# Spatial control of enzyme orientation at the lipid droplet interface enables regio- and chemoselective metabolic flux

**DOI:** 10.1126/sciadv.aeg5924

**Published:** 2026-11-06

**Authors:** So-Hee Son, Sangwon Bae, Soo Young Moon, Minhye Kim, Seung Soo Oh, Chang Yun Son, Ju Young Lee

**Affiliations:** ^1^ Graduate School of Engineering Biology, Korea Advanced Institute of Science and Technology (KAIST), Daejeon 34141, Republic of Korea.; ^2^ Department of Chemistry, Seoul National University, Seoul 08826, Republic of Korea.; ^3^ Department of Chemistry, Pohang University of Science and Technology (POSTECH), Pohang 37673, Republic of Korea.; ^4^ Department of Materials Science and Engineering, Pohang University of Science and Technology (POSTECH), Pohang 37673, Republic of Korea.; ^5^ Seoul National University Energy Initiative (SNUEI), Seoul National University, Seoul 08826, Republic of Korea.; ^6^ Department of Biological Sciences, Korea Advanced Institute of Science and Technology (KAIST), Daejeon 34141, Republic of Korea.; ^7^ KI forBioInnovation, Korea Advanced Institute of Science and Technology (KAIST), Daejeon 34141, Republic of Korea.

## Abstract

Precise spatial control over multienzyme cascade reactions is increasingly recognized as a design lever in synthetic biology for enhancing catalytic performance and directing metabolic flux. By emulating principles of cellular compartmentalization, synthetic scaffolds and engineered interfaces have emerged as powerful platforms to modulate intermediate channeling and reaction efficiency in complex biocatalytic systems. However, robust and spatially defined immobilization of multienzyme assemblies within physicochemically optimal compartments remains challenging because of the dynamic intracellular environment. Here, we reprogram the amphiphilic interface of yeast lipid droplets (LDs) as a programmable scaffold system to support regio- and chemoselective localization of enzymes. Guided by molecular dynamics simulations, we designed LD-anchoring motifs to direct the enzyme orientation at the LD monolayer interface, yielding cytosol-facing or LD inner-facing configurations. Using this designed LD-anchoring motif, we spatially reorganize a trienzyme cascade for prodeoxyviolacein (PDV) biosynthesis across the cytosol-LD interface, facilitating directional conversion of hydrophilic substrates into hydrophobic products. This spatial reorganization substantially improved PDV production by 1300%, achieving 46.25 milligrams per liter. Our approach offers a modular and tunable platform for engineering spatially organized and readily deployable enzyme cascades, opening avenues for biosynthetic catalysis at organellar interfaces.

## INTRODUCTION

Cellular metabolism often relies on the spatial organization of enzymes to efficiently orchestrate biochemical processes. In eukaryotes, lipid-bound organelles such as mitochondria and peroxisomes provide compartmentalized environments for biochemical reactions (*1*, *2*), while prokaryotes use membrane-less organelles such as protein-based condensates, including carboxysomes and metabolosomes, to create dedicated metabolic compartments (*3*, *4*). These compartments spatially colocalize sequential enzymes, streamlining cascade reactions and minimizing unwanted cross-talk with essential cellular pathways. Beyond their intrinsic roles, the spatial organization by both native and even artificial organelles has recently emerged as a powerful strategy in cellular and metabolic engineering. By encapsulating enzymes within favorable microenvironments, these compartments can protect proteins from degradation, enhance reaction kinetics, and mitigate the toxicity of unstable intermediates, ultimately supporting the efficient production of desirable metabolites (*5*, *6*).

Lipid droplets (LDs), unique organelles composed of a hydrophobic core of neutral lipids surrounded by a phospholipid monolayer, serve as specialized metabolic platforms with untapped potential (*7*, *8*). This amphiphilic ultrastructure not only facilitates the storage of hydrophobic metabolites but also offers a tunable interface for coordination and communication with the cytosol. Notably, many metabolic pathways cannot take place entirely within a single organelle, but they require metabolic flux across the cytosol-organellar interface. For example, the biosynthesis of poorly water-soluble metabolites, such as fatty acids and lipophilic drugs, necessitates a locally amphiphilic environment to efficiently channel hydrophilic intermediates into hydrophobic interiors. Lipid-based membranes, such as those of LDs, provide an ideal solution for spatially complex metabolic processes. Recent studies have demonstrated the potential of LDs as microenvironments for sequestering specific biomolecules, particularly in the biosynthesis of hydrophobic metabolites (*9*–*13*). However, despite their immense promise, the metabolic capacity of LDs and the intricate mechanisms regulating metabolic flux across the cytosol-LD interface remain underexplored.

To address these challenges, we developed a computational and experimental strategy to redesign LDs as dynamic enzyme scaffolds capable of achieving unparalleled efficiency in metabolic flux control (Fig. 1). In our approach, class I LD-anchoring motifs were redesigned through molecular dynamics (MD) simulations and functionalized for precise orientation control at the LD interface in vivo. Using the biosynthesis of hydrophobic prodeoxyviolacein (PDV) as a model pathway, we localized PDV metabolic enzymes to either cytosol-facing or LD inner-facing orientations at the LD interface in yeast, thereby enabling the efficient conversion of hydrophilic substrates into hydrophobic products within a spatially optimized framework. By fine-tuning the spatial positioning of PDV pathway enzymes at the LD interface, we achieved a 1300% increase in production, reaching 46.25 mg/liter. This study highlights our engineered LD scaffolds as a dynamic microenvironment for spatially tuning metabolic reactions in a controlled manner, optimizing metabolite-enzyme interactions, and revealing aspects that provide unprecedented control over metabolic processes.

**Fig. 1.**
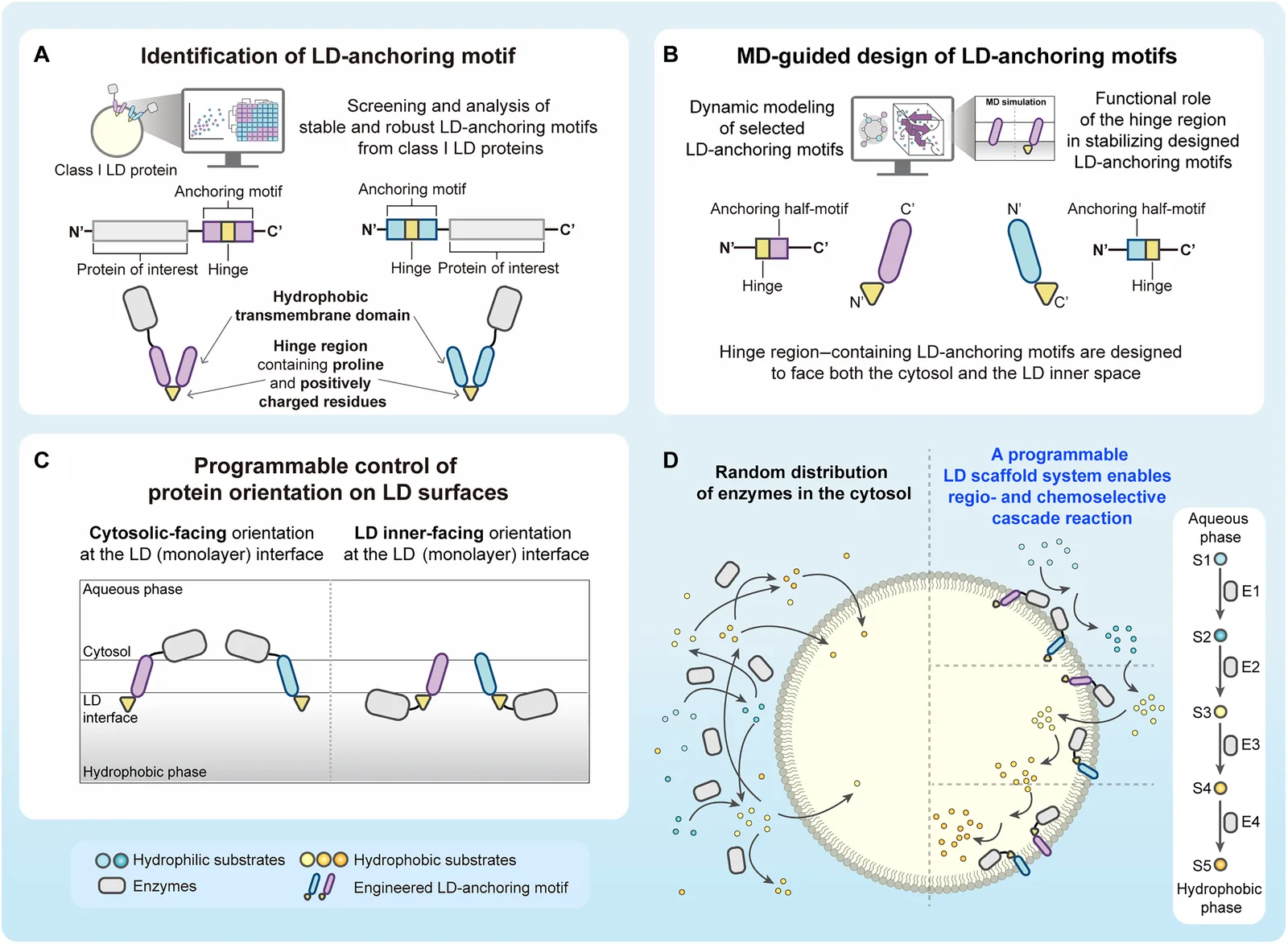
Schematic overview of the programmable LD scaffold system for spatial organization of metabolic reactions. (**A**) To identify suitable LD-anchoring motifs, class I LD membrane proteins are screened and analyzed on the basis of their high abundance and stable association with LDs. Class I LD proteins typically contain a membrane-embedded, V-shaped hydrophobic motif composed of two flanking hydrophobic regions and a central hinge region enriched in proline and positively charged residues. (**B**) LD-anchoring motifs are redesigned via MD simulations to enable precise control of protein orientation on the LD surface in vivo. MD simulations revealed the functional importance of the hinge region in stabilizing the designed LD-anchoring motifs. (**C**) The resulting hinge-containing LD-anchoring motifs conferred robust and spatially defined immobilization of target proteins, allowing them to function within physicochemically optimal microenvironments. (**D**) Using the programmable designed LD-anchoring motifs, enzymatic reactions are spatially reorganized and regulated in a controlled manner, enabling optimized metabolite-enzyme interactions across distinct intracellular environments such as aqueous cytosol or hydrophobic organellar interiors.

## RESULTS

### Identification of LD-anchoring motifs

LDs have a distinctive ultrastructure, consisting of an organic phase of neutral lipids, such as triacylglycerols (TAGs) and sterol esters, surrounded by a phospholipid monolayer (*14*, *15*). The LD surface, acting as an oil-water interface, is enriched with specific hydrophobic, membrane-embedded proteins, many of which are involved in lipid metabolism (*16*–*18*). However, the precise mechanisms by which newly synthesized proteins are targeted to LDs remain unknown. LD proteins are broadly classified into two classes: class I proteins, which are inserted into the endoplasmic reticulum and subsequently trafficked to LDs, where they stably associate with the LD membrane through hydrophobic motifs, and class II proteins, which are recruited from the cytosol and attach directly to the LD surface via amphipathic helices (fig. S1). Notably, a previous study has indicated that the sites available for class II protein insertion or association with LDs are spatially limited (*19*).

To identify stable and suitable LD membrane–anchoring motifs in a systematic way, we first screened and analyzed a set of 15 LD proteins that exhibit relatively high abundance (≥0.3%) on the LD surface, as informed by previous yeast LD proteomic analyses (table S1) (*20*). We were particularly interested in class I LD proteins to identify and engineer LD membrane–anchoring motifs that confer highly selective and stable localization of target proteins at the LD surface, allowing for either cytosol-facing or LD inner-facing orientations (Fig. 2A) (*16*–*18*). Using three well-established algorithms—Phobius, TMHMM, and Signal P-5.0—we predicted hydrophobic, membrane-embedded motifs from the 15 screened candidates and chose 10 proteins with distinct LD-anchoring motifs (table S2) (*21*–*23*). To enable site-specific linkage of target proteins via their N or C termini to the LD-anchoring motifs—while ensuring that the remaining free terminus is available for biological activity—we categorized the LD-anchoring motifs on the basis of their location. C-terminal LD motifs, including Erg6, Ayr1, Pgc1, Hfd1, and Erg1, have hydrophobic motifs at their extreme C termini, while N-terminal LD motifs, including Tgl3, Fat1, Loa1, Slc1, and Tgl1, have these motifs at their extreme N termini.

**Fig. 2.**
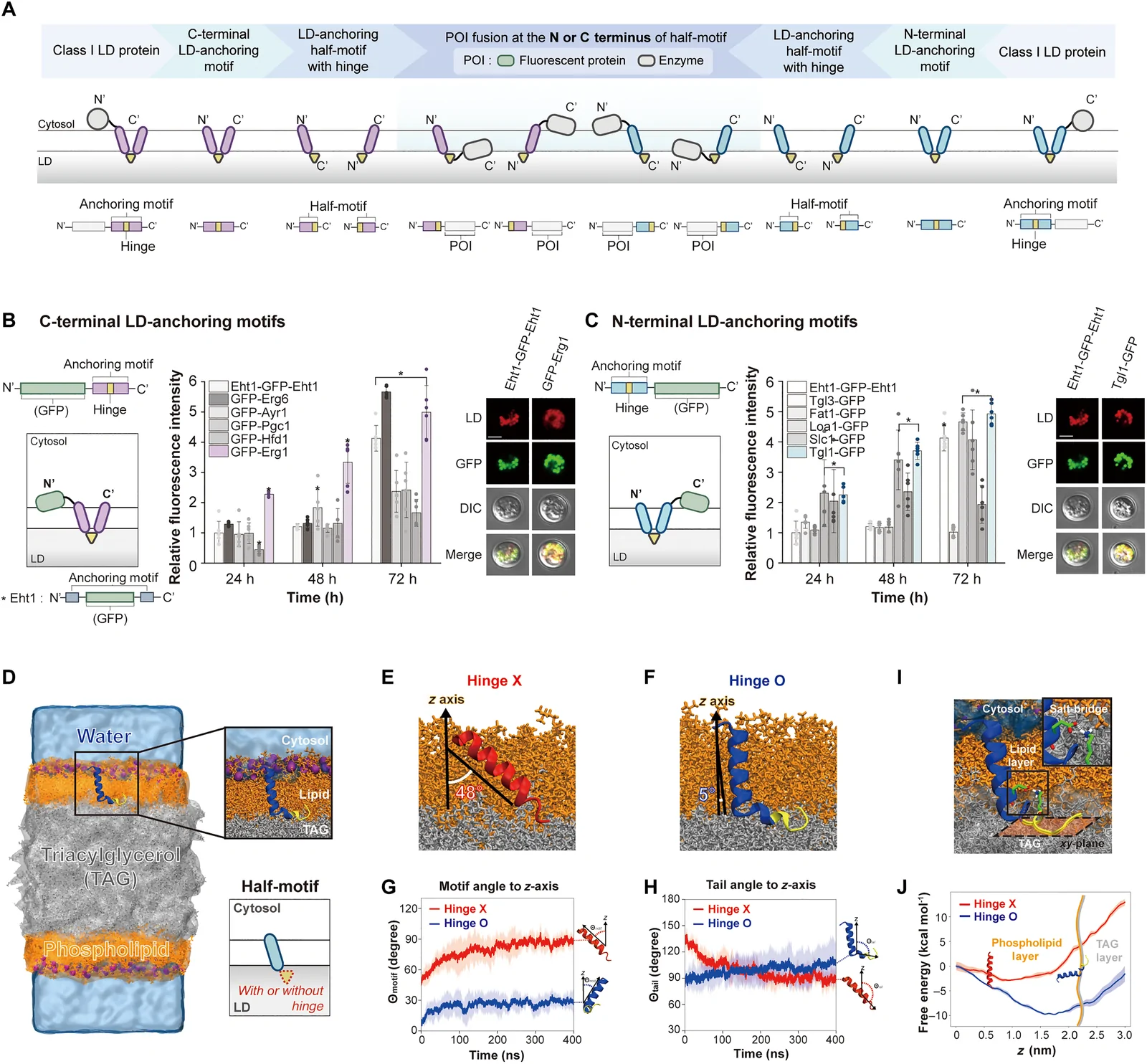
Molecular design of LD-anchoring motifs for LD surface localization. (**A**) Design strategy for constructing LD-anchoring motifs. A single hydrophobic region was removed from the V-shaped hydrophobic motif of class I LD proteins to generate a hinge-containing LD-anchoring half-motif, which was fused to either the N or C terminus of proteins of interest (POIs) for LD surface localization. (**B** and **C**) Screening of selective and stable LD-anchoring motifs derived from abundant yeast LD-associated proteins. We fused GFP to the N or C terminus of each predicted LD-anchoring motif and evaluated its localization by confocal fluorescence microscopy. Erg1- and Tgl1-derived motifs exhibited the highest LD surface anchoring capability, while the previously reported Eht1 motif served as a control. Yeast cells were grown in defined minimal media with 2% glucose at 30°C for 72 hours (h). Asterisks (*) indicate statistical significance compared with the Eht1-GFP-Eht1 control at 24 hours (*P* < 0.05, two-sided *t* test). GFP fluorescence intensities were quantified using ImageJ and normalized to the Eht1-GFP-Eht1 control at 24 hours. See fig. S2 for raw images. (**D**) Schematic of the LD trilayer system. Tgl1 half-motifs with (blue) or without (red) the hinge region were embedded in the upper phospholipid monolayer above the TAG core. (**E** and **F**) The hinge-less motif tilted and became peripherally associated, whereas the hinge-containing motif remained stably embedded in a vertical orientation. (**G** and **H**) The motif orientation was quantified by the α-helical and tail-region angles relative to the membrane normal axis. (**I**) The hinge region adopted an L-shaped conformation at the phospholipid-TAG interface, stabilized by a Glu-Lys salt bridge. (**J**) Effect of hinge region on the binding free energy of Tgl1 half-motifs to the phospholipid-LD core interface. Shaded regions indicate the statistical error estimated by five-block averaging.

Next, to examine the LD localization capabilities and expression levels of each LD-anchoring motif, we fused green fluorescent protein (GFP) to either the C or N terminus of 10 LD-anchoring motifs. These GFP-fused LD motifs were then expressed in wild-type yeast cells (Fig. 2, B and C; fig. S2; and table S3). The previously reported LD-anchoring motif of Eht1 was used as a control; Eht1, known as octanoyl-coenzyme A:ethanol acyltransferase, localizes to both LDs and the mitochondrial outer membrane via amino acids 1 to 13 and 429 to 451 (*24*). We observed that GFPs tagged with the C-terminal Erg1 motif (GFP-Erg1) and the N-terminal Tgl1 motif (Tgl1-GFP) were highly expressed and stably localized to the LD surface throughout the whole cultivation period (72 hours) (Fig. 2, B and C; fig. S2; and table S3). After 72 hours of cultivation, GFP-Erg1 and Tgl1-GFP exhibited 4.99- and 4.93-fold increases in fluorescence intensity compared to GFP tagged with the control Eht1 motif (Eht1-GFP-Eht1), respectively. Given their high and stable localization on the LD surface, we proceeded with the two effective LD-anchoring motifs (C-terminal Erg1 and N-terminal Tgl1) to anchor target proteins on the LD surface in an on-demand manner.

### Functional design of anchoring motifs for controlling protein orientation at the LD interface

To gain a sophisticated LD scaffold system, we further engineered the LD-anchoring motif of Erg1 (*12*) and Tgl1 (*25*). Our design strategy aimed to achieve precise and optimal enzyme immobilization on the LD surface, enabling spatial control of enzymatic cascade reactions on the basis of the regio- and chemoselectivity of substrates. Specifically, we sought to design LD-anchoring motifs that enable directional organization of target proteins at the LD interface, with controlled orientation toward either the cytosolic side or LD inner-facing side (Fig. 2A and fig. S3). The design strategy was informed by conceptual insights from previous studies (*26*–*32*) on the binding mechanisms of class I LD proteins. Typically, class I LD proteins are anchored to the LD membrane in a V-shaped orientation (*26*), where two hydrophobic segments are linked by a flexible hinge region enriched in proline and positively charged residues, enabling stable positioning at the LD interface (figs. S1 and S3A).

According to previous studies (*17*, *27*–*32*), the V-shaped motif of class I LD proteins require three essential components for LD targeting: (i) hydrophobic regions at both ends of the V shape, (ii) a proline residue positioned at the center of the hydrophobic region, and (iii) positively charged residues located in the hinge region (Fig. 2A and fig. S3A). Previously, a combined experimental and computational study demonstrated that stabilization of the V-shaped motif arises from a “snorkeling” behavior of positively charged residues, along with insertion of a bulky tryptophan side chain into the phospholipid monolayer (*26*, *33*). A more recent study showed that increased molecular rigidity can drive the localization of a terpenoid molecule that snorkels at the interface between the phospholipid monolayer and the LD core (*9*). We hypothesized that as long as the essential components of the LD-targeting motif remained intact, a truncated form of the LD-anchoring motif would still be capable of stable and functional localization at the LD membrane.

We first computationally designed two truncated variants of the Tgl1 LD-anchoring motifs to evaluate their anchoring functionality on the LD membrane. Both designed motifs contained the N-terminal transmembrane domain of Tgl1, but only one of the motifs included the hinge region with a conserved proline residue and a positively charged lysine residue (figs. S4 and S5 and table S4). Our motif design aimed to induce snorkeling of the hinge region at the LD surface by incorporating a positively charged lysine residue at the hinge, together with structural stiffness introduced by stabilizing salt bridging. By performing extensive MD simulations of these two designed Tgl1 motifs (designed Tgl1 half-motifs), we quantitatively analyzed their molecular interaction with the LD membrane (Fig. 2, D to J). Our computational membrane system in atomistic detail explicitly accounts for the aqueous cytosol, the LD core modeled as a TAG layer, and two phospholipid monolayers encapsulating the LD core to form membrane interfaces with cytosol (Fig. 2D). For each designed Tgl1 half-motif, with and without the hinge region, we embedded the motif in the phospholipid monolayer and equilibrated for 10 ns under an isobaric-isothermal (NPT) ensemble. After equilibration, two sets of six independent 400-ns simulations were performed in an isothermal-isochoric (NVT) ensemble, again with and without the hinge. These simulations were subsequently analyzed to characterize the behavior of the Tgl1 half-motif at the LD interface.

Intriguingly, both of the designed Tgl1 half-motifs were stabilized at the phospholipid membrane of the LD surface, but the presence of the hinge region made a substantial difference in their anchoring structure and binding strength. In the absence of the hinge region, the transmembrane domain quickly tilted by 40° to 50° to the membrane normal axis, accommodating the longer α-helical domain within the phospholipid monolayer (Fig. 2E) (*26*). This tilt angle continued to grow until the designed motif forms a peripheral orientation, which was stabilized by charged terminal residues and a glutamate ion at the end of the helical domain interacting with water and lipid head groups. In contrast, the hinge-containing designed Tgl1 half-motif formed a near-vertical orientation, with a tilt of only ∼20° to the membrane normal axis (Fig. 2, E to H). The angle trace that marked every 10 ps represents the mean over six replicas initiated from different configurations. The shaded region denotes the standard deviation.

This difference in embedding structure originated from two key molecular interactions induced by the hinge region, providing strong anchoring stability to the hinge-containing designed half-motif on the LD membrane. First, there was a strong electrostatic interaction and hydrogen bonding between the negatively charged glutamate residue near the bottom of the transmembrane domain and the positively charged lysine residue in the hinge region (Fig. 2I). Second, the hinge region preferentially localized to the interface between the phospholipid monolayer and the LD core, resulting in a parallel orientation to the membrane surface. Together, these interactions collectively influenced the hinge-containing designed half-motif to form a tightly bound L-shaped conformation at the membrane layer, in which the transmembrane domain was vertically embedded, while the hinge region aligned along the LD core-monolayer interface, forming an equatorial tail orientation. This secondary LD core-monolayer interface has been reported as a preferential binding site for structurally rigid terpenoid molecules (*9*), suggesting that the hinge region may mimic such interactions, further stabilizing our designed half-motifs. Both of the key molecular interactions are preserved in the C-terminal Erg1 LD-anchoring half-motif as well, implying its functional importance in stabilizing the designed half-motif on the LD surface. Moreover, free energy calculations confirmed that the hinge-containing designed half-motif binds favorably to the LD membrane, with a binding free energy of −9.75 kcal/mol, which is 6.85 kcal/mol lower (more favorable) than that of the hinge-less designed half-motif (Fig. 2J). Umbrella sampling window overlap and block-averaging analyses further confirmed sufficient sampling coverage and convergence of the free energy profile (fig. S6).

### Designed LD-anchoring motifs control target protein orientation at the LD interface

To experimentally validate the MD findings of our designed half-motifs (hereafter referred to as designed LD-anchoring motifs), we assessed their ability to stably and directionally localize target proteins at the LD interface in living yeast cells (Fig. 2A and fig. S3). We selected GFP as a model target protein and incorporated the hinge-containing designed LD-anchoring motifs, flanking the GFP with a flexible glycine-serine linker between the GFP and the designed LD-anchoring motifs.

We first fused GFP to LD-anchoring motifs designed to orient the protein toward either the cytosolic side (CYT) or the LD inner-facing side (L) at the LD interface using motifs derived from both Erg1 and Tgl1 (Fig. 3 and fig. S7). Specifically, GFP was tagged to the N and C termini of the cytosol-facing Erg1 (Erg1^CYT^) and Tgl1 (Tgl1^CYT^) motifs, while GFP was similarly tagged to the N and C termini of the LD inner-facing Erg1 (Erg1^L^) and Tgl1 (Tgl1^L^) motifs. We ensured that GFP-Erg1^CYT^, Tgl1^CYT^-GFP, GFP-Erg1^L^, and Tgl1^L^-GFP were expressed and localized to the right orientation at the LD interface in wild-type yeast cells, as directed by the designed LD-anchoring motifs.

**Fig. 3.**
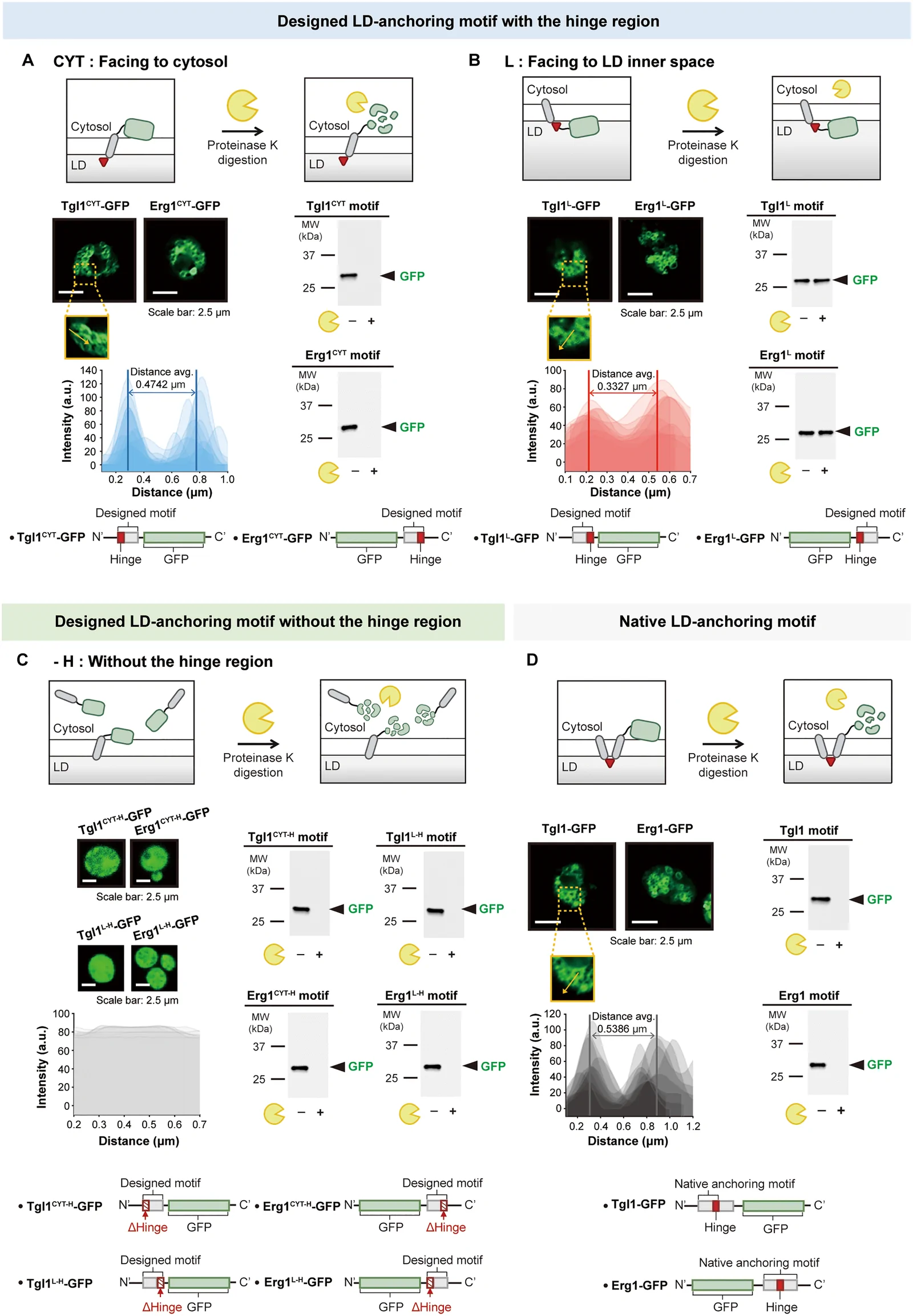
Spatial control of target protein orientation at the LD interface via designed LD-anchoring motifs. We assessed the ability of the designed LD-anchoring motifs derived from Erg1 and Tgl1 to direct and stably anchor target proteins at the LD interface with defined orientation in yeasts. GFP was fused to each LD-anchoring motif to orient GFP toward the cytosolic-facing side (**A**) or the LD inner-facing side of the LD interface (**B**). The hinge-containing designed LD-anchoring motifs successfully localized GFP to the LD surface with controlled orientation. The localization and orientation of GFP at the LD interface were analyzed using proteinase K digestion assays and confocal fluorescence microscopy. Yeast lysates expressing the GFP fused with the designed LD-anchoring motif were treated with proteinase K and analyzed by Western blotting using a GFP-specific antibody. Cytosol-facing GFPs (GFP-Erg1^CYT^ and Tgl1^CYT^-GFP) were exposed and degraded by the proteinase, whereas LD inner-facing GFPs (GFP-Erg1^L^ and Tgl1^L^-GFP) remained protected and intact. Supporting this controlled orientation via the designed LD-anchoring motifs, a slight difference in the diameter of the GFP fluorescence ring was observed between cytosol-facing and LD inner-facing GFPs localized at the LD interface. a.u., arbitrary units; MW, molecular weight. (**C**) The hinge-less designed LD-anchoring motifs (Tgl^CYT-H and L-H^ and Erg1^CYT-H and L-H^) failed to localize to the LD surface and were retained in the cytosol, indicating that the hinge region is essential for stable LD anchoring. (**D**) Native LD-anchoring motifs of Erg1 and Tgl1 localized correctly to the LD surface but lacked the structural specificity to orient GFP toward the LD inner space. Yeast cells were grown in defined minimal media with 2% glucose at 30°C for 48 hours. For confocal imaging, GFP was excited at 488 nm, and emission was collected between 500 and 530 nm. Scale bars, 2.5 μm. See fig. S7 for the corresponding repeated raw-image datasets.

To determine the orientation of GFP at the LD interface, we then used a common protease-based analysis (*34*) using proteolytic digestion of the accessible outward-facing portions by a nonspecific protease, such as proteinase K (*35*–*39*). After treatment of the yeast lysates expressing GFPs fused to the designed LD-anchoring motifs with the protease, we observed that the cytosol-facing GFPs (GFP-Erg1^CYT^ and Tgl1^CYT^-GFP) were exposed and subsequently degraded, while the LD inner-facing GFPs (GFP-Erg1^L^ and Tgl1^L^-GFP) remained intact (Fig. 3, A and B, and fig. S7). In the absence of proteinase treatment, all LD motif–fused GFPs remained intact. In support of the controlled orientation of GFP at the LD interface by the designed LD-anchoring motifs, we also observed a slight difference in the diameter of the fluorescence ring formed by GFP depending on its orientation (Fig. 3, A, B, and D, and fig. S7). Moreover, time-lapse fluorescence imaging of digitonin-permeabilized spheroplasts revealed a rapid loss of fluorescence in the cytosol-facing GFPs upon proteinase K treatment, while the fluorescence of the LD inner-facing GFPs remained intact, in line with the Western blot results (fig. S8 and movies S1 and S2).

Moreover, we further clarified whether the hinge region of the LD-anchoring motifs contributes to their energetically stable anchoring at the LD interface. In line with our MD simulation, the designed LD-anchoring motifs lacking the hinge regions (Tgl1^CYT-H and L-H^ and Erg1^CYT-H and L-H^) failed to anchor to the LD surface and were instead retained in the cytosol, indicating that the hinge region is essential for stable LD anchoring (Fig. 3C and fig. S7). In addition, MD simulations of Tgl1^L^-GFP positioned at the LD surface revealed that the L-shaped binding of the Tgl1^L^ motif supports stable positioning of GFP while maintaining its structural integrity (fig. S9). This alignment highlights the role of the hinge-containing designed LD anchoring motif in supporting thermodynamically stable anchoring and enabling precise spatial localization of target proteins, such as GFP, at the LD interface. Our MD simulation and experimental results in cells confirmed that the designed LD-anchoring motifs enable robust, orientation-specific localization of target proteins at LD surfaces in living cells.

### The designed LD-anchoring motifs enable functional positioning of enzymes at the LD interface

Given the modularity and programmability of our designed LD-anchoring motifs, they can selectively deliver and anchor target proteins to the LD interface, effectively transforming LDs into programmable organellar scaffolds for the spatial reorganization of metabolic pathways. Such reorganization on LD compartments would be a highly desirable synthetic biology strategy, as it enables the separation of non-natural pathways and processes into favorable biochemical environments—such as the aqueous phase in the cytosol or the hydrophobic phase in the LD inner space—while also allowing locally high concentrations of key pathway components to support enzymatic catalysis.

To explore this potential, we evaluated whether our designed LD-anchoring motifs could facilitate enzymatic cascade reactions by spatially directing functional enzymes to distinct LD surfaces. For controlling metabolic pathways on the LD outer and inner surfaces, we selected the well-characterized three-enzyme PDV pathway as a model metabolic pathway (Fig. 4A), which proceeds from a hydrophilic substrate through progressively hydrophobic intermediates to a hydrophobic product—making it an ideal model for environment-driven spatial organization. More specifically, the PDV pathway, encoded by the *vioA*, *vioB*, and *vioE* genes from the bacterium *Chromobacterium violaceum*, converts hydrophilic tryptophan into the hydrophobic green pigment PDV (*40*, *41*). We hypothesized that positioning the first two enzymes (VioA and VioB) with cytosolic activities in the cytosolic compartment, while directing the final hydrophobic metabolite-producing enzyme (VioE) toward the hydrophobic space of LDs, would optimize the PDV reaction flux by channeling substrates, intermediates, and final products to their most favorable reaction environments.

**Fig. 4.**
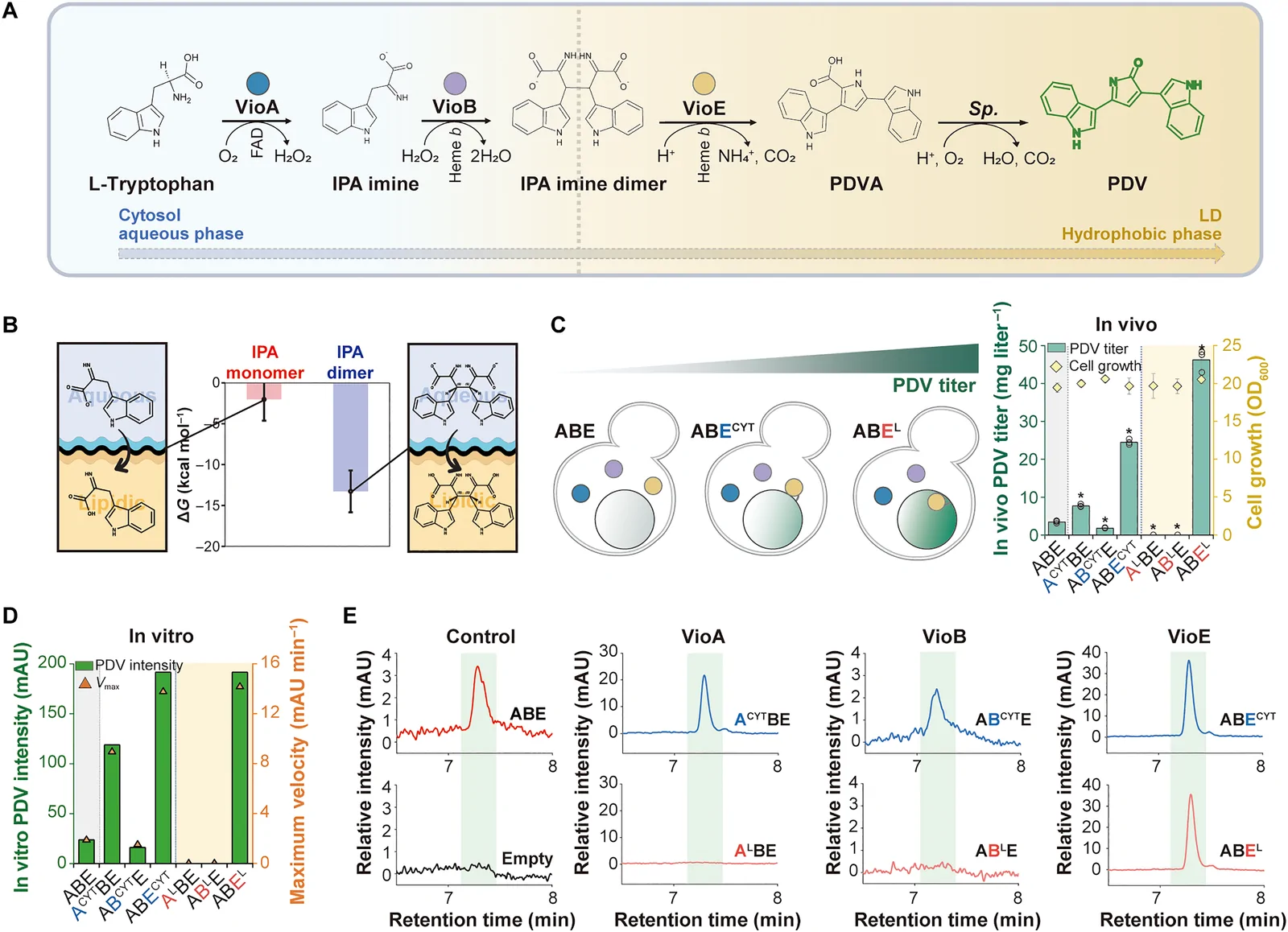
Engineering LDs into programmable organellar scaffolds for spatial organization of metabolic pathways. (**A**) Biosynthetic PDV pathway from hydrophilic tryptophan to hydrophobic PDV, spanning the aqueous cytosol and hydrophobic LD inner space through progressively hydrophobic intermediates. The pathway involves three enzymatic steps (VioA, VioB, and VioE from *C. violaceum*), followed by spontaneous rearrangement. (**B**) MD-calculated transfer free energies of IPA imine and IPA imine dimer intermediates in the PDV pathway. The IPA imine dimer strongly preferred the LD inner space over the cytosol. The error bars represent statistical errors estimated by 20-block averaging. (**C**) Spatial organization of PDV biosynthetic enzymes at the LD interface using designed LD-anchoring motifs. Anchoring VioE on the LD surface (ABE^CYT^ and ABE^L^) significantly enhanced PDV production. Yeast strains were cultivated in 10 ml of defined minimal media containing 2% glucose, l-tryptophan (608 mg/liter), and 40 μM ammonium iron(II) sulfate for 144 hours at 30°C in a 50-ml bioreactor. All PDV production experiments were repeated at least three times independently. (**D**) In vitro PDV biosynthesis using spatially organized VioA, VioB, and VioE. Consistent with in vivo PDV synthesis, ABE^CYT^ and ABE^L^ lysates produced PDV most efficiently and exhibited the highest catalytic activity. *V*_max_ was calculated from the initial linear slope (0 to 10 min). Reactions used 100 µg of yeast lysate protein in 5-ml reaction buffer, and PDV intensity was measured at 10 min. (**E**) HPLC analysis of in vitro–produced PDV using spatially organized VioA, VioB, and VioE. PDV peaks matched the standard retention time (∼7.3 min), with ABE^CYT^ and ABE^L^ yielding the highest PDV peaks. All data represent the means of biological triplicates, and error bars indicate standard deviation. Asterisks (*) denote statistical significance compared with the cytosolic ABE control (*P* < 0.05, two-sided *t* test).

To test this hypothesis, we first analyzed the solvation preference of each metabolite in the PDV pathway, including the substrate l-tryptophan, the two key intermediates indole-3-pyruvic acid (IPA) imine and IPA imine dimer, and the final product PDV, as it is essential to understand the physicochemical preferences of each metabolite to determine the optimal positioning of each enzyme for the most efficient cascade reaction. While l-tryptophan is known to be hydrophilic and PDV to be strongly hydrophobic, the solvation preference of the two intermediates (IPA imine and IPA imine dimer) remains poorly characterized. To quantify their relative hydrophobicity and characterize their preferred localization between the aqueous cytosol and the LD core as a TAG layer, we performed transfer free energy calculations (Fig. 4B). The IPA imine monomer and IPA imine dimer exhibited transfer free energies of −2.02 and −13.27 kcal/mol, respectively. The error bars represent the statistical error estimated by 20-block averaging. The simulations revealed that the monomeric IPA imine exhibited a relatively equal preference for both the cytosol and LD inner space, whereas the IPA imine dimer showed a strong preference for the LD inner space over the cytosol by 13.27 kcal/mol, indicating its significantly higher affinity for the hydrophobic environment. Consistent with these findings, a previous study reports that VioA—the initiating enzyme in the PDV pathway that oxidizes the hydrophilic substrate l-tryptophan—functions optimally in the aqueous cytosolic phase (*42*). VioB activity is favored at high pH (pH ∼8.5), while VioE catalysis depends on proton availability, implying changes in the charge state of its imine-based substrate intermediates (*42*–*44*). Combined with our computational transfer free energy analysis, this supports a spatial configuration in which VioE is anchored at the LD interface—positioned to access the hydrophobic IPA imine dimer partitioned toward the LD inner space. Meanwhile, positioning VioA and VioB in the cytosolic phase would provide the most effective strategy for an optimal cascade reaction.

Guided by our MD simulation results, to achieve LD anchoring of VioE in vivo, we fused the designed LD-anchoring motifs (N-terminal Tgl1^CYT^ and Tgl1^L^) to the N terminus of VioE, as previous studies have shown that modifications to the N-terminal region of VioE do not affect its folding or activity (table S5) (*45*–*47*). Similarly, we fused the designed C-terminal Erg1 motifs (Erg1^CYT^ and Erg1^L^) to the C terminus of VioA and VioB (table S5) (*46*). All three enzymes were localized at the LD interface, with defined orientations toward either the cytosolic-facing or LD inner-facing side in both wild-type and our LD-engineered yeast cells [LD-size yeast cell from our previous study (*9*)] (Fig. 4C, figs. S10 and S11, and tables S6 and S7). The LD-engineered cell—with expanded LD surface area (table S8)—provided increased capacity for protein anchoring compared to the wild-type cell, enabling more distinct visualization and functional assessment of LD-anchoring effects across multiple enzymes. We thus used the LD-engineered cell hereafter for evaluating the spatial organization and functional performance of multienzyme anchoring at the LD interface.

As expected, yeast cells with VioE oriented toward the LD inner space (ABE^L^) demonstrated significantly increased PDV production (Fig. 4C and table S7). After 144 hours of cultivation, ABE^L^ cells produced 46.25 mg/liter PDV—a 1340% increase compared to the cytosolic control ABE cells. In contrast, the cytosolic control cells expressing VioABE in the cytosol (ABE) produced only 3.45 mg/liter PDV. The ABE^CYT^ yeast cells, with cytosolic-facing VioE at the LD interface, also exhibited increased PDV production, reaching 24.49 mg/liter after 144 hours—a 709% increase over the cytosolic control cells (ABE). Anchoring the first enzyme VioA to the cytosolic space at the LD interface (A^CYT^BE) enhanced PDV production to 7.71 mg/liter, which was a 223% increase over the cytosolic control cells (ABE). However, PDV production by the AB^CYT^E cells (1.83 mg/liter) was lower than that of the cytosolic control ABE cells. In addition, we found that when enzymes were oriented toward the LD inner space—an unfavorable hydrophobic environment—as in the A^L^BE and AB^L^E configurations, PDV production was not observed, suggesting that VioA and VioB facing the LD inner space would likely lack access to their hydrophilic substrates (l-tryptophan and IPA imine).

It is likely that the concentration of the initial enzyme (VioA) at the confined reaction space of the LD surface could boost the initial rate of the reaction cascade. Previous studies have reported that immobilizing enzymes at the first reaction step can enhance kinetics (*48*, *49*), although the underlying mechanisms remain unclear. Furthermore, localizing the final PDV-producing enzyme, VioE, at the LD interface may facilitate the rapid diffusion of hydrophobic PDV across the LD membrane, allowing efficient accumulation within the LD inner space. In contrast, not all enzymes are equally robust to the LD interfacial environment. For example, VioB exhibited only marginal activity in the cytosol-facing orientation and showed nearly no activity in the LD inner-facing configurations, resulting in no improvement in PDV production upon LD anchoring (Fig. 4C). This result suggests that certain enzymes may be inherently sensitive to the LD interfacial environment, potentially due to folding instability or interfacial perturbation, which may limit their compatibility with LD-associated localization.

To further clarify the effect of anchoring PDV-producing enzymes at the LD interface, we tested additional PDV biosynthesis in vitro using the engineered LD yeast cell lysates (Fig. 4, D and E). Corroborating in vivo PDV synthesis, yeast lysates with VioE anchored at the LD interface (ABE^CYT^ and ABE^L^) produced PDV most efficiently (Fig. 4, D and E, and table S9). Consistently, maximum velocity (*V*_max_) values in ABE^CYT^ and ABE^L^ lysates increased to 13.76 and 14.16 milli–absorbance units (mAU)/min, respectively, compared to the control ABE lysate (1.87 mAU/min) (Fig. 4D and table S9). We noted that ABE^CYT^ lysate could achieve PDV productivity similar to ABE^L^, likely due to the greater availability of substrates and cofactors under substrate- and cofactor-saturated in vitro conditions compared to in vivo cells.

To further extend the applicability of our designed LD-anchoring motifs for spatial enzyme control, we applied them to the lycopene biosynthetic pathway composed of *Xanthophyllomyces dendrorhous* CrtE, CrtB, and CrtI (fig. S12A and table S10). This cascade reaction converts hydrophilic intermediates—farnesyl pyrophosphate and geranylgeranyl pyrophosphate—into the highly hydrophobic end products phytoene and lycopene. To assess the effect of enzyme orientation at the LD interface, each enzyme (CrtE, CrtB, and CrtI) was anchored in either the cytosol-facing or LD inner-facing configuration using our CYT- and L-facing LD-anchoring motifs.

Consistent with the spatial localization trends observed in PDV production (Fig. 4), we observed that enzyme localization had a significant effect on lycopene biosynthesis, likely reflecting the physicochemical preferences between enzyme positioning and their corresponding substrates, intermediates, and products (fig. S12B and tables S10 and S11). Specifically, anchoring CrtI in the LD inner-facing configuration (EBI^L^) significantly enhanced lycopene accumulation to 1.66 mg/g dry cell weight—a 2.44-fold increase compared to the cytosolic configuration (EBI, expressing CrtE, CrtB, and CrtI in the cytosol). Likewise, the cytosol-facing anchoring of CrtE (E^CYT^BI) increased lycopene production (0.92 mg/g dry cell weight; a 1.34-fold increase over the cytosolic control EBI). Accordingly, in line with the PDV production results, localizing the initial enzyme CrtE to the cytosolic phase at the LD interface likely facilitates the initial step of the biosynthetic cascade, thereby increasing the overall pathway flux. In contrast, positioning the final enzyme CrtI at the LD interface with the LD inner-facing orientation may enhance the efficient conversion and accumulation of the hydrophobic intermediate and product. To further validate hinge-dependent stable LD anchoring, we performed in vitro PDV and geranylgeranyl pyrophosphate biosynthesis assays using VioE and CrtE fused with the designed LD-anchoring motifs lacking the hinge region (VioE^CYT-H^ and CrtE^CYT-H^) (fig. S13 and tables S12 and S13). Consistent with the localization trends observed in Fig. 3C, deletion of the hinge region caused both VioE and CrtE to fail to stably anchor at the LD interface, resulting in biosynthetic outputs comparable to those of the cytosolic controls. These results further demonstrate that the hinge region is essential for stable LD anchoring.

Together, these results suggest that the physicochemical redistribution of a metabolic reaction into an appropriate environment can increase catalytic efficiency as well as enhance metabolic flux by concentrating enzymes and intermediate metabolites in close proximity, thereby accelerating the metabolic reaction rate. At the same time, given that the LD interface is a physicochemically distinct environment that could potentially affect protein folding or stability, enzyme-specific design may be required to achieve effective spatial organization.

### Cooperative anchoring of multiple enzymes at the LD interface for enhanced metabolic flux control

We further evaluated the anchoring of multiple enzymes onto LD surfaces to achieve spatial organization of metabolic pathways within a favorable environment. We targeted two or three enzymes simultaneously to the LD surfaces, exploring configurations in which two or all three enzymes (VioA, VioB, and VioE) were colocalized at the LD interface in various combinations and oriented either toward the cytosol or the LD inner space. Unexpectedly, when two or three enzymes were anchored simultaneously, the PDV titer was lower both in vivo and in vitro compared to that achieved with a single enzyme anchored at the LD interface (Fig. 5A, fig. S11, and table S7). Even with the most efficient configuration, simultaneous anchoring of cytosol-facing VioA and LD inner-facing VioE (A^CYT^BE^L^) produced 63% less PDV compared to the best PDV-producing ABE^L^ in vivo. These results suggest that the LD surface capacity for multiple enzymes is still a limiting factor, restricting the amount of functional protein that can be localized on the LD surface. Supporting this hypothesis, we found that expanding the available LD surface area by supplementing with oleic acid, which increases the number and size of LDs by promoting lipid accumulation (*50*, *51*), partially alleviated these limitations and enhanced PDV production (fig. S11 and table S7).

**Fig. 5.**
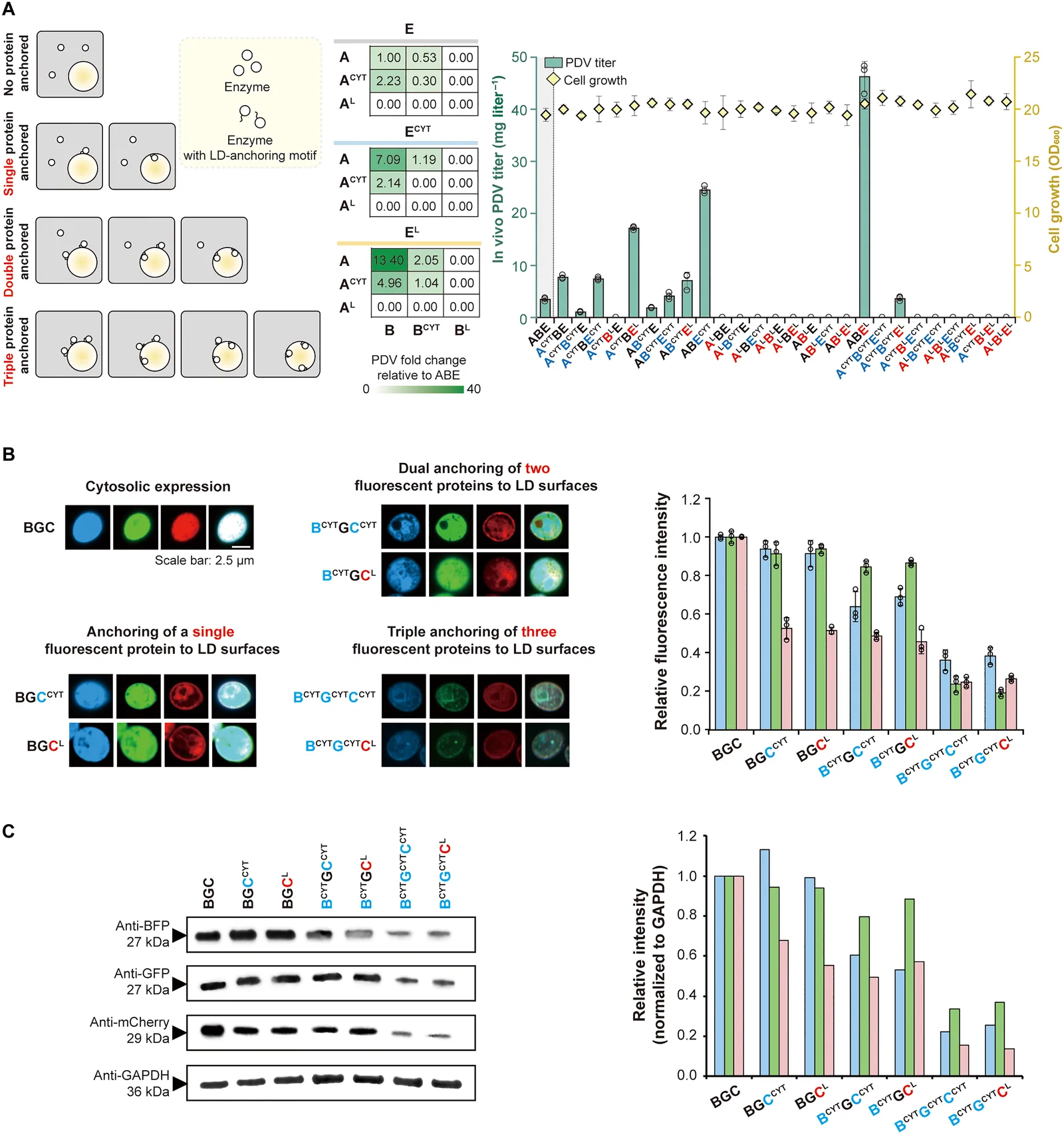
Spatially coordinated anchoring of multiple proteins on LD surfaces. (**A**) Anchoring of multiple enzymes onto LD surfaces to spatially organize the PDV biosynthetic pathway. Enzymes (VioA, VioB, and VioE) were simultaneously anchored in various cytosol- and LD inner-facing orientations using the designed LD-anchoring motifs. Anchoring a single enzyme (VioE) unexpectedly yielded higher PDV production than simultaneous multienzyme anchoring, even in the most favorable configuration (A^CYT^BE^L^). The heatmap shows PDV titers (mg/liter) and fold changes relative to the cytosolic control (ABE). Yeast strains were cultivated in 10 ml of defined minimal media containing 2% glucose, l-tryptophan (608 mg/liter), and 40 μM ammonium iron(II) sulfate for 144 hours at 30°C in a 50-ml bioreactor. Experiments were repeated at least three times independently. (**B**) Simultaneous localization of three fluorescent proteins (GFP, BFP, and mCherry) fused with the designed LD-anchoring motifs on LD surfaces. The fluorescence intensity decreased by up to 81% when all three proteins were simultaneously anchored. Yeast cells were grown in defined minimal media with 2% glucose at 30°C for 48 hours. For confocal imaging, BFP, GFP, and mCherry were excited at 405, 488, and 587 nm, and emission was collected at 440 to 460, 500 to 530, and 610 to 620 nm, respectively. Fluorescence intensities were quantified using ImageJ and normalized to the corresponding cytosolic controls. See fig. S14 for raw images. (**C**) Western blot analysis of GFP, BFP, and mCherry under simultaneous anchoring of one, two, or three proteins on LD surfaces. The abundance of each protein progressively decreased as the number of coanchored proteins increased, consistent with the fluorescence analysis. Protein expression levels were analyzed using anti-BFP, anti-GFP, and anti-mCherry antibodies, and GAPDH was used as a loading control. Quantified band intensities, normalized first to GAPDH and subsequently to the respective cytosolic controls, are shown on the right.

To further verify this assumption, we used three fluorescent proteins [GFP, blue fluorescent protein (BFP), and mCherry] fused with the designed LD-anchoring motifs and simultaneously localized them at the LD interface (Fig. 5B). We observed a consistent and significant decrease in fluorescence when all three fluorescent proteins were simultaneously anchored on LD surfaces, where a drop of up to 81% in fluorescence levels was observed (Fig. 5B, fig. S14, and table S14). However, given the inherent limitations of confocal microscopy in quantitatively assessing protein colocalization at LD surfaces, accurate quantification of fluorescent protein abundance and spatial distribution remained limited because of insufficient spatial resolution. In parallel, we performed Western blot analysis to assess the abundance of LD-anchored fluorescent proteins under conditions in which one, two, or three fluorescent proteins were simultaneously anchored. Consistent with the fluorescence analysis, the abundance of each individual protein progressively decreased as the number of coanchored proteins increased (Fig. 5C, fig. S15, and table S15). These results suggest that the available LD surface area is limiting and that proteins compete for limited anchoring capacity.

Collectively, these findings suggest that artificially targeting all two or more enzymes to the LD interface remains challenging, possibly due to the limited LD surface capacity and the presence of essential endogenous proteins vital for cell survival within the LD surface. Thus, optimizing the available LD space for multiple proteins may better cope with our designed LD-anchoring motifs, significantly improving metabolic flux at the LD interface.

## DISCUSSION

When assembling enzymes nearby for substrate channeling, confining enzyme-substrate reactions within an appropriate microenvironment is necessary for both catalytic function and efficient substrate transfer. This spatial organization enhances catalytic efficiency and improves metabolic flux, ultimately leading to higher productivity of desired products. However, strategies for precisely manipulating and optimizing these spatial and functional traits in enzymatic reactions are far less reported. Here, we set out to develop an innovative LD scaffold system as a customizable and tunable microenvironment for spatially organizing metabolic reactions in a controlled manner. By introducing a rationally designed LD-anchoring motif, we enabled the targeted localization of heterologous proteins to the yeast LD surface with a controlled orientation in an on-demand manner.

To realize this concept, we computationally and experimentally designed and validated the customizable LD-anchoring motifs that facilitate enzyme anchoring at the LD interface, imposing controlled orientation toward either the cytosolic-facing or LD inner-facing side. Our designed motif used highly controlled specific interaction with the phospholipid monolayer and interfacial lipid environment, mediating stable, orientation-defined positioning at the amphiphilic LD interface. Beyond previous studies that merely focused on compartmentalization within cellular organelles (*1*, *52*), this tunable LD-anchoring system allows the precise localization of enzymes in multistep cascade reactions, thereby optimizing regio- and chemoselectivity to enhance catalytic performance (note S1). In other words, our LD-anchoring system may contribute to the spatial redistribution of metabolic reactions by decoupling hydrophilic and hydrophobic processes, thereby enabling LDs to serve as programmable reaction scaffolds for metabolic pathway reorganization.

Exploiting this concept, we successfully localized three PDV metabolic enzymes to either the cytosolic-facing or LD inner-facing side of the LD interface, which led to a significant enhancement in PDV pathway efficiency. By computationally predicting preferential localization of intermediates along the pathway, we strategically positioned the final PDV-producing enzyme, VioE, toward the LD inner-facing side of the LD interface to maximize its interaction with the highly hydrophobic IPA imine dimer substrate. This approach led to a substantial increase in PDV titer, achieving 46.25 mg/liter at the 50-ml bioreactor scale, highlighting the potential of our LD-anchoring system in optimizing metabolic flux by precisely orchestrating enzyme localization within cellular compartments. Still, not all enzymes exhibited equal compatibility with the LD interfacial environment. For example, VioB showed only marginal activity in the cytosol-facing orientation and nearly no activity in LD inner-facing configurations in both in vivo and in vitro experiments, resulting in no improvement in PDV production upon LD anchoring. These findings suggest that enzyme performance at the LD interface varies among enzymes and is influenced by their physicochemical properties. Accordingly, while our results demonstrate that spatial organization at the LD interface can enhance pathway efficiency through orientation-dependent substrate accessibility and physicochemical partitioning, successful implementation may require enzyme-specific optimization tailored to individual protein properties.

Moreover, further improvement of the customizable LD-anchoring efficiency will require a comprehensive understanding of how metabolic plasticity influences organelle properties. Although key physicochemical properties of LDs, such as lipid composition, appear to be relatively conserved under standard conditions (*9*), more dynamic or extreme cellular states—such as nutrient limitation or shifts in lipid metabolism—may alter LD morphology and interfacial properties, potentially compromising anchoring robustness. Thus, engineering organelle-intrinsic properties such as morphology, capacity, and protein insertion machinery may be necessary to ensure robust and consistent anchoring across diverse metabolic backgrounds. This includes optimizing the LD size, number, and volume to enhance the accommodation space for anchoring target proteins while minimizing any deleterious perturbation to native LD enzyme function, which remains an important area for future investigation.

The application of our designed LD-anchoring system extends beyond PDV production, encompassing biosynthesis pathways for hydrophobic nutraceutical products and therapeutic drugs. In addition, it functions as a hub that facilitates enzyme-substrate interactions, further broadening its applicability in metabolic engineering and synthetic biology. Given the tunability and modularity of our approach, LDs could serve as programmable biochemical compartments, enabling the separation and spatial organization of distinct enzymatic pathways. Overall, our LD-anchoring system–mediated spatial reorganization of metabolic pathways thus provides insights and design concepts for adopting cellular organelles as versatile tools in synthetic biology.

## MATERIALS AND METHODS

### MD simulation

All MD simulations were performed using OpenMM version 8.0 (*53*). The CHARMM36 (*54*, *55*) force field was used for phospholipids, TAGs representing LD cores, proteins, and ions, along with a modified TIP3P water model (*56*). In particular, the partial charges of the TAG headgroup in the LD system were adopted from the previously reported C36c parameter set (*57*), which has been shown to reproduce hydrophobic and interfacial properties of TAG with greater accuracy (*58*, *59*). Specifically, these superior descriptions enabled accurate prediction of the subtle structural coupling between the neutral lipid core and the phospholipid monolayer. Such high-fidelity parameterization is essential for capturing critical interfacial phenomena, as demonstrated by recent studies that successfully elucidated the regulation of surface packing defects by membrane remodeling (*60*) and the modulation of interfacial properties by core polarity (*61*). Compatible force field parameters for the intermediate substrates in the PDV pathway (i.e., IPA imine and IPA imine dimer) were obtained using the CHARMM General Force Field (*62*). The particle mesh Ewald method (*63*, *64*) was used to treat the long-range electrostatics, with a cutoff distance of 1.2 nm used for direct Coulomb and van der Waals interactions. The temperature and pressure of the system were maintained at 300 K and 1 atm using the Langevin thermostat and Monte Carlo Membrane barostat, respectively.

Preparation of the LD-like membrane environment followed protocols established in prior work (*9*). Briefly, a preequilibrated 10-nm-thick TAG layer was inserted between the upper and lower hydrated phospholipid monolayers consisting of a 60:40 ratio of 1-palmitoyl-2-linoleoyl-*sn*-glycero-3-phosphatidylcholine to 1,2-dipalmitoleoyl-*sn*-glycero-3-phosphoethanolamine. The equilibrated lipid monolayers and TAG layer were adapted from our previous work, where they were prepared using the CHARMM-GUI membrane builder tool (*65*, *66*). For membrane-embedded proteins, the initial structures of Erg1 and Tgl1 half-motifs were generated by AlphaFold2 (*67*) and then equilibrated in a 0.15 M KCl solution for a short time (1 ns) to model physiological conditions. The crystal structure of GFP [PDB (Protein Data Bank) ID: 2Y0G] was used and integrated with the designed LD-anchoring half-motifs in silico.

### Umbrella sampling simulation for hinge-region analysis

The free energy profile (potential of mean force) for the two designed LD-anchoring half-motifs with and without the hinge region was calculated using the umbrella sampling method (*68*), one of the enhanced sampling techniques. The initial structures of membrane-embedded half-motifs were prepared as follows: (i) Each motif was positioned with vertical alignment inside a small hole generated in the upper phospholipid layer, (ii) the center of mass (COM) of the phospholipids was restrained to maintain structural alignment, and (iii) the conformation of motifs was preserved in their original equilibrated helix structure with backbone root mean square deviation constraints. The combined structure was first equilibrated for 10 ns under the canonical (NVT) ensemble, with restraints applied to the water to prevent penetration into the phospholipid layer during the anomalous helix alignment. Subsequently, the water restraints were removed, and the simulation was extended for 10 ns. Following equilibration, the motif was positioned at 31 distinct locations relative to the phospholipid layer to initiate umbrella sampling. During the initialization phase, harmonic positional restraints with a force constant of 2000 kJ mol^−1^ nm^−2^ were applied to the COM of the motif along the *z* axis at intervals of 0.1 nm from phospholipid head layer (*z* = 0 nm) to the LD core region (*z* = 3 nm), resulting in 31 windows. Once the motif reached the target *z* position in a given window, all restraints were removed except for a reduced positional bias (700 kJ mol^−1^ nm^−2^) to maintain the motif COM at the designated *z* position. In each window, a 110-ns NVT simulation was performed, and the final 100-ns simulation of each trajectory was used to construct the potential of mean force profile via the weighted histogram analysis method (*69*). The statistical uncertainty was estimated as the standard error of the mean using five-block averaging.

### Transfer free energy calculation

The solvation free energies of IPA imine and IPA imine dimer were computed using the traditional thermodynamic integration scheme in two solvent environments, water and TAG. In these calculations, electrostatic interactions were first gradually scaled down from full strength (λ = 1) to none (λ = 0) in an interval of 0.1, followed by the scaling of Lennard-Jones interactions using a softcore potential with the same interval. The substrates were modeled as neutral species in both water and TAG. Therefore, to account for the deprotonation state of IPA imine in the cytosol environment, a correction of 7 kcal/mol was applied on the basis of the cytosolic pH of 7.2 and the p*K*_a_ (where *K*_a_ is the acid dissociation constant) of 2.1 for IPA imine (*70*).

### Plasmids and strains construction

All plasmids and strains used in this study are listed in tables S16 to S18. All modified yeast strains were derived from CEN.PK2-1D of *Saccharomyces cerevisiae* (*MAT*α *ura3-52 trp1*-*289 leu2*-*3,112 his3*Δ*1 MAL2-8C SUC2*). All plasmids were generated via insertion of a gene fragment, which had been obtained either from yeast genomic DNA or stock plasmids, amplified with corresponding primer pairs, and digested with restriction enzymes into pRS416-derived vectors under GPD1, TEF1, and CCW12 promoters for gene overexpression. For constructing LD-anchoring fluorescent protein variants in yeast, the plasmid pGADT7-ADH700-yeCherry-pTEF1(ashbya)-yeGFP-DHFR (plasmid no. 24585) for GFP and mCherry and 1x_a1gRNAOp_pCYC1m_yeBFP2 for BFP (plasmid no. 64390) were obtained from Addgene. For PDV production in yeast, the plasmid pJAS1052 (plasmid no. 158598) for VioA and VioB and pJAS1669 for VioE (plasmid no. 158594) were obtained from Addgene. In addition, lycopene-producing strains were generated by introducing pLM494 (Addgene no. 100539) containing *crtE*, *crtB*, and *crtI*. The standard LiAc/ss carrier DNA/polyethylene glycol method was used for yeast transformation (*71*). Detailed amino acid sequences for the LD-anchoring motifs are provided in table S4.

### Confocal fluorescence microscopy

For localization analysis of the fluorescent proteins fused to LD-anchoring motifs, yeast strains were grown in the yeast synthetic complete (YSC) medium (0.19% yeast synthetic dropout medium without uracil and 0.67% yeast nitrogen base without amino acids) supplemented with 2% (w/v) glucose. After growing for 24, 48, or 72 hours at 30°C with shaking at 250 rpm, the cells were fixed for 20 min with 3.7% formaldehyde in phosphate-buffered saline (pH 7.4). Then, the cells were washed twice with 1× phosphate-buffered saline and observed with a Zeiss-LSM 980 multiphoton confocal microscope (Zeiss, Germany) equipped with a Plan-Apochromat 63×/1.4–numerical aperture oil immersion objective under the Airyscan superresolution mode. Confocal images were processed and analyzed using ImageJ software (National Institutes of Health, US) and ZEN imaging software (Zeiss, Germany). Fluorescence intensities of BFP, GFP, and mCherry were quantified using ImageJ software. Relative fluorescence intensities were calculated by normalizing the fluorescence intensity of each LD-localized protein to that of its corresponding cytosolic control.

### Protein degradation assay via Western blotting

To determine the orientation of LD-anchoring GFP variants at the LD membrane surface, a protein degradation assay using proteinase K was performed on whole-cell lysates, as previously described (*72*–*74*). Briefly, yeast cells were first grown overnight at 30°C with shaking at 250 rpm in 10 ml of YSC medium supplemented with 2% (w/v) glucose. The seed cultures were then inoculated into a 250-ml bioreactor containing 50 ml of medium at an initial optical density at 600 nm (OD_600_) of 1.0 and cultivated for 48 hours. Cells were harvested and resuspended in a lysis buffer containing 50 mM tris (pH 7.4), 0.5 M NaCl, 1 mM EDTA, and 10% glycerol (*75*). The mixture was mechanically disrupted using a FastPrep-24 5G homogenizer (MP Biomedicals, US) under mild conditions to maintain LD integrity. After cell lysis, the lysate was centrifuged at 13,000 rpm for 5 min to remove cellular debris, and the resulting supernatant was collected for subsequent analysis. Then, proteinase K (40 μg/ml) was added to the supernatants and incubated at 37°C for 30 min. The reaction was stopped by the addition of phenylmethylsulfonyl fluoride (5 mM), and proteins were precipitated with 20% trichloroacetic acid. The resulting pellet was dissolved in SDS-containing Laemmli sample buffer, heated to 95°C for 15 min, and then analyzed using Western blotting.

### Western blot analysis

Proteins were separated by 10% SDS–polyacrylamide gel and transferred to a polyvinylidene fluoride membrane. Membranes were blocked with tris-buffered saline (TBS) containing 1% skim milk for 1 hour at room temperature and then incubated with mouse anti-GFP (1:1000, Invitrogen), mouse anti-BFP (1:1000, Abcam), mouse anti-mCherry (1:1000, Abcam), or mouse anti–glyceraldehyde-3-phosphate dehydrogenase (GAPDH) (1:1000, Abcam) antibodies in 1× TBS with 1% skim milk at 4°C overnight. Following incubation, membranes were treated with goat anti-mouse immunoglobulin G conjugated to horseradish peroxidase (1:1000; Jackson ImmunoResearch Laboratories) in 1× TBS with 1% skim milk for 2 hours at room temperature. Blots were visualized using a ChemiDoc MP imaging system (Bio-Rad).

### In vitro assay for VioABE activity

In vitro enzyme activity assays were conducted as previously described (*42*, *76*, *77*). Enzyme activity assays for VioABE were performed in a total volume of 5 ml containing 75 mM 4-(2-hydroxyethyl)-1-piperazineethanesulfonic acid (pH 7.5 to 8.0), 1 μM flavin adenine dinucleotide, 40 μM ammonium iron(II) sulfate, 250 μM l-tryptophan, and 100 μg of cell lysate protein. At multiple time points, 200 μl of the reaction mixture was quenched with 40 μl of 50% trichloroacetic acid and centrifuged. The supernatant was filtered through 0.2-μm syringe filters and analyzed by high-performance liquid chromatography (HPLC) using a Zorbax Eclipse XDB-C18 column (Agilent Technologies, US) at 30°C to quantify the PDV concentration. The reaction velocity was determined by plotting product formation over time, with the initial linear phase used to calculate *V*_max_. All assays were conducted in technical triplicate. To compare overall PDV formation, HPLC peak areas were analyzed at multiple time points during the reaction, with the data from the 10-min time point used, as it corresponded to the highest observed PDV production. Peak areas were used to evaluate the relative intensity of PDV produced by each strain under identical conditions.

### PDV and lycopene production

Yeast cells were grown overnight in 3 ml of YSC medium supplemented with 2% (w/v) glucose at 30°C with 250-rpm shaking. For PDV production, the seed cultures were inoculated into 10 ml of YSC medium supplemented with 2% (w/v) glucose, tryptophan (608 mg/liter), and 40 μM ammonium iron(II) sulfate in 50-ml bioreactor tubes (to an initial OD_600_ of 1.0) and then incubated at 30°C with shaking at 250 rpm for 144 hours. For lycopene production, the seed cultures were inoculated into 50 ml of YP medium containing 2% glucose in 250-ml flasks at an initial OD_600_ of 0.5 and cultivated under the same conditions (*10*). Cells were harvested after 144 hours of incubation. All PDV and lycopene production experiments were repeated at least three times independently.

### Metabolite extraction and quantification

PDV and lycopene titers were quantified as described previously (*45*, *46*, *78*, *79*). Briefly, yeast cells were harvested by centrifugation at 13,000*g* for 5 min. The harvested cells were resuspended in 0.5 ml of methanol for PDV extraction and 0.5 ml of acetone for lycopene extraction with lysing matrix C (MP Biomedicals, US), respectively. The mixture was mechanically disrupted using a FastPrep-24 5G homogenizer (MP Biomedicals, US) according to the manufacturer’s instructions (*45*, *80*). The resulting lysates were centrifuged at 13,000*g* for 10 min, and the supernatants were filtered using 0.2-μm syringe filters. The PDV extracted from the methanol solution was analyzed using an Agilent HPLC system equipped with an ultraviolet detector at 610 nm (*78*). The samples were separated on a Zorbax Eclipse XDB-C18 column (Agilent Technologies, US) at 30°C using a stepwise gradient elution at a flow rate of 1.0 ml/min for 15 min as the following method (solvent A is H_2_O with 0.1% acetate acid, and solvent B is methanol with 0.1% acetate acid): ramping from 100 to 20% solvent A in 0 to 5 min, maintaining 20% solvent A (80% solvent B) in 5 to 8 min, ramping from 20 to 100% solvent A in 8 to 12 min, and maintaining 100% solvent A in 12 to 15 min. PDV standards were provided by Lee’s Lab at Korea Advanced Institute of Science and Technology because commercial standards were not available (*79*). The lycopene extracted from the acetone solution was analyzed using an Agilent HPLC system equipped with a Prodigy column (5 μm, 250 by 4.6 mm; Phenomenex) and a ultraviolet detector set at 474 nm (*81*, *82*). Lycopene was eluted with a flow of 1.0 ml/min using the following linear gradient: starting condition, 82% A and 18% B; at 20 min, 76% A and 24% B; at 30 min, 58% A and 42% B; at 40 min, 40% A and 60% B; at 45 min, 82% A and 18% B. Phase A was a mixture of acetonitrile, *n*-hexane, methanol, and dichloromethane (2:1:1:1, v/v), and phase B was acetonitrile. The lycopene standard was purchased from Sigma-Aldrich (SMB00706).

## Supplementary Materials

### The PDF file includes:

Note S1 Methods S1 and S2 Figs. S1 to S15 Tables S1 to S18 Legends for movies S1 and S2 References
