# Supplementary material for: Spatial control of enzyme orientation at the lipid droplet interface enables regio- and chemoselective metabolic flux
Source: Sci Adv. 2026 Nov 6;12(45):eaeg5924. doi: 10.1126/sciadv.aeg5924 (PMC13742153; doi:10.1126/sciadv.aeg5924)
Supplement: Supplementary file 1 [file sciadv.aeg5924_sm.pdf]

Science Advances
AAAS

Supplementary Materials for

Spatial control of enzyme orientation at the lipid droplet interface enables regio- and chemoselective metabolic flux

So-Hee Son *et al.*

Corresponding author: Seung Soo Oh, seungsoo@postech.ac.kr; Chang Yun Son, changyunson@snu.ac.kr; Ju Young Lee, juyounglee@kaist.ac.kr

Sci. Adv. **12**, eaeg5924 (2026)
DOI: 10.1126/sciadv.aeg5924

The PDF file includes:

Note S1
Methods S1 and S2
Figs. S1 to S15
Tables S1 to S18
Legends for movies S1 and S2
References

Other Supplementary Material for this manuscript includes the following:

Movies S1 and S2

Note S1. Comparison of our directional and spatial enzyme organization strategy using designed LD-anchoring motifs with previous pathway compartmentalization strategies: Focus on violacein biosynthesis

Spatial localization of metabolic enzymes has emerged as a powerful approach for rewiring biosynthetic pathways and enhancing metabolic flux by promoting metabolic channeling and minimizing undesired side reactions. Several previous studies have explored strategies for enzyme co-localization to compartmentalize biosynthetic pathways in both prokaryotic and eukaryotic systems.

In prokaryotes, which lack membrane-bound organelles, engineered membraneless compartments have been employed to confer spatial control. For example, in the study "Encapsulation of select violacein pathway enzymes in the 1,2-propanediol utilization bacterial microcompartment to divert pathway flux (*52*)", selected enzymes from the violacein pathway were encapsulated within bacterial microcompartments (BMCs) in a cell-free context. These proteinaceous shells enabled spatial co-localization of pathway enzymes, allowing controlled encapsulation ratios and thereby facilitating metabolic channeling and diversion of pathway flux. In parallel, "Light-based control of metabolic flux through assembly of synthetic organelles (*45*)" employed optogenetic tools to trigger assembly and disassembly of metabolically active enzyme clusters. When applied to the deoxyviolacein pathway, this approach led to a 6-fold increase in product titer and an 18-fold increase in product specificity, attributed to enhanced channeling and restricted flux through competing branches. In eukaryotic systems, LD surfaces have also been explored as scaffolding platforms. The study "Synthetic Protein Scaffolds for Biosynthetic Pathway Colocalization on Lipid Droplet Membranes (*76*)" demonstrated the use of plant-derived oleosin proteins and cohesin–dockerin interaction modules to recruit multiple enzymes to LD membranes, thereby enhancing production through increased enzyme proximity.

While these studies highlight the value of spatial enzyme localization, our approach builds upon and expands these concepts by introducing a directionally programmable LD interface platform that enables precise control over enzyme orientation—either toward the cytosol or the LD core. We developed a rationally designed set of LD-anchoring motifs that enable precise orientation of cascade enzymes toward either the cytosolic space or the hydrophobic LD core, depending on the physicochemical properties of their substrates and products. This directional programmability allows for regio- and chemo-selective assembly of enzyme cascades at the amphiphilic interface of LDs, supporting more efficient and organized biosynthesis.

To our knowledge, this is the first demonstration of directionally controlled enzyme localization at the interface of an amphiphilic organelle in a living system. Our strategy not only provides a modular and tunable framework for metabolic pathway compartmentalization but also complements existing scaffolding and compartmentalization approaches by introducing an additional axis of sub-organelle-level programmability. This enables advanced spatial engineering for enhanced metabolic flux and pathway specificity in living yeast cells.

Method S1. Time-lapse fluorescence imaging of proteinase K-treated yeast spheroplasts expressing GFP fused to the designed LD-anchoring motifs

For proteinase K treatment and time-lapse imaging, yeast spheroplasts were prepared according to previously reported protocols (*83-87*). In short, yeast cells were first grown overnight at 30 °C in YSC medium supplemented with 2% (w/v) glucose with shaking at 250 rpm. The cells were harvested, washed twice with PBS, and resuspended in spheroplasting buffer (1.2 M sorbitol, 20 mM HEPES, 150 mM NaCl, pH 7.4). The resuspended cells were incubated with 50 U/mL lyticase (L4025, Sigma, USA) at 37 °C for 60 min with gentle shaking. After enzymatic digestion, the buffer was removed, and the cells were incubated with 0.001% digitonin on ice for 3 min to mildly permeabilize the plasma membrane while maintaining organelle integrity and allowing access of proteinase K. Cells were then rinsed with 5 mL of 0.6 M sorbitol buffer (identical in composition to the spheroplasting buffer, except for the reduced sorbitol concentration) and pelleted at 450 × g for 2 min. The harvested cells were resuspended in 0.3 mL of 0.6 M sorbitol buffer to an OD_{600} of 0.3. Finally, the spheroplasts were fixed on a µ-slide using poly-L-Lysine (ibidi, USA) and treated with proteinase K (100 µg/mL) in 0.6 M sorbitol buffer with 0.01% digitonin. Time-lapse fluorescence images were acquired every 5 sec using a Zeiss LSM 980 multiphoton confocal microscope (Zeiss, Germany) equipped with a Plan-Apochromat 63x/1.4 NA oil immersion objective.

Method S2. In vitro biosynthesis assays for VioE and CrtE

To enable accurate comparison of enzyme activities across different strains, all VioE and CrtE proteins were fused with a C-terminal histidine (6×His) tag. His-tagged proteins were purified using the Capturem™ His-Tagged Purification Kit (Takara Bio, Japan), and protein concentrations were quantified using the Pierce™ Rapid Gold BCA Protein Assay Kit (Thermo Fisher Scientific, USA) prior to the assay. Biosynthetic outputs were normalized based on the abundance of purified protein obtained from each construct, and the resulting product formation intensities were further normalized to those of the corresponding cytosolic VioE and CrtE controls for relative activity comparison.

For VioE assays, the pathway intermediate IPA imine dimer was supplied using yeast lysates expressing the upstream enzymes VioA and VioB, because this intermediate is not commercially available. Equal amounts of total protein from VioAB-expressing lysates (500 µg), based on previous crude lysate-based pathway reconstitution studies (*88, 89*), were used to provide a freshly generated pathway intermediate for all reactions. In vitro biosynthetic assays were performed in a total volume of 5 mL containing 500 µg of total protein from VioAB-expressing lysates, 75 mM 4-(2-hydroxyethyl)-1-piperazineethanesulfonic acid (HEPES; pH 7.5–8.0), 1 µM flavin adenine dinucleotide, 40 µM ammonium iron (II) sulfate, 250 µM L-tryptophan, and 100 µg of normalized VioE protein. Based on preliminary time-course experiments showing that the reaction proceeded within the initial linear phase during the first 10 min, 200 µL of the reaction mixture was collected after incubation at 37 °C for 10 min, quenched with 40 µL of 50% TCA, and centrifuged. The supernatant was filtered through 0.2 µm syringe filters and analyzed by HPLC using a Zorbax Eclipse XDB-C18 column (Agilent Technologies, USA) at 30 °C to quantify PDV concentration.

For CrtE assays, reactions were performed in a total volume of 5 mL containing 75 mM HEPES buffer (pH 7.5), 50 µM farnesyl pyrophosphate (FPP), 250 µM isopentenyl pyrophosphate (IPP), 5 mM $MgCl_2$, and 100 µg of normalized CrtE protein. The reaction was incubated at 37 °C for 120 min. At multiple time points, 200 µL of the reaction mixture was quenched with 40 µL of 50% TCA and centrifuged. The geranylgeranyl pyrophosphate (GGPP) obtained from the reaction mixture was filtered through 0.2 µm syringe filters. The GGPP intensity over time was used to determine reaction velocity, and the initial linear phase was used to calculate the maximum velocity (V_{max}). To analyze GGPP, an Agilent HPLC system equipped with a Zorbax SB-C18 column (4.6 × 150 mm, 5 µm; Agilent Technologies, USA) was used. The HPLC method was modified from previously reported methods for GGPP analysis (*90*). Samples were separated at a flow rate of 0.8 mL/min using solvent A (5 mM ammonium bicarbonate in water) and solvent B (acetonitrile) with the following gradient elution method: 0–2 min, 0–10% solvent B; 2–14 min, 10–64% solvent B; 14–16 min, 64–100% solvent B; and 16–17 min, return to 0% solvent B. GGPP standards were purchased from Sigma Aldrich (G6025).

To verify enzyme orientation, cell lysates were treated with 0.5 mg/mL Proteinase K, a concentration selected based on previous protease protection and membrane topology assays (*91, 92*), at 37 °C for 10 min prior to the assay. Subsequent in vitro biosynthesis assays were then performed and quantified using the same procedures described above.

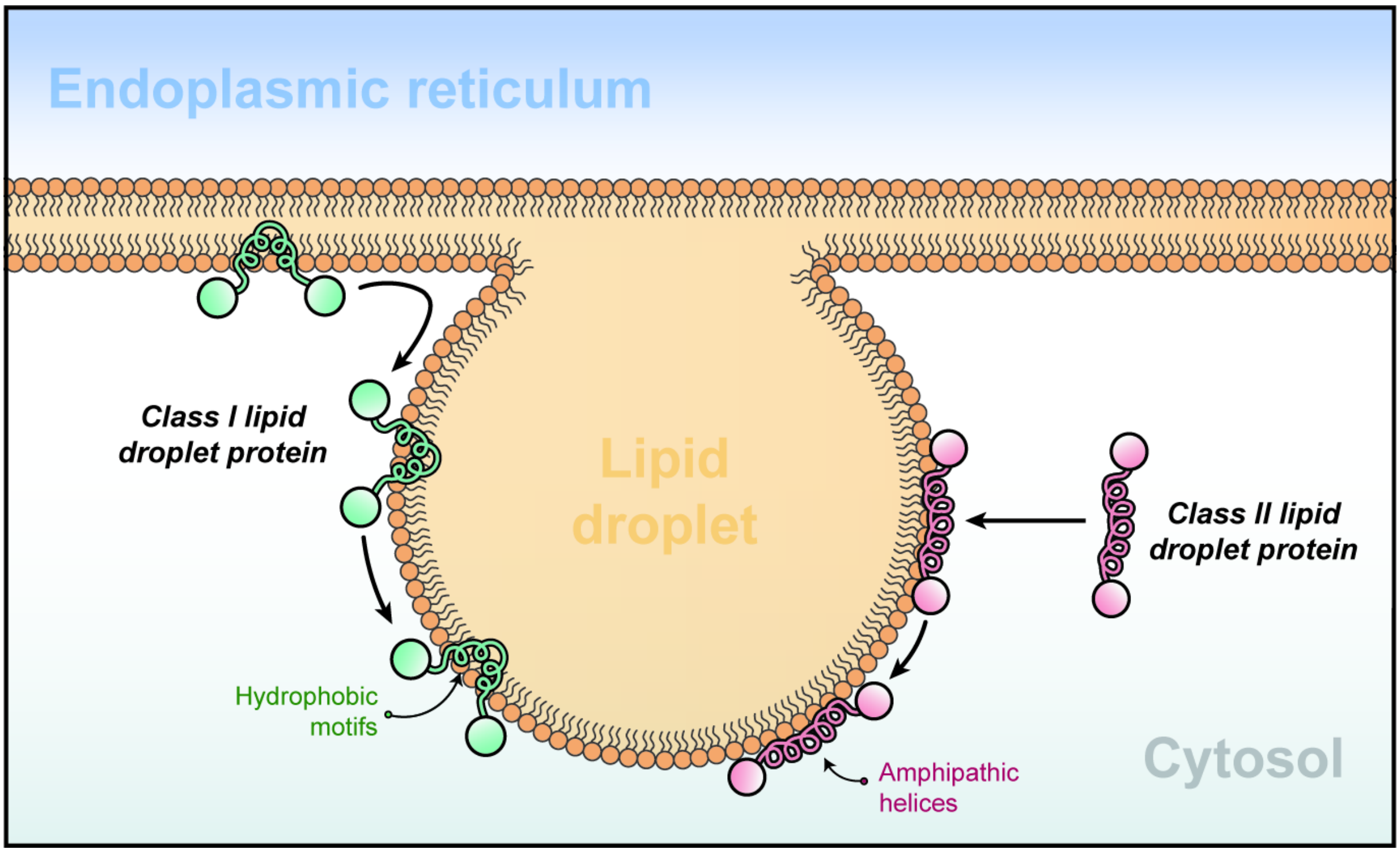

Fig. S1. Targeting mechanisms of Class I and Class II lipid droplet (LD) proteins. LD proteins are targeted to the LD surface through distinct mechanisms from either the endoplasmic reticulum (ER) membrane or the cytosol. Class I LD proteins are initially embedded in the ER membrane via hydrophobic motifs, and subsequently relocate onto the LD phospholipid monolayer during LD formation. In contrast, Class II LD proteins are synthesized in the cytosol and directly associate with the LD surface by inserting amphipathic helices into the monolayer.

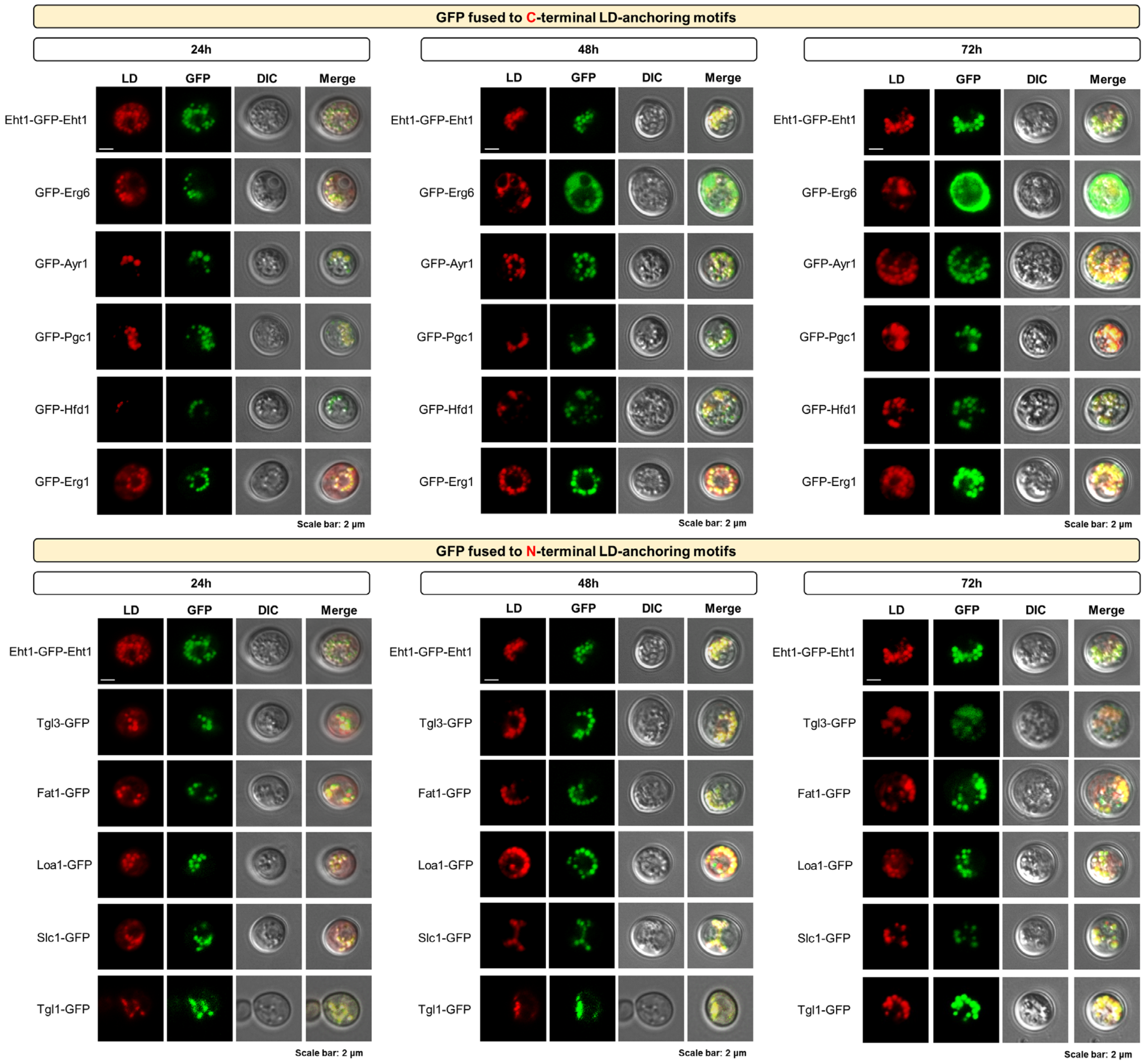

Fig. S2. Localization of Green Fluorescent Protein (GFP) fused to LD-anchoring motifs in wild-type yeast cells. The fluorescence of GFP fused to LD-anchoring motifs was observed at 24, 48, and 72 hours. The top section shows the localization of GFP fused to C-terminal LD-anchoring motifs; the bottom section presents GFP fused to N-terminal LD-anchoring motifs. Yeast cells were stained with Nile Red to visualize LDs (red), and GFP fluorescence (green) was detected by confocal microscopy. Differential interference contrast (DIC) images and merged images (LD, GFP, and DIC) are also shown. Scale bars, 2 µm.

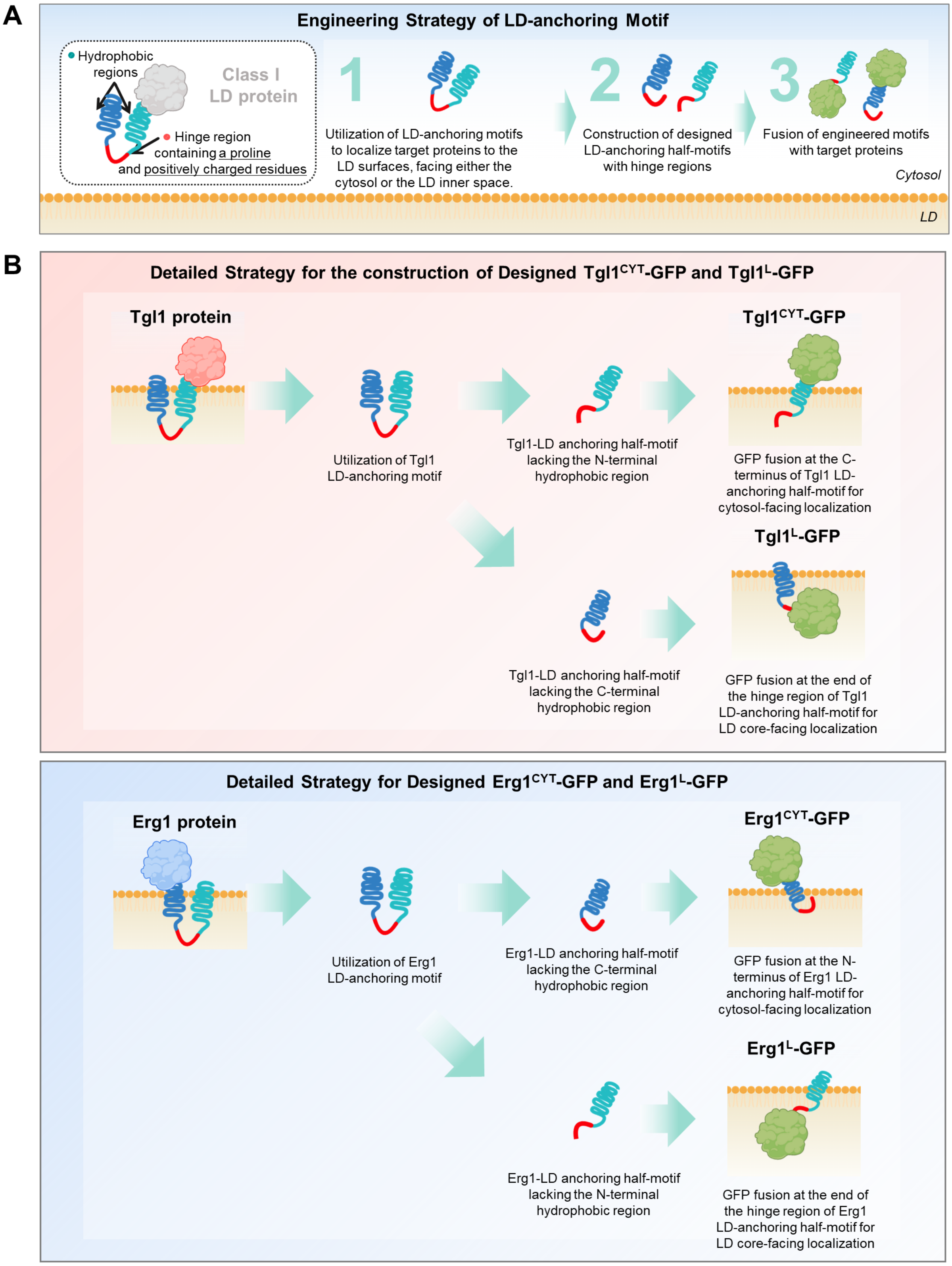

Fig. S3. Engineering strategy for constructing designed LD-anchoring motifs. (A) Schematic overview of the design strategy for LD-anchoring motifs. Class I LD proteins typically contain a membrane-embedded, V-shaped hydrophobic structure composed of two

hydrophobic regions flanking a hinge region enriched in proline and positively charged residues. To engineer LD-anchoring motifs, we first screened Class I LD proteins (Step 1), selectively deleted one hydrophobic region to generate a half-motif structure (Step 2), and subsequently fused the engineered LD-anchoring half-motifs to target proteins to enable directed localization to the LD surface (Step 3). **(B)** Detailed construction strategies for Tgl1- and Erg1 LD-anchoring motifs fused to GFP. For each motif, the native LD-anchoring motif was identified, one hydrophobic region was selectively removed, and GFP was fused either to the N-terminus or to the end of the hinge region of the half-motifs, generating two distinct orientation-controlled anchoring designs.

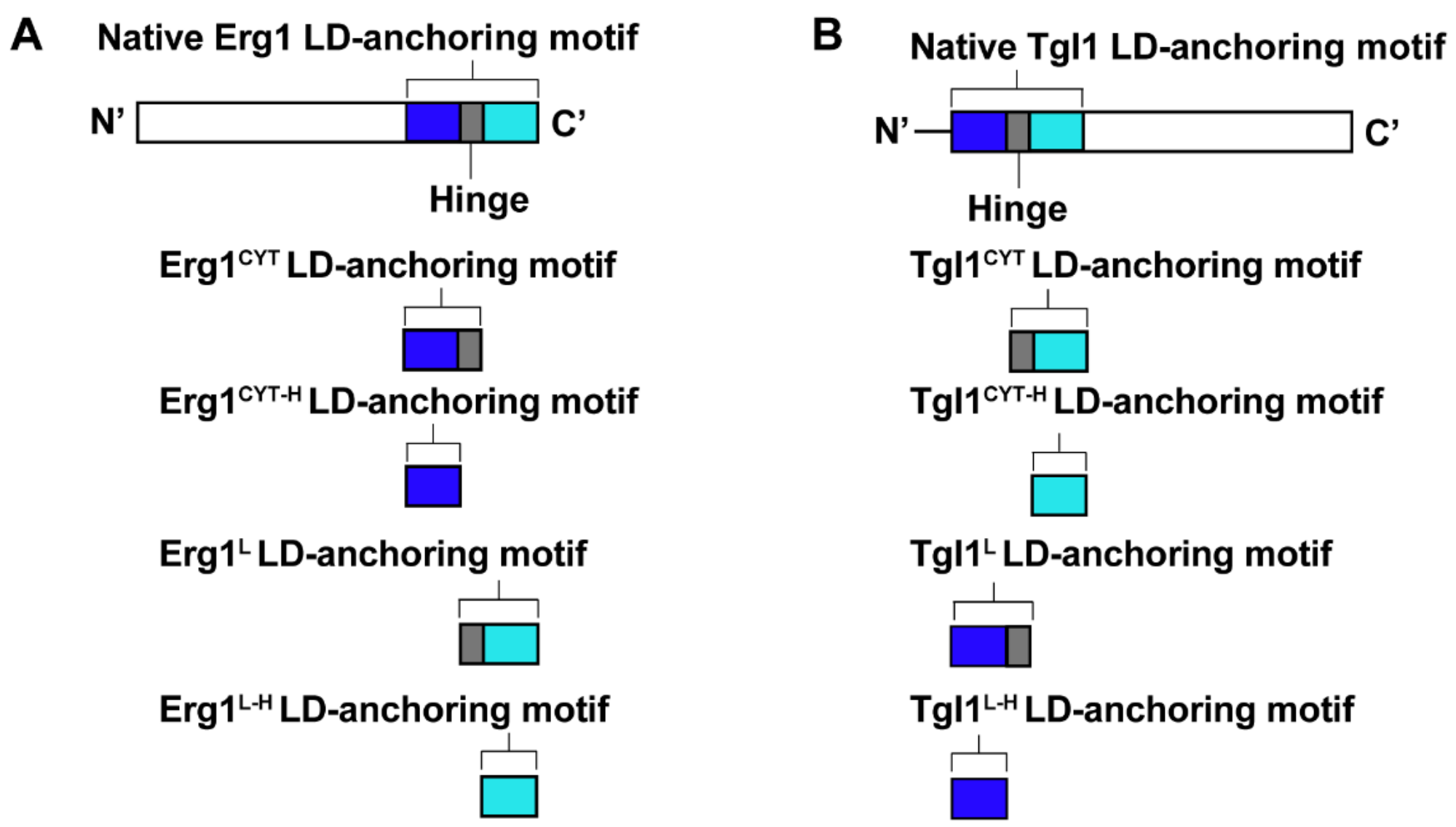

LD-anchoring motif-hinge region-LD-anchoring motif

A Erg1 amino acid sequences
MSAVNVAPELINADNTITYDAIVIGAGVIGPCVATGLARKGKKVLIVERDWAMPD
RIVGELMQPGGVRALRSLGMIQSINNIEAYPVTGYTVFFNGEQVDIPYPYKADIPKV
EKLKDLVKDGNDKVLEDSTIHIKDYEDDERERGVAFVHGRFLNNLRNITAQEPNV
TRVQGNCIEILKDEKNEVVGAKVDIDGRGKVEFKAHLTFICDGIFSRFRKELHPDH
VPTVGSSFVGMSLFNAKNPAPMHGHVILGSDHMPILVYQISPEETRILCAYNSPKVP
ADIKSWMIKDVQPFIPKSLRPSFDEAVSQGKFRAMPNSYLPARQNDVTGMCVIGD
ALNMRHPLTGGGMTVGLHDVVLLIKKIGDLDFSDREKVLDELLDYHFERKSYDSV
INVLSVALYSLFAADSDNLKALQKGCFKYFQRGGDCVNKPVEFLSGVLPKPLQLT
RVFFAVAFYTIYLNMEERGFLGLPMALLEGIMILITAIRVFTPFLFGELIG*

B Tgl1 amino acid sequences
MYFPFLGRLSITDYIIVVLVYIESIISSVLKLIPQPMINLFEWLINFSTSSDDNTIEEKL
RSAPTIHEMCAIFDISVEDHLVRTEDNYILTLHRIPPISKNRFNNKVVYLHHGLLMC
SDVWCCNIERHKNLPFVLHDLGYDVWMGNNRGNKYSTAHLNKPPKSNKFWDFSI
DEFAFFDIPNSIEFILDITKVDKVICIGFSQGSAQMFAAFSLSEKLNRKVSHFIAIAPA
MTPKGLHNRIVDTLAKSSPGFMYLFFGRKIVLPSAVIWQRTLHPTLFNLCIDIANKI
LFNWKSFNILPRQKIASYAKLYSTTSVKSIVHWFQILRSQKFQMFEESDNMLNSLTR
PYQIANFPTRTNIKIPILLIYGGIDSLVDIDVMKKNLPFNSVFDVKVDNYEHLDLIWG
KDADTLVIAKVLRFIEFFNPGNVSVKTNQLLPSASLVEELPSTTWKTTHPTHGLSYR
THSADRSPLSVQADEADEVHNADNSRFLRRVFSTSAIDEDNENEHQDDTEDQIHKE
QQRRLSAYLESSKDLRQLDANSSTTALDALNKE*

Fig. S4. Schematic representation and amino acid sequences of native and designed LD-anchoring motifs derived from Erg1 and Tgl1. (A, B) Native LD-anchoring motifs from Erg1 and Tgl1 proteins are shown, along with their engineered, truncated versions used in

this study. Positively charged (red) and proline (orange)-enriched residues within the hinge regions are highlighted.

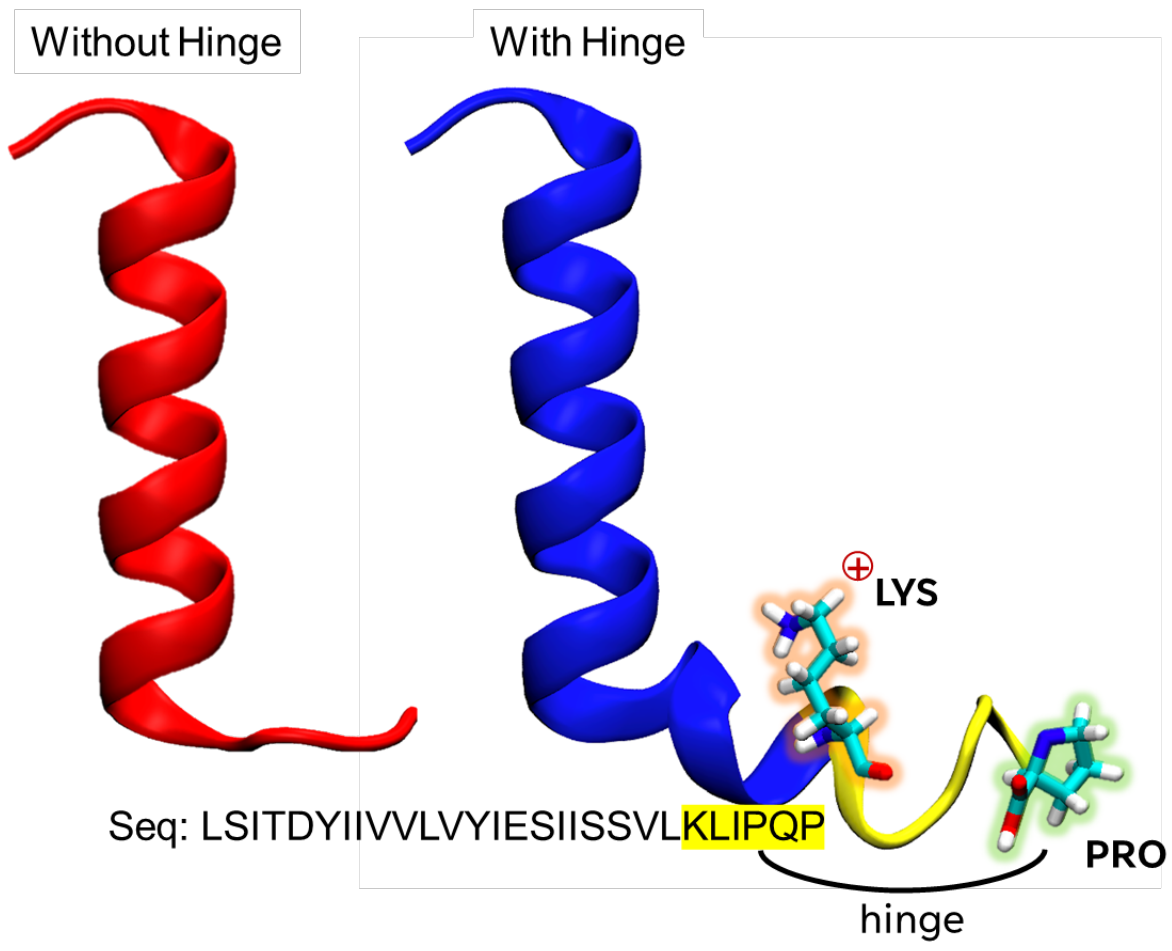

Fig. S5. AlphaFold2-predicted structures of two truncated variants of the Tgl1 LD-anchoring motifs. Both designed motifs contain the N-terminal helix domain, while only the hinge-containing designed half-motif has an additional six amino acid residues with a positively charged lysine residue and a conserved proline residue, which provide key molecular interactions to stabilize the designed half-motif at the LD surface.

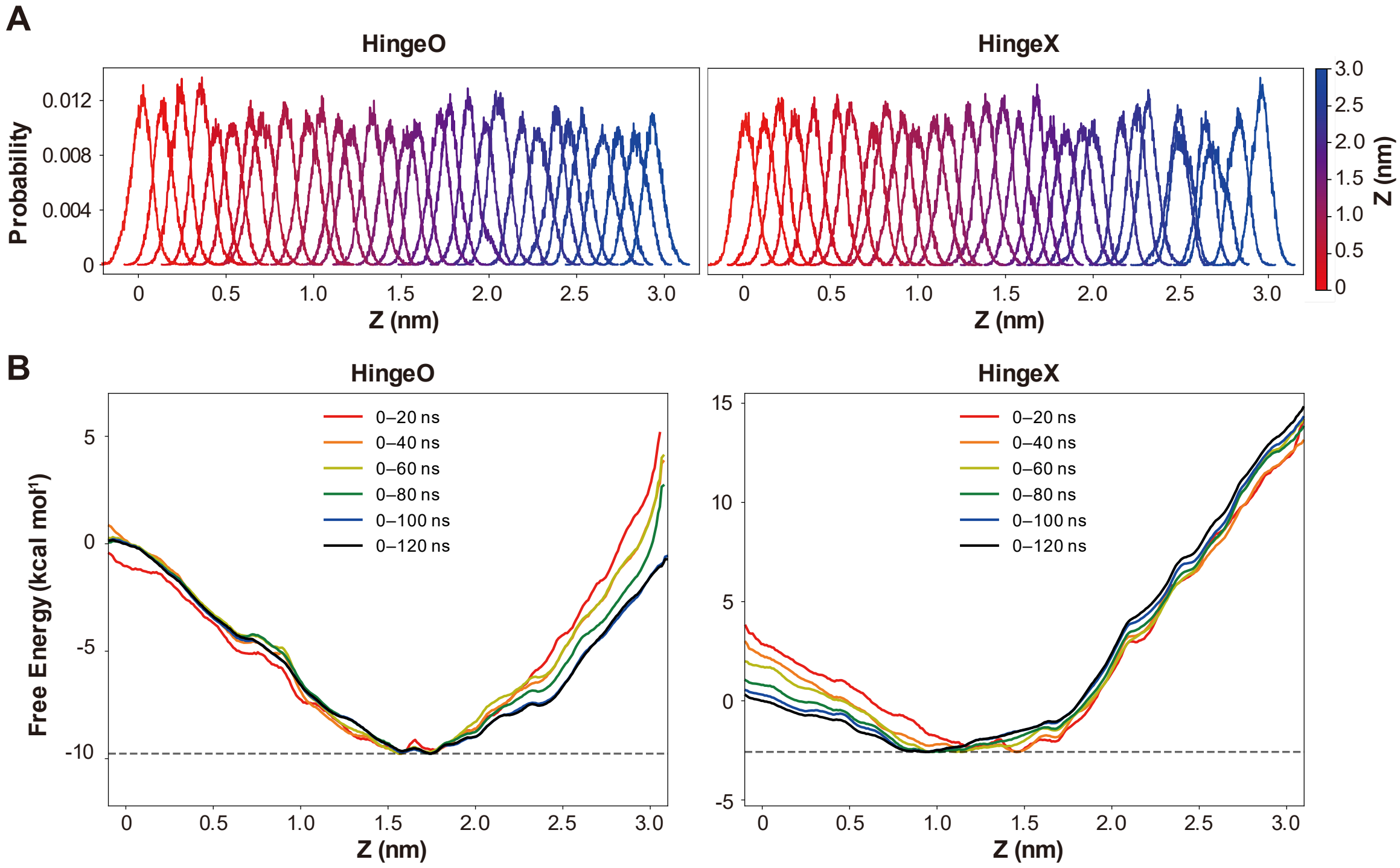

Fig. S6. Validation of umbrella sampling convergence. (A) Probability distributions of umbrella sampling windows along the Z-coordinate for the Hinge O and Hinge X systems. **(B)** PMF convergence tests for the Hinge O and Hinge X systems.

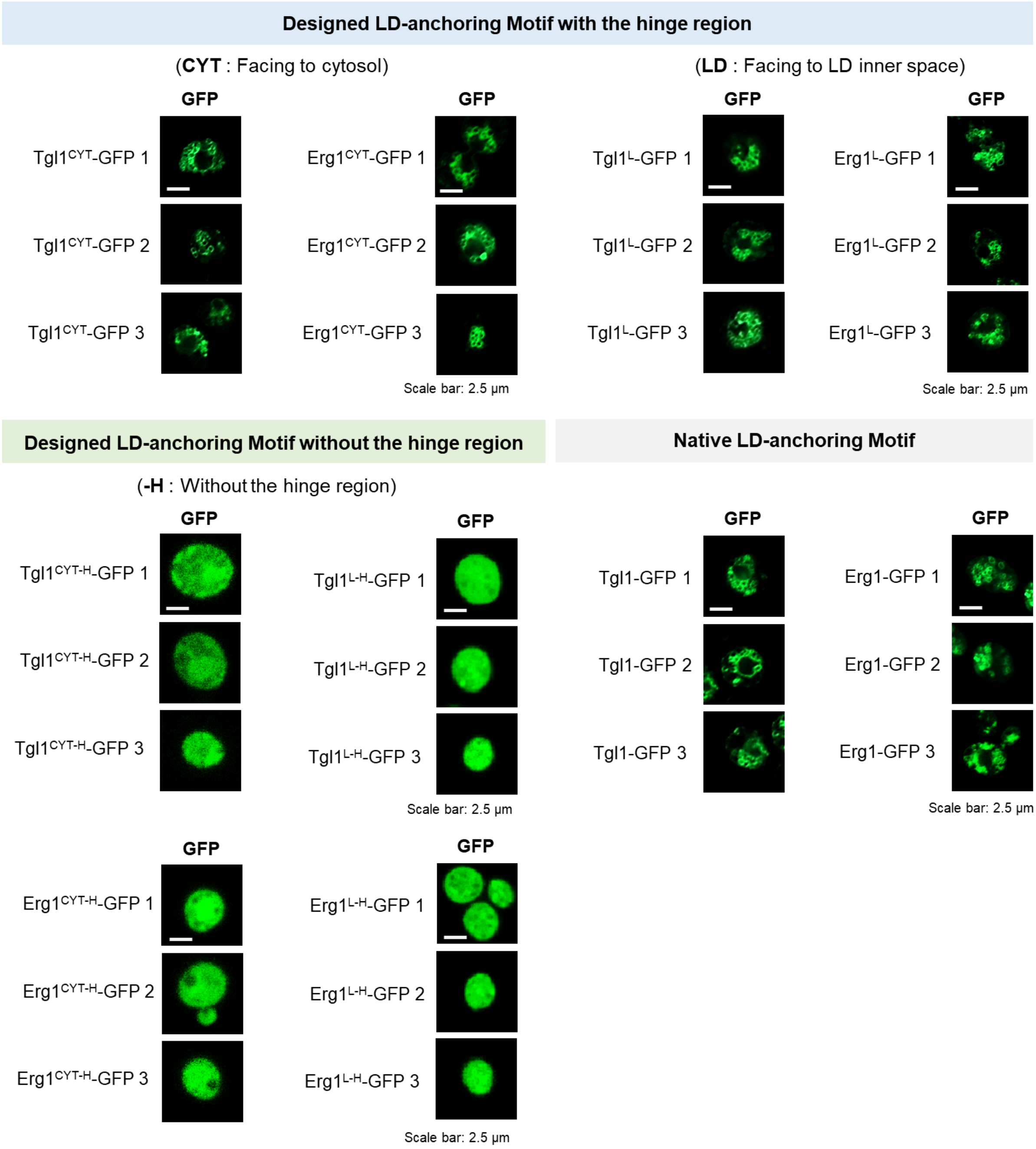

Fig. S7. Localization of GFP fused to designed LD-anchoring motifs in wild-type yeast cells. The localization of GFPs fused to designed LD-anchoring motifs with or without the hinge region, and native LD-anchoring motifs was analyzed by confocal microscopy. The top section shows GFP fused to designed LD-anchoring motifs with the hinge region: constructs facing the cytosol (Tgl1^{CYT}-GFP and Erg1^{CYT}-GFP) and constructs facing the LD inner space (Tgl1^{L}-GFP and Erg1^{L}-GFP). The bottom left section presents GFP fused to hinge region-lacking designed LD motifs (Tgl1^{CYT-H}-GFP, Tgl1^{L-H}-GFP, Erg1^{CYT-H}-GFP, and Erg1^{L-H}-GFP). The bottom right section shows GFP fused to the native LD-anchoring motifs (Tgl1-GFP and Erg1-GFP). Scale bars, 2.5 μm.

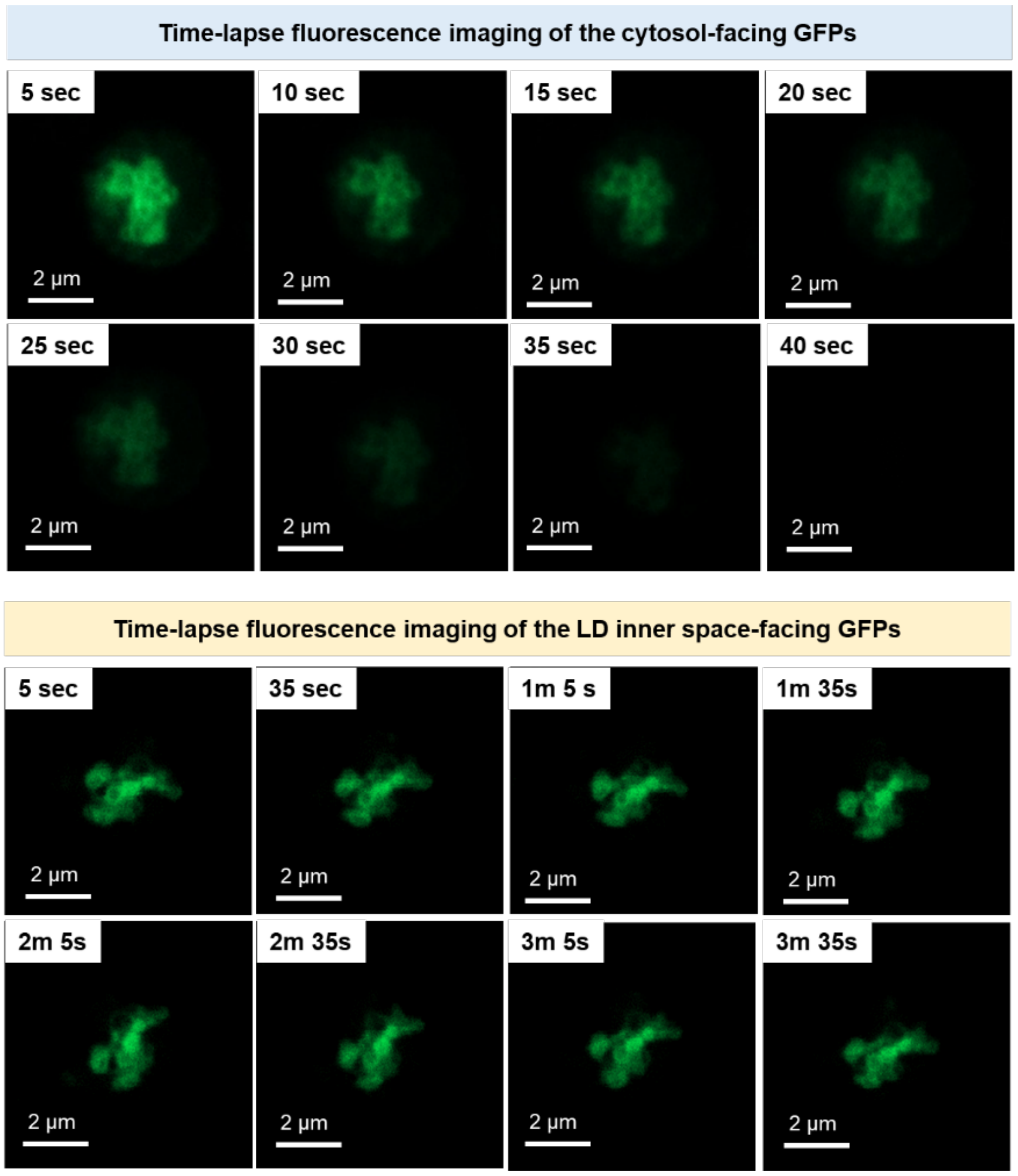

Fig. S8. Time-lapse fluorescence microscopy images showing rapid loss of fluorescence of the cytosol-facing GFP (top) and stable fluorescence of the LD inner-facing GFP (bottom). Digitonin-permeabilized yeast spheroplasts expressing either the cytosol-facing GFP (Tgl1^{CYT}-GFP) or the LD inner space-facing GFP (Tgl1^{L}-GFP) were treated with proteinase K (see Method S1 for details). Upon proteinase K treatment, the fluorescence signal of the cytosol-facing GFPs (top) rapidly decreased, while that of the LD inner-facing GFPs (bottom) remained stable throughout the imaging (see Movies S1 and S2 for details). Time-lapse fluorescence images were acquired every 5 sec using a Zeiss LSM 980 multiphoton confocal microscope (Zeiss, Germany) equipped with a Plan-Apochromat 63x/1.4 NA oil immersion objective. Scale bar, 2 μm.

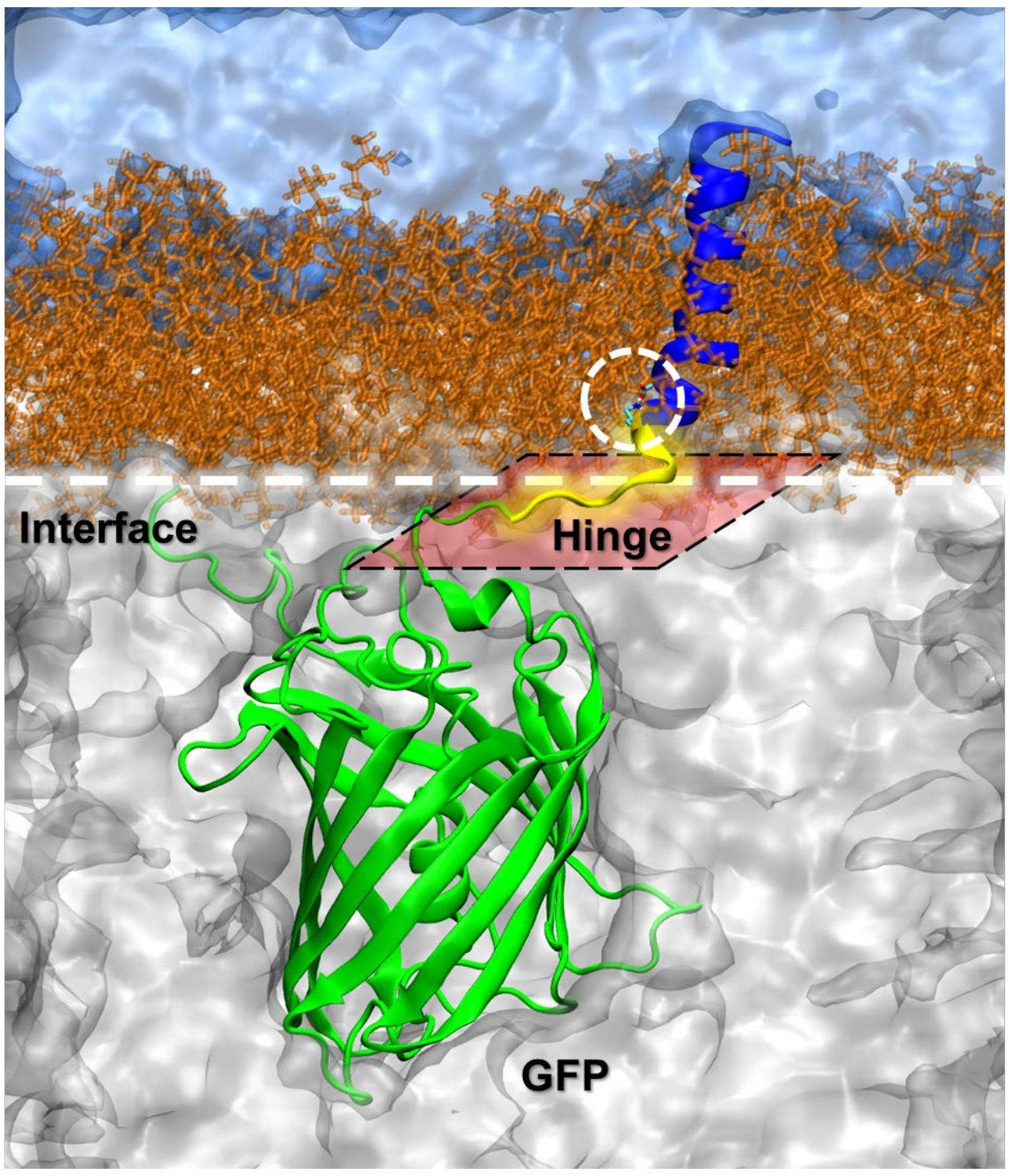

Fig. S9. Equilibrated MD simulation structure of Tgl1^{L}-GFP embedded on the LD surface retains functional GFP protein structure (colored in green) within the LD core. The hinge region (colored in yellow) and the salt-bridge interaction (white circle) in the designed Tgl1 half-motif (colored in blue-yellow) are highlighted to show their distinct role in forming an L-shaped interfacial anchoring at the phospholipid-LD core interface.

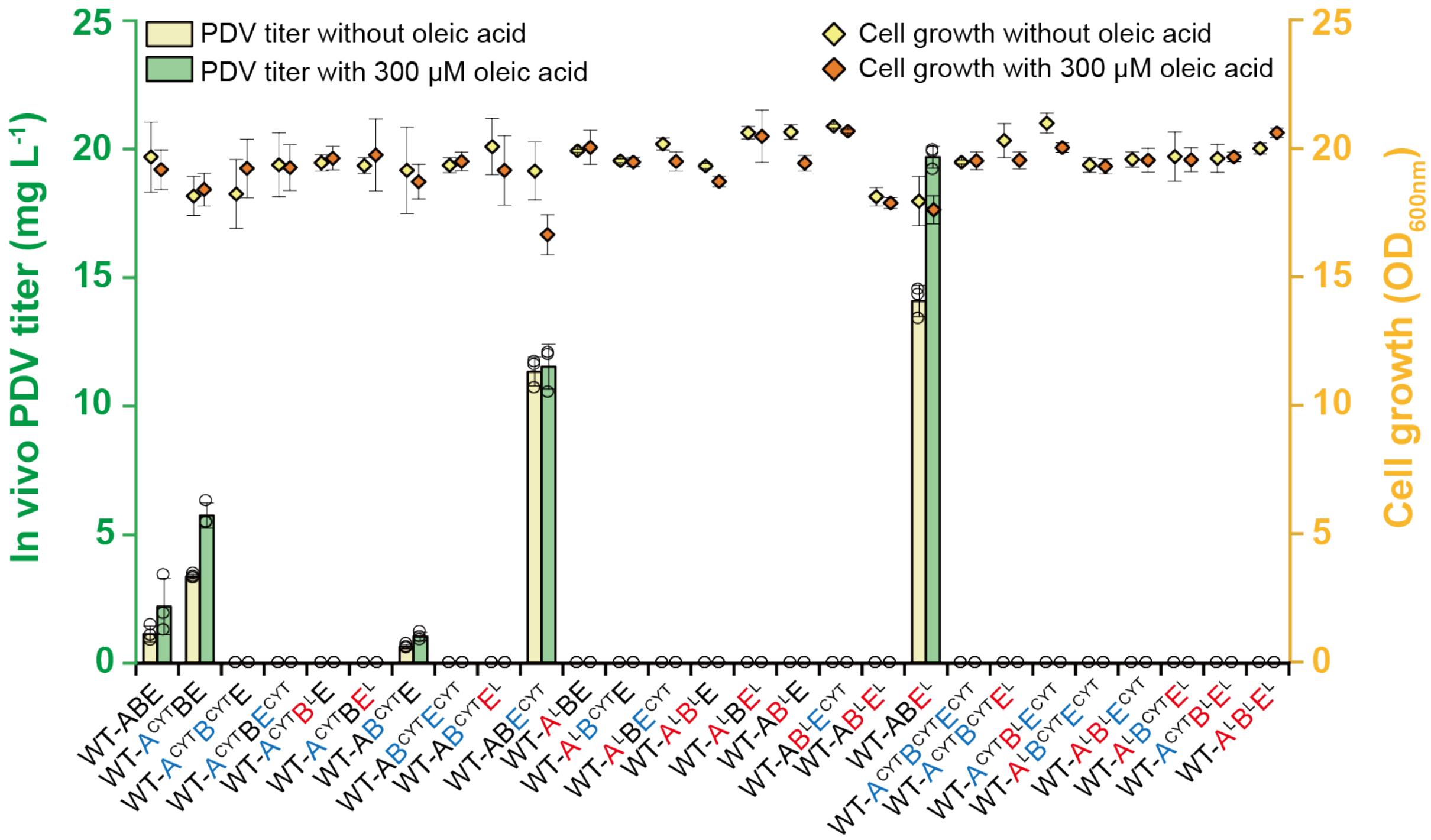

Fig. S10. In vivo PDV production and cell growth of wild-type (WT) yeast strains expressing LD-anchored PDV biosynthetic enzymes with or without oleic acid supplementation. PDV production titers and cell growth were measured in WT yeast strains expressing various combinations of LD-anchoring PDV biosynthetic enzymes (VioA, VioB, and VioE) in the presence or absence of 300 µM oleic acid supplementation. When the PDV biosynthetic enzymes were localized to LD surfaces, PDV production was more efficient in our LD-engineered yeast strains from our previous study (See Fig. 5B) compared to the WT yeast strains. Yeast strains were cultivated in 10 mL of defined minimal media containing 2% (w/v) glucose, 608 mg/L L-tryptophan, and 40 µM ammonium iron (II) sulfate, with or without 300 µM oleic acid, for 144 h at 30 °C in a 50 mL bioreactor. Yellow and green bars represent PDV titers without and with oleic acid supplementation, respectively. Yellow and orange diamonds indicate OD_{600} values without and with oleic acid supplementation, respectively. Data are shown as mean ± SD from three independent experiments (Table S6).

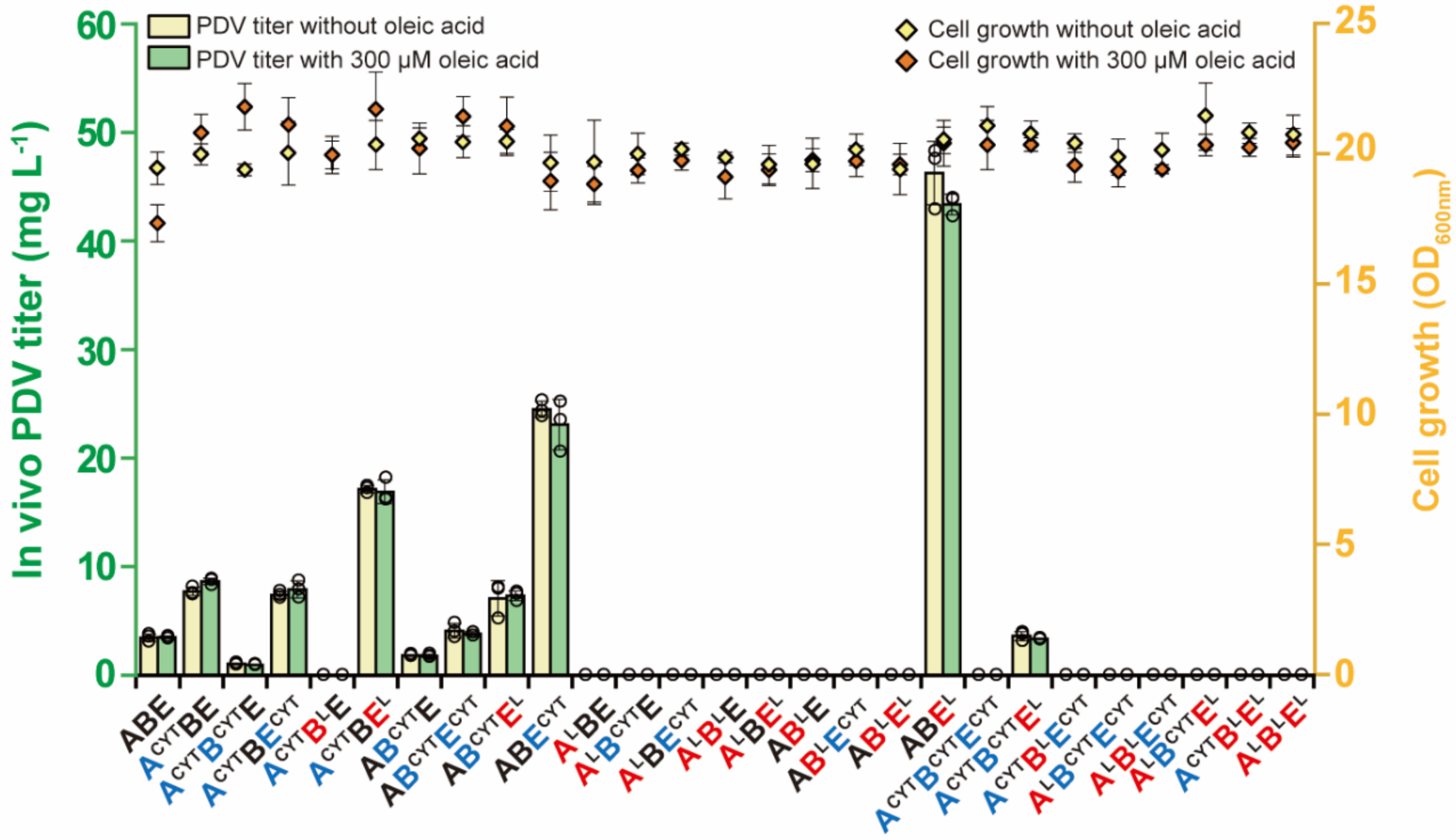

Fig. S11. Effects of oleic acid supplementation on in vivo PDV production and cell growth in LD-engineered yeast cells expressing LD-anchored PDV biosynthetic enzymes. PDV production titers and cell growth were measured in previously developed LD-engineered yeast strains (LD-size yeast cell from our previous study) (*9*) expressing various combinations of LD-anchored PDV biosynthetic enzymes (VioA, VioB, and VioE) in the presence or absence of 300 µM oleic acid supplementation. Yeast strains were cultivated in 10 mL of defined minimal medium containing 2% (w/v) glucose, 608 mg/L L-tryptophan, and 40 µM ammonium iron (II) sulfate, with or without 300 µM oleic acid, for 144 h at 30 °C in 50 mL bioreactors. Yellow and green bars represent PDV titers without and with oleic acid supplementation, respectively. Yellow and orange diamonds indicate OD_{600} values without and with oleic acid supplementation, respectively. Data are presented as mean ± SD from three independent experiments (Table S7).

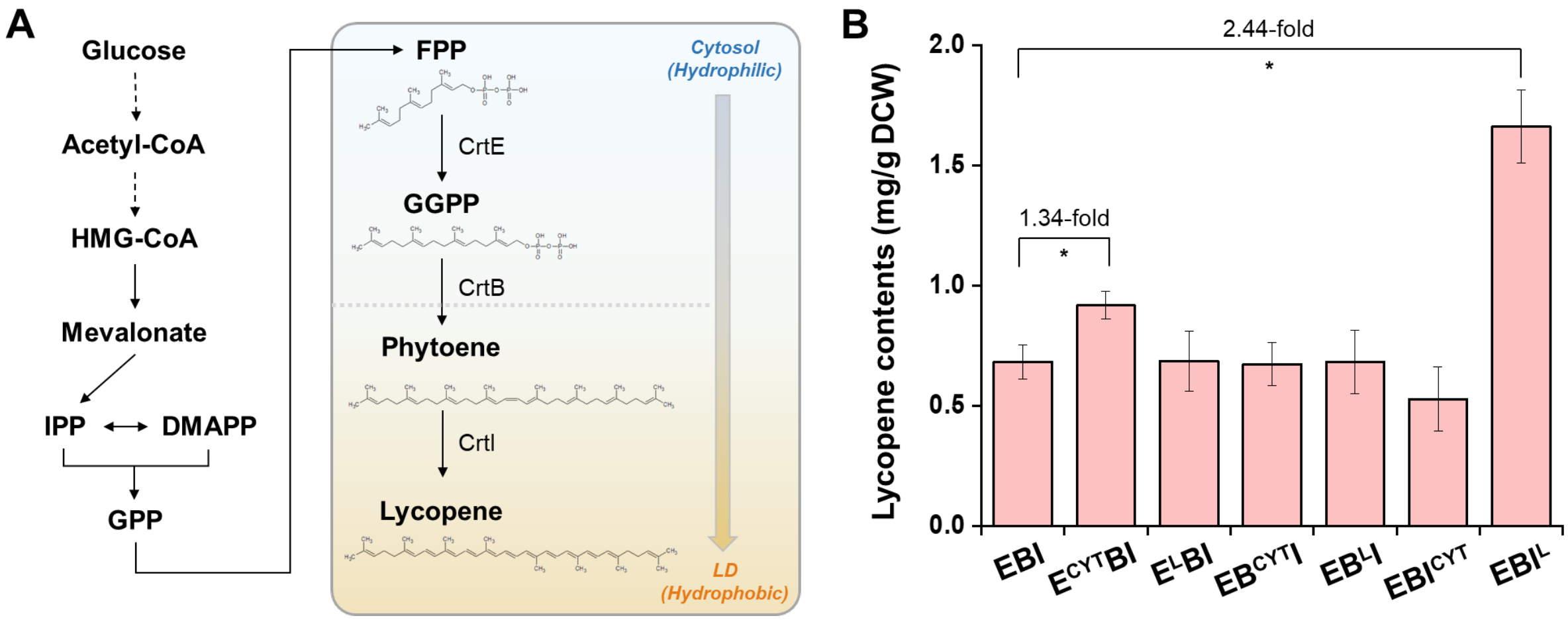

Fig. S12. In vivo lycopene contents in yeast strains expressing LD-anchoring lycopene biosynthetic enzymes. (A) The biosynthetic lycopene pathway consisting of *Xanthophyllomyces dendrorhous* CrtE, CrtB and CrtI. Farnesyl pyrophosphate (FPP) is converted to geranylgeranyl pyrophosphate (GGPP) by CrtE, followed by phytoene synthesis by CrtB and subsequent desaturation to lycopene by CrtI. The vertical gradient indicates the increasing hydrophobicity of pathway intermediates from FPP to lycopene. **(B)** Spatial organization of lycopene biosynthetic enzymes on the inner and outer surfaces of LDs using designed LD-anchoring motifs. Anchoring CrtI toward the LD inner space (EBI^{L}) resulted in a lycopene accumulation of 1.66 mg/g DCW—a 2.44-fold increase compared to the cytosolic control (EBI, expressing CrtE, CrtB, and CrtI in the cytosol). Cytosol-facing anchoring of CrtE ($E^{CYT}BI$) also increased lycopene production to 0.92 mg/g DCW (a 1.34-fold increase over the cytosolic control EBI). Yeast strains were cultivated in 50 mL of YP media containing 2% (w/v) glucose for 144 h at 30 °C in a 250 mL bioreactor. Lycopene production experiments were repeated at least three times independently (Table S11). Error bars represent standard deviations from biological replicates, and asterisks (*) indicate p-values < 0.05.

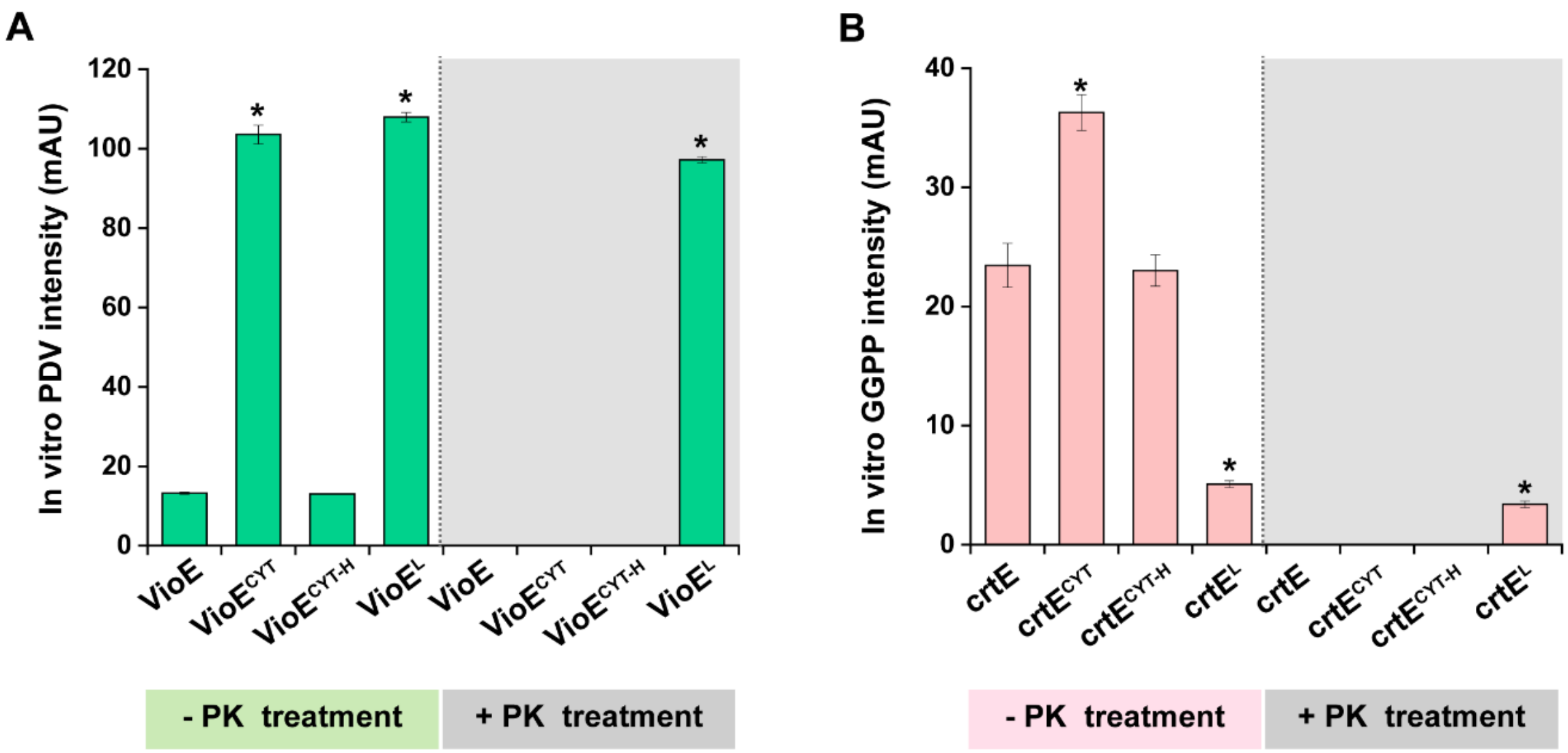

Fig. S13. Verification of enzyme orientation and biosynthetic performance of LD-anchored VioE and CrtE variants using in vitro biosynthesis assays. To evaluate enzyme orientation and biosynthetic output upon LD anchoring, in vitro biosynthesis assays were performed under Proteinase K-untreated (−PK) and Proteinase K-treated (+PK) conditions (see Methods S2 for details). All samples were collected 10 min after reaction initiation. PDV and GGPP production levels (mAU) were quantified after the reactions using normalized amounts of each target protein (VioE or CrtE). All in vitro biosynthesis experiments were repeated at least three times independently (Table S13). Error bars represent standard deviations from biological replicates. Asterisks (*) indicate statistical significance ($P < 0.05$) compared to the corresponding cytosolic VioE or CrtE control under the same Proteinase K treatment condition, as determined by a two-sided t-test. mAU, milli absorbance units. **(A)** In vitro PDV production using VioE variants. The pathway intermediate IPA imine dimer was supplied using VioAB-expressing yeast lysates, as this intermediate is not commercially available. Without Proteinase K treatment (−PK), both cytosol-facing (VioE^{CYT}) and LD inner-facing (VioE^{L}) LD-anchored VioE exhibited enhanced PDV production compared with the cytosolic control (VioE). In contrast, the hinge-deleted VioE variant (VioE$^{CYT\text{-}H}$) showed PDV production levels comparable to those of the cytosolic control, indicating that deletion of the hinge region in the designed LD-anchoring motifs impaired the stable localization of the enzyme at the LD interface. Following Proteinase K treatment (+PK), only LD inner-facing VioE (VioE^{L}) retained detectable PDV production. **(B)** In vitro GGPP production using CrtE variants. Under Proteinase K-untreated (−PK) conditions, cytosol-facing LD-anchored CrtE (CrtE^{CYT}) exhibited enhanced GGPP production compared with the cytosolic control (CrtE). In contrast, LD inner-facing CrtE (CrtE^{L}) showed only minimal GGPP production, likely reflecting severely restricted substrate accessibility, although small prenyl intermediates such as FPP may still partially access the LD interface and permit residual activity. Similarly, the hinge-deleted CrtE variant (CrtE$^{CYT\text{-}H}$) exhibited GGPP production levels comparable to those of the cytosolic control, suggesting impaired LD localization upon hinge deletion. Following Proteinase K treatment (+PK), no detectable GGPP production was observed for any CrtE variants.

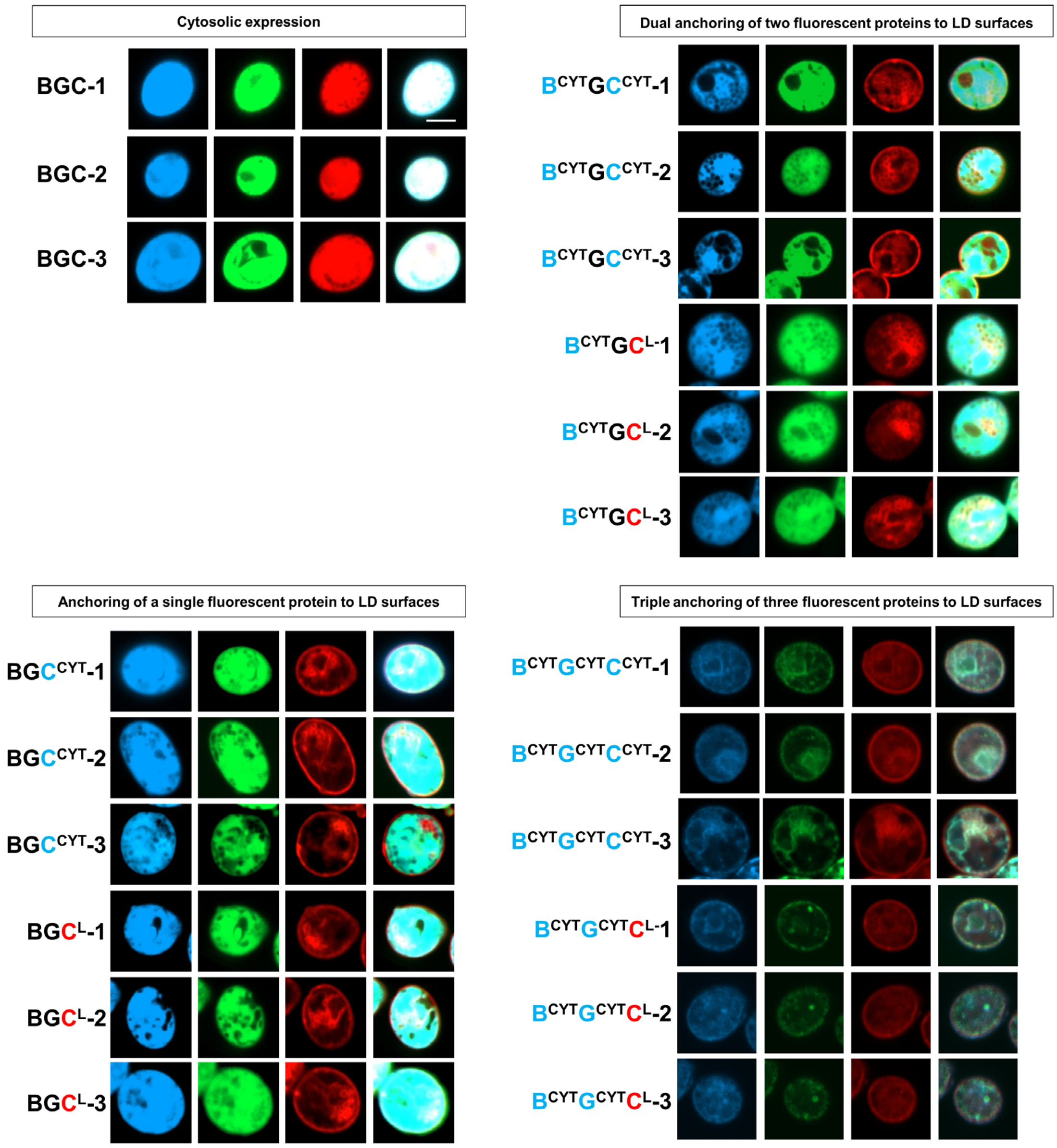

Fig. S14. Confocal microscopy analysis of fluorescent proteins anchored to LD surfaces using designed LD-anchoring motifs. Confocal microscopy images show the subcellular localization and fluorescence intensity of fluorescent proteins. Blue fluorescent protein (BFP, B, blue channel) and GFP (G, green channel) were anchored via the designed Erg1 LD-anchoring motif, while mCherry (C, red channel) was anchored using the designed Tgl1 LD-anchoring motif, following the same strategy applied for PDV biosynthetic enzymes. Fluorescence signals for BFP, GFP, and mCherry are shown individually and as merged images to illustrate co-localization patterns. (Top left) Cytosolic expression of BFP, GFP, and mCherry without LD targeting. (Bottom left) Anchoring of a single fluorescent protein to LD surfaces. (Top right) Dual anchoring of two fluorescent proteins to LD surfaces. (Bottom right) Triple anchoring of three fluorescent proteins to LD surfaces. Scale bars, 2.5 µm.

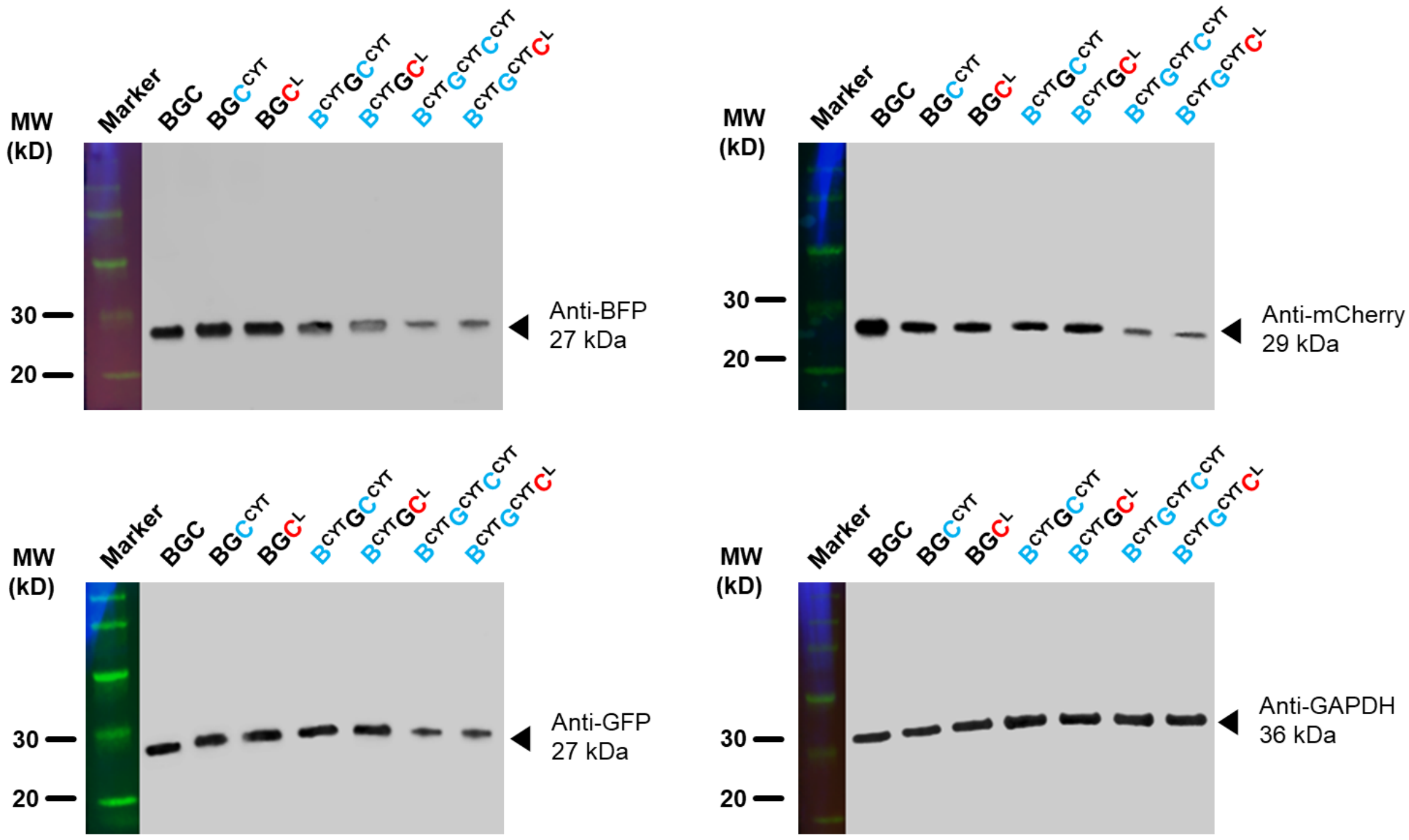

Fig. S15. Uncropped western blot images corresponding to the quantification shown in Fig. 5C. Western blot images showing BFP, mCherry, GFP, and GAPDH in the indicated strains. Western blotting was performed on separate membranes using identical samples loaded with equal amounts of total protein in the same lane order. GAPDH was used as the loading control.

Table S1. List of LD proteins identified in the *Saccharomyces cerevisiae* LD proteome with ≥ 0.3% relative abundance (*20*).

	Gene	Description	Localization	Relative abundance (%)
1	*ERG6*	Sterol 24-C-methyltransferase	ER/LD/M	5.31
2	*FAA4*	Long chain fatty acyl-CoA synthetase	LD/Cm	1.40
3	*FAT1*	Very long chain fatty acyl-CoA synthetase	ER/LD/PM/P/IntM	1.39
4	*TGL1*	Steryl ester hydrolase	LD/IntM	1.39
5	*PGC1*	Phosphatidyl glycerol phospholipase C	LD/M	1.07
6	*AYR1*	1-acyldihydroxyacetone-phosphate reductase	ER/LD/Cm/IntM/M	1.01
7	*ERG7*	Lanosterol synthase	ER/LB/PM	0.91
8	*SLC1*	1-acyl-sn-glycerol-3-phosphate acyltransferase	LD	0.84
9	*TGL3*	Bifunctional triacylglycerol lipase	LD	0.62
10	*TGL5*	Bifunctional triacylglycerol lipase	LD	0.42
11	*LOA1*	Lysophosphatidic acid acyltransferase	ER/LD/Cm	0.40
12	*ERG1*	Squalene epoxidase	ER/LB	0.38
13	*PDR16*	Phosphatidylinositol transfer protein	LD/Cm/PM	0.34
14	*YEH1*	Steryl ester hydrolase	LD/IntM	0.34
15	*HFD1*	Putative fatty aldehyde dehydrogenase	E/LD/M	0.32

Note: LD, lipid droplet; ER, endoplasmic reticulum; M, mitochondria; PM, plasma membrane; LB, lipid body; Cm, cytoplasm; intM, integral to membrane.

Table S2. Predicted LD-anchoring motifs identified by three algorithms. Proteins shaded in gray were excluded due to the absence of LD-anchoring motif prediction data. Residue ranges listed under the predicted and selected motifs correspond to amino acid positions within the full-length native protein sequences.

Protein	Class of LD	Predicted LD-anchoring motif (Phobius)	Predicted LD-anchoring motif (TMHMM)	Signal sequence (SignalP-5.0)	Selected residues for this study
Erg6	Class I	323-359	323-359	ND	323-359
Faa4	Class I	ND	ND	ND	-
Fat1	Class I	6-26	7-29	1-30	1-30
Tgl1	Class I	9-55	ND	ND	9-55
Pgc1	NR	297-315	297-315	ND	297-315
Ayr1	Class I	250-271	250-271	ND	250-271
Erg7	Class I	ND	ND	ND	-
Slc1	Class I	1-30	ND	1-30	1-30
Tgl3	Class I	ND	197-219	ND	197-219
Tgl5	Class I	ND	ND	ND	-
Loa1	Class I	31-53	31-53	ND	31-53
Erg1	Class I	441-496	ND	ND	441-496
Pdr16	NR	ND	ND	ND	-
Yeh1	NR	ND	ND	ND	-
Hfd1	NR	132-155	130-152	ND	130-155

Note: NR: Not reported, ND: Not detected.

Table S3. Fluorescence intensity of GFP fused to selected native LD-anchoring motifs at 24, 48, and 72 hr in wild-type yeast cells. Data are shown as mean ± standard deviation from three independent experiments.

	**GFP fluorescence intensity**										
	**Control**	**C-terminal LD-anchoring motifs**					**N-terminal LD-anchoring motifs**				
	**Eht1-GFP-Eht1**	**GFP-Erg6**	**GFP-Ayr1**	**GFP-Pgc1**	**GFP-Hfd1**	**GFP-Erg1**	**Tgl3-GFP**	**Fat1-GFP**	**Loa1-GFP**	**Slc1-GFP**	**Tgl1-GFP**
**24 hr**											
Ave. (a.u)	8.05 (± 3.08)	10.36 (± 0.55)	7.66 (± 3.32)	7.95 (± 2.76)	3.59 (± 0.96)	18.32 (± 0.71)	10.86 (± 1.64)	8.77 (± 0.84)	18.57 (± 8.89)	16.46 (± 8.39)	18.13 (± 2.04)
**48 hr**											
Ave. (a.u)	9.69 (± 0.74)	10.57 (± 1.16)	14.74 (± 4.91)	9.28 (± 1.09)	10.59 (± 3.91)	26.89 (± 5.64)	9.40 (± 1.09)	9.53 (± 1.48)	27.40 (± 7.91)	18.94 (± 4.96)	29.83 (± 2.27)
**72 hr**											
Ave. (a.u)	33.22 (± 3.42)	45.59 (± 1.35)	19.10 (± 5.55)	19.49 (± 7.38)	13.40 (± 3.59)	40.19 (± 7.12)	8.20 (± 1.38)	37.52 (± 2.35)	32.75 (± 7.94)	15.57 (± 4.99)	39.66 (± 2.87)

Table S4. Amino acid sequences of Erg1- and Tgl1-derived LD-anchoring motifs used in this study.

LD-anchoring motif	Amino acid sequence (residue numbers)	Length of sequence
Erg1	PLQLTRVFFAVAFYTIYLNMEERGFLGLPMALLEGIMILITAIRVFTPFLFGELIG (441-496)	56
$Erg1^{CYT}$	PLQLTRVFFAVAFYTIYLNMEERGFLGLP (441-469)	29
$Erg1^{CYT\text{-}H}$	PLQLTRVFFAVAFYTIYLNMEE (441-462)	22
$Erg1^{L}$	RGFLGLPMALLEGIMILITAIRVFTPFLFGELIG (463-496)	34
$Erg1^{L\text{-}H}$	MALLEGIMILITAIRVFTPFLFGELIG (470-496)	27
Tgl1	LSITDYIIVVLVYIESIISSVLKLIPQPMINLFEWLINFSTSSDDNT (9-55)	47
$Tgl1^{CYT}$	KLIPQPMINLFEWLINFSTSSDDNT (31-55)	25
$Tgl1^{CYT\text{-}H}$	MINLFEWLINFSTSSDDNT (37-55)	19
$Tgl1^{L}$	LSITDYIIVVLVYIESIISSVLKLIPQP (9-36)	28
$Tgl1^{L\text{-}H}$	LSITDYIIVVLVYIESIISSVL (9-30)	22

Table S5. Amino acid sequences of LD-anchoring constructs used for PDV biosynthesis.

**A^{CYT}**<br>Annotation: VioA-linker-Erg1^{CYT} LD-anchoring motif<br>MKHSSDICIVGAGISGLTCASHLLDSPACRGLSLRIFDMQQEAGGRIRSKMLDGKA<br>SIELGAGRYSPQLHPHFQSAMQHYSQKSEVYPFTQLKFKSHVQQKLKRAMNELSP<br>RLKEHGKESFLQFVSRYQGHDSAVGMIRSMGYDALFLPDISAEMAYDIVGKHPEIQ<br>SVTDNDANQWFAAETGFAGLIQGIKAKVKAAGARFSLGYRLLSVRTDGDGYLLQ<br>LAGDDGWKLEHRTRHLILAIPPSAMAGLNVDFPEAWSGARYGSLPLFKGFLTYGE<br>PWWLDYKLDDQVLIVDNPLRKIYFKGDKYLFFYTDSEMANYWRGCVAEGEDGY<br>LEQIRTHLASALGIVRERIPQPLAHVHKYWAHGVEFCRDSDIDHPSALSHRDSGIIA<br>CSDAYTEHCGWMEGGLLSAREASRLLLQRIAAGSGSPLQLTRVFFAVAFYTIYLN<br>MEERGFLGLP*
**A^L**<br>Annotation: VioA-linker-Erg1^L LD-anchoring motif<br>MKHSSDICIVGAGISGLTCASHLLDSPACRGLSLRIFDMQQEAGGRIRSKMLDGKA<br>SIELGAGRYSPQLHPHFQSAMQHYSQKSEVYPFTQLKFKSHVQQKLKRAMNELSP<br>RLKEHGKESFLQFVSRYQGHDSAVGMIRSMGYDALFLPDISAEMAYDIVGKHPEIQ<br>SVTDNDANQWFAAETGFAGLIQGIKAKVKAAGARFSLGYRLLSVRTDGDGYLLQ<br>LAGDDGWKLEHRTRHLILAIPPSAMAGLNVDFPEAWSGARYGSLPLFKGFLTYGE<br>PWWLDYKLDDQVLIVDNPLRKIYFKGDKYLFFYTDSEMANYWRGCVAEGEDGY<br>LEQIRTHLASALGIVRERIPQPLAHVHKYWAHGVEFCRDSDIDHPSALSHRDSGIIA<br>CSDAYTEHCGWMEGGLLSAREASRLLLQRIAAGSGSRGFLGLPMALLEGIMILITAI<br>RVFTPFLFGELIG*
**B^{CYT}**<br>Annotation: VioB-linker-Erg1^{CYT} LD-anchoring motif<br>MSILDFPRIHFRGWARVNAPTANRDPHGHIDMASNTVAMAGEPFDLARHPTEFHR<br>HLRSLGPRFGLDGRADPEGPFSLAEGYNAAGNNHFSWESATVSHVQWDGGEADR<br>GDGLVGARLALWGHYNDYLRTTFNRARWVDSDPTRRDAAQIYAGQFTISPAGAG<br>PGTPWLFTADIDDSHGARWTRGGHIAERGGHFLDEEFGLARLFQFSVPKDHPHFLF<br>HPGPFDSEAWRRLQLALEDDDVLGLTVQYALFNMSTPPQPNSPVFHDMVGVVGL<br>WRRGELASYPAGRLLRPRQPGLGDLTLRVNGGRVALNLACAIPFSTRAAQPSAPD<br>RLTPDLGAKLPLGDLLLRDEDGALLARVPQALYQDYWTNHGIVDLPLLREPRGSL<br>TLSSELAEWREQDWVTQSDASNLYLEAPDRRHGRFFPESIALRSYFRGEARARPDI<br>PHRIEGMGLVGVESRQDGDAAEWRLTGLRPGPARIVLDDGAEAIPLRVLPDDWAL<br>DDATVEEVDYAFLYRHVMAYYELVYPFMSDKVFSLADRCKCETYARLMWQMC<br>DPQNRNKSYYMPSTRELSAPKARLFLKYLAHVEGQARLQAPPPAGPARIESKAQL<br>AAELRKAVDLELSVMLQYLYAAYSIPNYAQGQQRVRDGAWTAEQLQLACGSGD<br>RRRDGGIRAALLEIAHEEMIHYLVVNNLLMALGEPFYAGVPLMGEAARQAFGLDT<br>EFALEPFSESTLARFVRLEWPHFIPAPGKSIADCYAAIRQAFLDLPDLFGGEAGKRG<br>GEHHLFLNELTNRAHPGYQLEVFDRDSALFGIAFVTDQGEGGALDSPHYEHSHFQ<br>RLREMSARIMAQSAPFEPALPALRNPVLDESPGCQRVADGRARALMALYQGVYEL<br>MFAMMAQHFAVKPLGSLRRSRLMNAAIDLMTGLLRPLSCALMNLPSGIAGRTAG<br>PPLPGPVDTRSYDDYALGCRMLARRCERLLEQASMLEPGWLPDAQMELLDFYRR<br>QMLDLACGKLSREAGSGSPLQLTRVFFAVAFYTIYLNMEERGFLGLP*

B^L

Annotation: VioB-linker-Erg1^L LD-anchoring motif

MSILDFPRIHFRGWARVNAPTANRDPHGHIDMASNTVAMAGEPFDLARHPTEFHR
HLRSLGPRFGLDGRADPEGPFSLAEGYNAAGNNHFSWESATVSHVQWDGGEADR
GDGLVGARLALWGHYNDYLRTTFNRARWVDSDPTRRDAAQIYAGQFTISPAGAG
PGTPWLFTADIDDSHGARWTRGGHIAERGGHFLDEEFGLARLFQFSVPKDHPHFLF
HPGPFDSEAWRRLQLALEDDDVLGLTVQYALFNMSTPPQPNSPVFHDMVGVVGL
WRRGELASYPAGRLLRPRQPGLGDLTLRVNGGRVALNLACAIPFSTRAAQPSAPD
RLTPDLGAKLPLGDLLLRDEDGALLARVPQALYQDYWTNHGIVDLPLLREPRGSL
TLSSELAEWREQDWVTQSDASNLYLEAPDRRHGRFFPESIALRSYFRGEARARPDI
PHRIEGMGLVGVESRQDGDAAEWRLTGLRPGPARIVLDDGAEAIPLRVLPDDWAL
DDATVEEVDYAFLYRHVMAYYELVYPFMSDKVFSLADRCKCETYARLMWQMC
DPQNRNKSYYMPSTRELSAPKARLFLKYLAHVEGQARLQAPPPAGPARIESKAQL
AAELRKAVDLELSVMLQYLYAAYSIPNYAQGQQRVRDGAWTAEQLQLACGSGD
RRRDGGIRAALLEIAHEEMIHYLVVNNLLMALGEPFYAGVPLMGEAARQAFGLDT
EFALEPFSESTLARFVRLEWPHFIPAPGKSIADCYAAIRQAFLDLPDLFGGEAGKRG
GEHHLFLNELTNRAHPGYQLEVFDRDSALFGIAFVTDQGEGGALDSPHYEHSHFQ
RLREMSARIMAQSAPFEPALPALRNPVLDESPGCQRVADGRARALMALYQGVYEL
MFAMMAQHFAVKPLGSLRRSRLMNAAIDLMTGLLRPLSCALMNLPSGIAGRTAG
PPLPGPVDTRSYDDYALGCRMLARRCERLLEQASMLEPGWLPDAQMELLDFYRR
QMLDLACGKLSREAGSGSRGFLGLPMALLEGIMILITAIRVFTPFLFGELIG*

E^CYT

Annotation: Tgl1^CYT LD-anchoring motif-linker-VioE

MKLIPQPMINLFEWLINFSTSSDDNTGSGSENREPPLLPARWSSAYVSYWSPMLPD
DQLTSGYCWFDYERDICRIDGLFNPWSERDTGYRLWMSEVGNAASGRTWKQKV
AYGRERTALGEQLCERPLDDETGPFAELFLPRDVLRRLGARHIGRRVVLGREADG
WRYQRPGKGPSTLYLDAASGTPLRMVTGDEASRASLRDFPNVSEAEIPDAVFAAK
R*

E^L

Annotation: Tgl1^L LD-anchoring motif-linker-VioE

MLSITDYIIVVLVYIESIISSVLKLIPQPGSGSENREPPLLPARWSSAYVSYWSPMLPD
DQLTSGYCWFDYERDICRIDGLFNPWSERDTGYRLWMSEVGNAASGRTWKQKV
AYGRERTALGEQLCERPLDDETGPFAELFLPRDVLRRLGARHIGRRVVLGREADG
WRYQRPGKGPSTLYLDAASGTPLRMVTGDEASRASLRDFPNVSEAEIPDAVFAAK
R*

Table S6. Comparison of PDV production and cell growth in the wild-tyle (WT) yeast strains expressing various combinations of LD-anchored PDV biosynthetic enzymes. Yeast strains were cultivated in 10 mL of defined minimal media containing 2% (w/v) glucose, 608 mg/L L-tryptophan, and 40 µM ammonium iron (II) sulfate for 144 h at 30 °C in a 50 mL bioreactor. −OA and +OA indicate conditions without and with 300 µM oleic acid supplementation, respectively.

	PDV titer (mg L^{-1})		Cell growth (OD_{600})		PDV contents (mg g^{-1} DCW)	
	- OA	+ OA	- OA	+ OA	- OA	+ OA
**WT-ABE**	1.14 (± 0.31)	2.20 (± 1.10)	19.67 (± 1.36)	19.17 (± 0.77)	0.06 (± 0.02)	0.12 (± 0.06)
**WT-A^{CYT}BE**	3.38 (± 0.09)	5.74 (± 0.48)	18.15 (± 0.76)	18.40 (± 0.63)	0.19 (± 0.00)	0.31 (± 0.02)
**WT-A^{CYT}B^{CYT}E**	0.00 (± 0.00)	0.00 (± 0.00)	18.23 (± 1.34)	19.22 (± 1.14)	0.00 (± 0.00)	0.00 (± 0.00)
**WT-A^{CYT}BE^{CYT}**	0.00 (± 0.00)	0.00 (± 0.00)	19.36 (± 1.25)	19.25 (± 0.89)	0.00 (± 0.00)	0.00 (± 0.00)
**WT-A^{CYT}B^{L}E**	0.00 (± 0.00)	0.00 (± 0.00)	19.44 (± 0.32)	19.62 (± 0.46)	0.00 (± 0.00)	0.00 (± 0.00)
**WT-A^{CYT}BE^{L}**	0.00 (± 0.00)	0.00 (± 0.00)	19.33 (± 0.31)	19.75 (± 1.40)	0.00 (± 0.00)	0.00 (± 0.00)
**WT-AB^{CYT}E**	0.65 (± 0.08)	1.04 (± 0.16)	19.15 (± 1.68)	18.71 (± 0.67)	0.03 (± 0.00)	0.06 (± 0.01)
**WT-AB^{CYT}E^{CYT}**	0.00 (± 0.00)	0.00 (± 0.00)	19.35 (± 0.29)	19.49 (± 0.36)	0.00 (± 0.00)	0.00 (± 0.00)
**WT-AB^{CYT}E^{L}**	0.00 (± 0.00)	0.00 (± 0.00)	20.07 (± 1.09)	19.15 (± 1.35)	0.00 (± 0.00)	0.00 (± 0.00)
**WT-ABE^{CYT}**	11.34 (± 0.56)	11.54 (± 0.87)	19.12 (± 1.13)	16.64 (± 0.78)	0.59 (± 0.05)	0.07 (± 0.08)
**WT-A^{L}BE**	0.00 (± 0.00)	0.00 (± 0.00)	19.91 (± 0.06)	20.04 (± 0.66)	0.00 (± 0.00)	0.00 (± 0.00)
**WT-A^{L}B^{CYT}E**	0.00 (± 0.00)	0.00 (± 0.00)	19.52 (± 0.07)	19.47 (± 0.19)	0.00 (± 0.00)	0.00 (± 0.00)
**WT-A^{L}BE^{CYT}**	0.00 (± 0.00)	0.00 (± 0.00)	20.17 (± 0.23)	19.49 (± 0.37)	0.00 (± 0.00)	0.00 (± 0.00)
**WT-A^{L}B^{L}E**	0.00 (± 0.00)	0.00 (± 0.00)	19.31 (± 0.11)	18.71 (± 0.21)	0.00 (± 0.00)	0.00 (± 0.00)
**WT-A^{L}BE^{L}**	0.00 (± 0.00)	0.00 (± 0.00)	20.61 (± 0.24)	20.47 (± 1.02)	0.00 (± 0.00)	0.00 (± 0.00)
**WT-AB^{L}E**	0.00 (± 0.00)	0.00 (± 0.00)	20.64 (± 0.29)	19.43 (± 0.31)	0.00 (± 0.00)	0.00 (± 0.00)
**WT-AB^{L}E^{CYT}**	0.00 (± 0.00)	0.00 (± 0.00)	20.87 (± 0.08)	20.67 (± 0.08)	0.00 (± 0.00)	0.00 (± 0.00)
**WT-AB^{L}E^{L}**	0.00 (± 0.00)	0.00 (± 0.00)	18.12 (± 0.37)	17.87 (± 0.22)	0.00 (± 0.00)	0.00 (± 0.00)
**WT-ABE^{L}**	14.09 (± 0.60)	19.69 (± 0.42)	17.95 (± 0.96)	17.61 (± 0.54)	0.79 (± 0.01)	1.12 (± 0.05)
**WT-A^{CYT}B^{CYT}E^{CYT}**	0.00 (± 0.00)	0.00 (± 0.00)	19.47 (± 0.08)	19.52 (± 0.35)	0.00 (± 0.00)	0.00 (± 0.00)
**WT-A^{CYT}B^{CYT}E^{L}**	0.00 (± 0.00)	0.00 (± 0.00)	20.31 (± 0.66)	19.54 (± 0.33)	0.00 (± 0.00)	0.00 (± 0.00)
**WT-A^{CYT}B^{L}E^{CYT}**	0.00 (± 0.00)	0.00 (± 0.00)	20.99 (± 0.38)	20.03 (± 0.19)	0.00 (± 0.00)	0.00 (± 0.00)

**WT-$A^{L}B^{CYT}E^{CYT}$**	0.00 (± 0.00)	0.00 (± 0.00)	19.36 (± 0.29)	19.30 (± 0.29)	0.00 (± 0.00)	0.00 (± 0.00)
**WT-$A^{L}B^{L}E^{CYT}$**	0.00 (± 0.00)	0.00 (± 0.00)	19.57 (± 0.30)	19.54 (± 0.46)	0.00 (± 0.00)	0.00 (± 0.00)
**WT-$A^{L}B^{CYT}E^{L}$**	0.00 (± 0.00)	0.00 (± 0.00)	19.68 (± 0.96)	19.56 (± 0.46)	0.00 (± 0.00)	0.00 (± 0.00)
**WT-$A^{CYT}B^{L}E^{L}$**	0.00 (± 0.00)	0.00 (± 0.00)	19.61 (± 0.54)	19.67 (± 0.19)	0.00 (± 0.00)	0.00 (± 0.00)
**WT-$A^{L}B^{L}E^{L}$**	0.00 (± 0.00)	0.00 (± 0.00)	20.00 (± 0.21)	20.61 (± 0.18)	0.00 (± 0.00)	0.00 (± 0.00)

Table S7. Comparison of PDV production and cell growth in our previous LD-engineered yeast strains expressing various combinations of LD-anchored PDV biosynthetic enzymes. Yeast strains were cultivated in 10 mL of defined minimal media containing 2% (w/v) glucose, 608 mg/L L-tryptophan, and 40 µM ammonium iron (II) sulfate for 144 h at 30 °C in a 50 mL bioreactor. −OA and +OA indicate conditions without and with 300 µM oleic acid supplementation, respectively.

	**PDV titer ($mg\ L^{-1}$)**		**Cell growth (OD_{600})**		**PDV contents ($mg\ g^{-1}$ DCW)**	
	**- OA**	**+ OA**	**- OA**	**+ OA**	**- OA**	**+ OA**
**ABE**	3.45 (± 0.34)	3.50 (± 0.13)	19.44 (± 0.62)	17.33 (± 0.71)	0.18 (± 0.01)	0.20 (± 0.00)
**$A^{CYT}BE$**	7.71 (± 0.37)	8.63 (± 0.30)	19.97 (± 0.40)	20.80 (± 0.71)	0.39 (± 0.03)	0.42 (± 0.03)
**$A^{CYT}B^{CYT}E$**	1.04 (± 0.09)	0.95 (± 0.04)	19.37 (± 0.23)	21.80 (± 0.89)	0.05 (± 0.00)	0.04 (± 0.00)
**$A^{CYT}BE^{CYT}$**	7.40 (± 0.31)	7.90 (± 0.78)	20.01 (± 1.22)	21.12 (± 1.04)	0.37 (± 0.03)	0.37 (± 0.04)
**$A^{CYT}B^{L}E$**	0.00 (± 0.00)	0.00 (± 0.00)	19.95 (± 0.72)	19.95 (± 0.53)	0.00 (± 0.00)	0.00 (± 0.00)
**$A^{CYT}BE^{L}$**	17.13 (± 0.33)	16.90 (± 1.10)	20.33 (± 0.95)	21.71 (± 1.43)	0.84 (± 0.05)	0.78 (± 0.03)
**$AB^{CYT}E$**	1.83 (± 0.09)	1.78 (± 0.15)	20.59 (± 0.41)	20.20 (± 0.98)	0.09 (± 0.00)	0.09 (± 0.00)
**$AB^{CYT}E^{CYT}$**	4.09 (± 0.66)	3.83 (± 0.17)	20.45 (± 0.61)	21.43 (± 0.77)	0.20 (± 0.03)	0.18 (± 0.00)
**$AB^{CYT}E^{L}$**	7.08 (± 1.63)	7.32 (± 0.44)	20.47 (± 0.46)	21.05 (± 1.12)	0.34 (± 0.07)	0.35 (± 0.01)
**ABE^{CYT}**	24.49 (± 0.74)	23.09 (± 2.32)	19.64 (± 1.07)	18.95 (± 1.10)	1.25 (± 0.07)	1.22 (± 0.06)
**$A^{L}BE$**	0.00 (± 0.00)	0.00 (± 0.00)	19.67 (± 1.62)	18.83 (± 0.69)	0.00 (± 0.00)	0.00 (± 0.00)
**$A^{L}B^{CYT}E$**	0.00 (± 0.00)	0.00 (± 0.00)	19.99 (± 0.80)	19.35 (± 0.48)	0.00 (± 0.00)	0.00 (± 0.00)
**$A^{L}BE^{CYT}$**	0.00 (± 0.00)	0.00 (± 0.00)	20.17 (± 0.23)	19.74 (± 0.37)	0.00 (± 0.00)	0.00 (± 0.00)
**$A^{L}B^{L}E$**	0.00 (± 0.00)	0.00 (± 0.00)	19.86 (± 0.20)	19.11 (± 0.85)	0.00 (± 0.00)	0.00 (± 0.00)
**$A^{L}BE^{L}$**	0.00 (± 0.00)	0.00 (± 0.00)	19.57 (± 0.74)	19.38 (± 0.61)	0.00 (± 0.00)	0.00 (± 0.00)
**$AB^{L}E$**	0.00 (± 0.00)	0.00 (± 0.00)	19.63 (± 0.96)	19.75 (± 0.45)	0.00 (± 0.00)	0.00 (± 0.00)
**$AB^{L}E^{CYT}$**	0.00 (± 0.00)	0.00 (± 0.00)	20.16 (± 0.60)	19.72 (± 0.59)	0.00 (± 0.00)	0.00 (± 0.00)
**$AB^{L}E^{L}$**	0.00 (± 0.00)	0.00 (± 0.00)	19.41 (± 0.99)	19.59 (± 0.39)	0.00 (± 0.00)	0.00 (± 0.00)
**ABE^{L}**	46.25 (± 2.91)	43.36 (± 0.95)	20.51 (± 0.51)	20.40 (± 0.88)	2.25 (± 0.09)	2.13 (± 0.07)
**$A^{CYT}B^{CYT}E^{CYT}$**	0.00 (± 0.00)	0.00 (± 0.00)	21.08 (± 0.72)	20.33 (± 0.95)	0.00 (± 0.00)	0.00 (± 0.00)
**$A^{CYT}B^{CYT}E^{L}$**	3.61 (± 0.41)	3.34 (± 0.06)	20.77 (± 0.49)	20.35 (± 0.26)	0.17 (± 0.02)	0.16 (± 0.00)
**$A^{CYT}B^{L}E^{CYT}$**	0.00 (± 0.00)	0.00 (± 0.00)	20.41 (± 0.36)	19.55 (± 0.65)	0.00 (± 0.00)	0.00 (± 0.00)

**A^{L}B^{CYT}E^{CYT}**	0.00 (± 0.00)	0.00 (± 0.00)	19.87 (± 0.69)	19.32 (± 0.59)	0.00 (± 0.00)	0.00 (± 0.00)
**A^{L}B^{L}E^{CYT}**	0.00 (± 0.00)	0.00 (± 0.00)	20.13 (± 0.65)	19.40 (± 0.17)	0.00 (± 0.00)	0.00 (± 0.00)
**A^{L}B^{CYT}E^{L}**	0.00 (± 0.00)	0.00 (± 0.00)	21.43 (± 1.29)	20.33 (± 0.42)	0.00 (± 0.00)	0.00 (± 0.00)
**A^{CYT}B^{L}E^{L}**	0.00 (± 0.00)	0.00 (± 0.00)	20.78 (± 0.38)	20.24 (± 0.34)	0.00 (± 0.00)	0.00 (± 0.00)
**A^{L}B^{L}E^{L}**	0.00 (± 0.00)	0.00 (± 0.00)	20.71 (± 0.77)	20.42 (± 0.55)	0.00 (± 0.00)	0.00 (± 0.00)

Table S8. Total LD surface area and volume per cell in the LD-engineered yeast strain (*9*)

	Wild-type strain	LD-engineered strain
**The average diameter of LDs per cell (μm)**	0.200	0.330
**The average number of LDs per cell**	23.000	15.400
**The total volume of LDs per cell (μm^3)**	0.092	0.293
**The total surface area of LDs per cell (μm^2)**	2.898	5.267

Table S9. In vitro PDV production and maximum velocity of yeast lysates expressing LD-anchored PDV biosynthetic enzymes. PDV production was monitored in vitro by measuring PDV intensity (mAU) at indicated time points (0–120 minutes) using yeast lysates expressing various LD-anchored biosynthetic enzymes. Maximum velocity (mAU min^{-1}) was calculated based on the initial linear phase (0-10 min).

	In vitro PDV intensity (mAU)											Maximum velocity (mAU min^{-1})
	**0 min**	**1 min**	**3 min**	**5 min**	**10 min**	**20 min**	**30 min**	**40 min**	**50 min**	**60 min**	**120 min**	
**ABE**	0.00	14.85	23.84	25.30	23.82	23.25	24.05	24.27	25.24	24.21	25.54	1.87
**$A^{CYT}BE$**	0.00	84.48	91.08	111.48	118.84	119.84	118.19	120.39	121.82	120.78	122.55	8.94
**$AB^{CYT}E$**	0.00	5.08	6.58	11.20	16.17	16.62	14.11	17.83	17.25	18.20	16.35	1.5
**ABE^{CYT}**	0.00	146.82	152.96	174.41	191.63	193.66	192.28	193.47	192.38	196.32	193.43	13.76
**$A^{L}BE$**	0.00	0.00	0.00	0.00	0.00	0.00	0.00	0.00	0.00	0.00	0.00	0
**$AB^{L}E$**	0.00	0.00	0.00	0.00	0.00	0.00	0.00	0.00	0.00	0.00	0.00	0
**ABE^{L}**	0.00	137.30	168.69	183.56	191.65	194.76	193.22	194.30	192.29	195.52	193.92	14.16

Table S10. Amino acid sequences of LD-anchoring constructs used for lycopene biosynthesis

crtE^CYT

Annotation: crtE-linker-Erg1^CYT LD-anchoring motif

MDYANILTAIPLEFTPQDDIVLLEPYHYLGKNPGKEIRSQLIEAFNYWLDVKKEDLE
VIQNVVGMLHTASLLMDDVEDSSVLRRGSPVAHLIYGIPQTINTANYVYFLAYQEI
FKLRPTPIPMPVIPPSSASLQSSVSSASSSSSASSENGGTSTPNSQIPFSKDTYLDKVIT
DEILSLHRGQGLELFWRDSLTCPSEEEYVKMVLGKTGGLFRIAVRLMMAKSECDI
DFVQLVNLISIYFQIRDDYMNLQSSEYAHNKNFAEDLTEGKFSFPTIHSIHANPSSRL
VINTLQKKSTSPEILHHCVNYMRTETHSFEYTQEVLNTLSGALERELGRLQGEFAE
ANSRMDLGDVDSEGRTGKNVKLEAILKKLADIPLGGGGSGGGGSGGGGSPLQLTR
VFFAVAFYTIYLNMEERGFLGLP*

crtE^L

Annotation: crtE-linker-Erg1^L LD-anchoring motif

MDYANILTAIPLEFTPQDDIVLLEPYHYLGKNPGKEIRSQLIEAFNYWLDVKKEDLE
VIQNVVGMLHTASLLMDDVEDSSVLRRGSPVAHLIYGIPQTINTANYVYFLAYQEI
FKLRPTPIPMPVIPPSSASLQSSVSSASSSSSASSENGGTSTPNSQIPFSKDTYLDKVIT
DEILSLHRGQGLELFWRDSLTCPSEEEYVKMVLGKTGGLFRIAVRLMMAKSECDI
DFVQLVNLISIYFQIRDDYMNLQSSEYAHNKNFAEDLTEGKFSFPTIHSIHANPSSRL
VINTLQKKSTSPEILHHCVNYMRTETHSFEYTQEVLNTLSGALERELGRLQGEFAE
ANSRMDLGDVDSEGRTGKNVKLEAILKKLADIPLGGGGSGGGGSGGGGSRGFLGL
PMALLEGIMILITAIRVFTPFLF*

crtB^CYT

Annotation: Tgl1^CYT LD-anchoring motif-linker-crtB

MKLIPQPMINLFEWLINFSTSSDDNTRTIYGNKKMPSSFPLITPPVLSLFFSSRPYSSQ
PKRDLELAVKLLEKKSRSFFVASAGFPSEVRERLVGLYAFCRVTDDLIDSPEVSSNP
HATIDMVSDFLTLLFGPPLHPSQPDKILSSPLLPPSHPSRPTGMYPLPPPPSLSPAELV
QFLTERVPVQYHFAFRLLAKLQGLIPRYPLDELLRGYTTDLIFPLSTEAVQARKTPIE
TTADLLDYGLCVAGSVAELLVYYVSWASAPSQVPATIEEREAVLVASREMGTALQL
VNIARDIKGDATEGRFYLPLSFFGLRDESKLAIPTDWTEPRPQDFDKLLSLSPSSTLP
SSNASESFRFEWKTYSLPLVAYAEDLAKHSYKGIDRLPTEVQAGMRAACASYLLIG
REIKVVWKGDVGERRTVAGWRRVRKVLSVVMSGWEGQ*

crtB^L

Annotation: Tgl1^L LD-anchoring motif-linker-crtB

MTDYIIVVLVYIESIISSVLKLIPQPRTIYGNKKMPSSFPLITPPVLSLFFSSRPYSSQPK
RDLELAVKLLEKKSRSFFVASAGFPSEVRERLVGLYAFCRVTDDLIDSPEVSSNPHA
TIDMVSDFLTLLFGPPLHPSQPDKILSSPLLPPSHPSRPTGMYPLPPPPSLSPAELVQF
LTERVPVQYHFAFRLLAKLQGLIPRYPLDELLRGYTTDLIFPLSTEAVQARKTPIETT
ADLLDYGLCVAGSVAELLVYYVSWASAPSQVPATIEEREAVLVASREMGTALQLV
NIARDIKGDATEGRFYLPLSFFGLRDESKLAIPTDWTEPRPQDFDKLLSLSPSSTLPS
SNASESFRFEWKTYSLPLVAYAEDLAKHSYKGIDRLPTEVQAGMRAACASYLLIG
REIKVVWKGDVGERRTVAGWRRVRKVLSVVMSGWEGQ*

crtI^CYT

Annotation: Tgl1^CYT LD-anchoring motif-linker-crtI

MKLIPQPMINLFEWLINFSTSSDDNTEAAAKEAAAKEAAAKEAAAKGKEQDQDKP
TAIIVGCGIGGIATAARLAKEGFQVTVFEKNDYSGGRCSLIERDGYRFDQGPSLLLL
PDLFKQTFEDLGEKMEDWVDLIKCEPNYVCHFHDEETFTFSTDMALLKREVERFE
GKDGFDRFLSFIQEAHRHYELAVVHVLQKNFPGFAAFLRLQFIGQILALHPFESIWT
RVCRYFKTDRLRRVFSFAVMYMGQSPYSAPGTYSLLQYTELTEGIWYPRGGFWQ
VPNTLLQIVKRNNPSAKFNFNAPVSQVLLSPAKDRATGVRLESGEEHHADVVIVN
ADLVYASEHLIPDDARNKIGQLGEVKRSWWADLVGGKKLKGSCSSLSFYWSMDR
IVDGLGGHNIFLAEDFKGSFDTIFEELGLPADPSFYVNVPSRIDPSAAPEGKDAIVIL
VPCGHIDASNPQDYNKLVARARKFVIQTLSAKLGLPDFEKMIVAEKVHDAPSWEK
EFNLKDGSILGLAHNFMQVLGFRPSTRHPKYDKLFFVGASTHPGTGVPIVLAGAKL
TANQVLESFDRSPAPDPNMSLSVPYGKPLKSNGTGIDSQVQLKFMDLER*

crtI^{L}

Annotation: Tgl1^{L} LD-anchoring motif-linker-crtI

MTDYIIVVLVYIESIISSVLKLIPQPEAAAKEAAAKEAAAKEAAAKGKEQDQDKPT
AIIVGCGIGGIATAARLAKEGFQVTVFEKNDYSGGRCSLIERDGYRFDQGPSLLLLP
DLFKQTFEDLGEKMEDWVDLIKCEPNYVCHFHDEETFTFSTDMALLKREVERFEG
KDGFDRFLSFIQEAHRHYELAVVHVLQKNFPGFAAFLRLQFIGQILALHPFESIWTR
VCRYFKTDRLRRVFSFAVMYMGQSPYSAPGTYSLLQYTELTEGIWYPRGGFWQVP
NTLLQIVKRNNPSAKFNFNAPVSQVLLSPAKDRATGVRLESGEEHHADVVIVNAD
LVYASEHLIPDDARNKIGQLGEVKRSWWADLVGGKKLKGSCSSLSFYWSMDRIV
DGLGGHNIFLAEDFKGSFDTIFEELGLPADPSFYVNVPSRIDPSAAPEGKDAIVILVP
CGHIDASNPQDYNKLVARARKFVIQTLSAKLGLPDFEKMIVAEKVHDAPSWEKEF
NLKDGSILGLAHNFMQVLGFRPSTRHPKYDKLFFVGASTHPGTGVPIVLAGAKLTA
NQVLESFDRSPAPDPNMSLSVPYGKPLKSNGTGIDSQVQLKFMDLER*

Table S11. In vivo lycopene production and cell growth of yeast strains expressing LD-anchored lycopene biosynthetic enzymes. Yeast strains were cultivated in 50 mL of YP medium containing 2% (w/v) glucose for 144 h at 30 °C in 250 mL flasks. Data are presented as mean ± standard deviation from three independent experiments.

	**Lycopene titer ($mg\ L^{-1}$)**	**Cell growth (OD_{600})**	**Lycopene contents ($mg\ g^{-1}$ DCW)**
**EBI**	13.04 (± 0.95)	92.00 (± 3.49)	0.68 (± 0.07)
**$E^{CYT}BI$**	16.85 (± 1.13)	88.27 (± 0.46)	0.92 (± 0.06)
**$E^{L}BI$**	12.91 (± 2.25)	90.67 (± 1.85)	0.68 (± 0.12)
**$EB^{CYT}I$**	14.16 (± 1.85)	101.07 (± 0.46)	0.67 (± 0.09)
**$EB^{L}I$**	13.97 (± 2.71)	98.40 (± 0.80)	0.68 (± 0.13)
**EBI^{CYT}**	10.58 (± 2.83)	96.27 (± 1.22)	0.53 (± 0.13)
**EBI^{L}**	31.12 (± 1.73)	90.13 (± 4.03)	1.66 (± 0.15)

Table S12. In vitro GGPP biosynthesis by LD-anchored CrtE variants involved in the initial step of lycopene biosynthesis. In vitro GGPP production was analyzed by measuring GGPP intensity (mAU) at the indicated time points (0-120 min) in yeast lysates expressing LD-anchored CrtE variants. Maximum velocities (mAU min^{-1}) were calculated from the initial linear reaction phase (0-10 min).

	In vitro GGPP intensity (mAU)											Maximum velocity (mAU min^{-1})
	**0 min**	**1 min**	**3 min**	**5 min**	**10 min**	**20 min**	**30 min**	**40 min**	**50 min**	**60 min**	**120 min**	
**crtE**	0	8.12	14.37	19.84	23.46	24.12	23.88	24.35	24.09	24.18	24.41	2.15
**$crtE^{CYT}$**	0	12.84	21.06	29.74	36.28	37.91	37.42	38.05	37.68	37.84	38.12	3.31
**$crtE^{CYT\text{-}H}$**	0	7.95	13.92	18.52	23.02	23.64	23.37	23.71	23.55	23.48	23.66	2.09
**$crtE^{L}$**	0	0	1.18	2.34	5.08	5.24	5.11	5.37	5.26	5.19	5.42	0.53

Table S13. Quantification of in vitro PDV and GGPP production by LD-anchored VioE and CrtE variants with or without Proteinase K treatment. −PK and +PK indicate conditions without and with Proteinase K treatment, respectively (see Methods S2 for details).

	PDV intensity (mAU)	
	Untreated (−PK)	0.5 mg/mL Proteinase K treated (+PK)
VioE	13.25 (± 0.28)	ND
$VioE^{CYT}$	103.54 (± 2.37)	ND
$VioE^{CYT\text{-}H}$	13.03 (± 0.11)	ND
$VioE^{L}$	107.92 (± 1.17)	97.20 (± 0.77)

	GGPP intensity (mAU)	
	Untreated (−PK)	0.5 mg/mL Proteinase K treated (+PK)
crtE	23.46 (± 1.85)	ND
$crtE^{CYT}$	36.28 (± 1.51)	ND
$crtE^{CYT\text{-}H}$	23.02 (± 1.32)	ND
$crtE^{L}$	5.08 (± 0.30)	3.38 (± 0.28)

Note: ND: Not detected

Table S14. Comparison of expression levels of fluorescent proteins anchored to LD surfaces using designed LD-anchoring motifs. Relative fluorescence intensities were analyzed, as shown in Fig. S14.

		BFP	GFP	mCherry
**Cytosolic expression**	BGC	1.00 (± 0.01)	1.00 (± 0.03)	1.00 (± 0.00)
**Anchoring of a single fluorescent protein to LD surfaces**	BGC^{CYT}	0.94 (± 0.04)	0.91 (± 0.06)	0.53 (± 0.06)
	BGC^{L}	0.91 (± 0.07)	0.92 (± 0.02)	0.52 (± 0.02)
**Dual anchoring of two fluorescent proteins to LD surfaces**	$B^{CYT}GC^{CYT}$	0.64 (± 0.08)	0.85 (± 0.03)	0.49 (± 0.02)
	$B^{CYT}GC^{L}$	0.69 (± 0.04)	0.87 (± 0.01)	0.46 (± 0.06)
**Triple anchoring of three fluorescent proteins to LD surfaces**	$B^{CYT}G^{CYT}C^{CYT}$	0.36 (± 0.05)	0.24 (± 0.04)	0.25 (± 0.03)
	$B^{CYT}G^{CYT}C^{L}$	0.38 (± 0.04)	0.19 (± 0.02)	0.27 (± 0.02)

Table S15. Comparison of expression levels of fluorescent proteins anchored to LD surfaces using designed LD-anchoring motifs. Relative western blot band intensities were analyzed after normalization to GAPDH and subsequent normalization to the corresponding cytosolic expression controls, as shown in Fig. 5C.

		BFP	GFP	mCherry
**Cytosolic expression**	BGC	1.00	1.00	1.00
**Anchoring of a single fluorescent protein to LD surfaces**	BGC^{CYT}	1.13	0.94	0.68
	BGC^{L}	0.99	0.94	0.55
**Dual anchoring of two fluorescent proteins to LD surfaces**	$B^{CYT}GC^{CYT}$	0.61	0.80	0.49
	$B^{CYT}GC^{L}$	0.53	0.88	0.57
**Triple anchoring of three fluorescent proteins to LD surfaces**	$B^{CYT}G^{CYT}C^{CYT}$	0.22	0.34	0.15
	$B^{CYT}G^{CYT}C^{L}$	0.25	0.37	0.14

Table S16. List of plasmids used in this study

Plasmids	Description / Genotype	Reference
pRS416	Expression vector containing *GPD1* promoter, CEN/ARS single-copy origin and *URA3* marker	(*93*)
p416-P_{TEF1}-Eht1-GFP	p416-P_{TEF1}-Eht1(1-13)-GFP-Eht1(429-451)	This study
p416-P_{TEF1}-Erg6-GFP	p416-P_{TEF1}-Erg6(323-359)-GFP	This study
p416-P_{TEF1}-Tgl3-GFP	p416-P_{TEF1}-Tgl3(197-219)-GFP	This study
p416-P_{TEF1}-Ayr1-GFP	p416-P_{TEF1}-Ayr1(250-271)-GFP	This study
p416-P_{TEF1}-Pgc1-GFP	p416-P_{TEF1}-Pgc1(297-315)-GFP	This study
p416-P_{TEF1}-Hfd1-GFP	p416-P_{TEF1}-Hfd1(130-155)-GFP	This study
p416-P_{TEF1}-Erg1-GFP	p416-P_{TEF1}-Erg1(441-496)-GFP	This study
p416-P_{TEF1}-Fat1-GFP	p416-P_{TEF1}-Fat1(1-30)-GFP	This study
p416-P_{TEF1}-Loa1-GFP	p416-P_{TEF1}-Loa1(31-53)-GFP	This study
p416-P_{TEF1}-Slc1-GFP	p416-P_{TEF1}-Slc1(1-30)-GFP	This study
p416-P_{TEF1}-Tgl1-GFP	p416-P_{TEF1}-Tgl1(9-55)-GFP	This study
p416-P_{TEF1}-$Erg1^{CYT}$-GFP	p416-P_{TEF1}-Erg1(441-469)-GFP	This study
p416-P_{TEF1}-$Erg1^{CYT-H}$-GFP	p416-P_{TEF1}-Erg1(441-462)-GFP	This study
p416-P_{TEF1}-$Erg1^{L}$-GFP	p416-P_{TEF1}-Erg1(463-496)-GFP	This study
p416-P_{TEF1}-$Erg1^{L-H}$-GFP	p416-P_{TEF1}-Erg1(470-496)-GFP	This study
p416-P_{TEF1}-$Tgl1^{CYT}$-GFP	p416-P_{TEF1}-Tgl1(31-55)-GFP	This study
p416-P_{TEF1}-$Tgl1^{CYT-H}$-GFP	p416-P_{TEF1}-Tgl1(37-55)-GFP	This study
p416-P_{TEF1}-$Tgl1^{L}$-GFP	p416-P_{TEF1}-Tgl1(9-36)-GFP	This study
p416-P_{TEF1}-$Tgl1^{L-H}$-GFP	p416-P_{TEF1}-Tgl1(9-30)-GFP	This study
pJAS1052	Harboring PDV biosynthesis genes (*VioA* and *VioB*)	Addgene (*47*)
pJAS1669	Harboring PDV biosynthesis genes (*VioE*)	Addgene (*47*)

p416-AB	p416-P_{CCW12}-VioA-T_{ENO1}-P_{TEF1}-VioB-T_{CYC1}	This study
p416-ABE	p416-P_{CCW12}-VioA-T_{ENO1}-P_{TEF1}-VioB-T_{CYC1}-P_{GPD1}-VioE-T_{CYC1}	This study
p416-$A^{CYT}BE$	p416-P_{CCW12}-VioA- Erg1(441-469)-T_{ENO1}-P_{TEF1}-VioB-T_{CYC1}-P_{GPD1}-VioE-T_{CYC1}	This study
p416-$A^{CYT}B^{CYT}E$	p416-P_{CCW12}-VioA- Erg1(441-469)-T_{ENO1}-P_{TEF1}-VioB- Erg1(441-469)-T_{CYC1}-P_{GPD1}-VioE-T_{CYC1}	This study
p416-$A^{CYT}BE^{CYT}$	p416-P_{CCW12}-VioA- Erg1(441-469)-T_{ENO1}-P_{TEF1}-VioB-T_{CYC1}-P_{GPD1}- Tgl1(31-55)-VioE-T_{CYC1}	This study
p416-$A^{CYT}B^{L}E$	p416-P_{CCW12}-VioA- Erg1(441-469)-T_{ENO1}-P_{TEF1}-VioB- Erg1(463-496)-T_{CYC1}-P_{GPD1}-VioE-T_{CYC1}	This study
p416-$A^{CYT}BE^{L}$	p416-P_{CCW12}-VioA- Erg1(441-469)-T_{ENO1}-P_{TEF1}-VioB-T_{CYC1}-P_{GPD1}- Tgl1(9-36)-VioE-T_{CYC1}	This study
p416-$AB^{CYT}E$	p416-P_{CCW12}-VioA-T_{ENO1}-P_{TEF1}-VioB-Erg1(441-469)-T_{CYC1}-P_{GPD1}-VioE-T_{CYC1}	This study
p416-$AB^{CYT}E^{CYT}$	p416-P_{CCW12}-VioA-T_{ENO1}-P_{TEF1}-VioB-Erg1(441-469)-T_{CYC1}-P_{GPD1}- Tgl1(31-55)-VioE-T_{CYC1}	This study
p416-$AB^{CYT}E^{L}$	p416-P_{CCW12}-VioA-T_{ENO1}-P_{TEF1}-VioB- Erg1(441-469)-T_{CYC1}-P_{GPD1}- Tgl1(9-36)-VioE-T_{CYC1}	This study
p416-ABE^{CYT}	p416-P_{CCW12}-VioA-T_{ENO1}-P_{TEF1}-VioB-T_{CYC1}-P_{GPD1}-Tgl1(31-55)-VioE-T_{CYC1}	This study
p416-$A^{L}BE$	p416-P_{CCW12}-VioA- Erg1(463-496)-T_{ENO1}-P_{TEF1}-VioB-T_{CYC1}-P_{GPD1}-VioE-T_{CYC1}	This study
p416-$A^{L}B^{CYT}E$	p416-P_{CCW12}-VioA- Erg1(463-496)-T_{ENO1}-P_{TEF1}-VioB- Erg1(441-469)-T_{CYC1}-P_{GPD1}-VioE-T_{CYC1}	This study
p416-$A^{L}BE^{CYT}$	p416-P_{CCW12}-VioA- Erg1(463-496)-T_{ENO1}-P_{TEF1}-VioB-T_{CYC1}-P_{GPD1}- Tgl1(31-55)-VioE-T_{CYC1}	This study
p416-$A^{L}B^{L}E$	p416-P_{CCW12}-VioA- Erg1(463-496)-T_{ENO1}-P_{TEF1}-VioB- Erg1(463-496)-T_{CYC1}-P_{GPD1}-VioE-T_{CYC1}	This study
p416-$A^{L}BE^{L}$	p416-P_{CCW12}-VioA- Erg1(463-496)-T_{ENO1}-P_{TEF1}-VioB-T_{CYC1}-P_{GPD1}- Tgl1(9-36)-VioE-T_{CYC1}	This study
p416-$AB^{L}E$	p416-P_{CCW12}-VioA-T_{ENO1}-P_{TEF1}-VioB- Erg1(463-496)-T_{CYC1}-P_{GPD1}-VioE-T_{CYC1}	This study
p416-$AB^{L}E^{CYT}$	p416-P_{CCW12}-VioA-T_{ENO1}-P_{TEF1}-VioB- Erg1(463-496)-T_{CYC1}-P_{GPD1}- Tgl1(31-55)-VioE-T_{CYC1}	This study
p416-$AB^{L}E^{L}$	p416-P_{CCW12}-VioA-T_{ENO1}-P_{TEF1}-VioB- Erg1(463-496)-T_{CYC1}-P_{GPD1}- Tgl1(9-36)-VioE-T_{CYC1}	This study
p416-ABE^{L}	p416-P_{CCW12}-VioA-T_{ENO1}-P_{TEF1}-VioB-T_{CYC1}-P_{GPD1}-Tgl1(9-36)-VioE-T_{CYC1}	This study
p416-$A^{CYT}B^{CYT}E^{CYT}$	p416-P_{CCW12}-VioA- Erg1(441-469)-T_{ENO1}-P_{TEF1}-VioB- Erg1(441-469)-T_{CYC1}-P_{GPD1}- Tgl1(31-55)-VioE-T_{CYC1}	This study
p416-$A^{CYT}B^{CYT}E^{L}$	p416-P_{CCW12}-VioA- Erg1(441-469)-T_{ENO1}-P_{TEF1}-VioB- Erg1(441-469)-T_{CYC1}-P_{GPD1}- Tgl1(9-36)-	This study

	VioE-T_{CYC1}	
p416-$A^{CYT}B^{L}E^{CYT}$	p416-P_{CCW12}-VioA- Erg1(441-469)-T_{ENO1}-P_{TEF1}-VioB- Erg1(463-496)-T_{CYC1}-P_{GPD1}- Tgl1(31-55)-VioE-T_{CYC1}	This study
p416-$A^{L}B^{CYT}E^{CYT}$	p416-P_{CCW12}-VioA- Erg1(463-496)-T_{ENO1}-P_{TEF1}-VioB- Erg1(441-469)-T_{CYC1}-P_{GPD1}- Tgl1(31-55)-VioE-T_{CYC1}	This study
p416-$A^{L}B^{L}E^{CYT}$	p416-P_{CCW12}-VioA- Erg1(463-496)-T_{ENO1}-P_{TEF1}-VioB- Erg1(463-496)-T_{CYC1}-P_{GPD1}- Tgl1(31-55)-VioE-T_{CYC1}	This study
p416-$A^{L}B^{CYT}E^{L}$	p416-P_{CCW12}-VioA- Erg1(463-496)-T_{ENO1}-P_{TEF1}-VioB- Erg1(441-469)-T_{CYC1}-P_{GPD1}- Tgl1(9-36)-VioE-T_{CYC1}	This study
p416-$A^{CYT}B^{L}E^{L}$	p416-P_{CCW12}-VioA-Erg1(441-469)-T_{ENO1}-P_{TEF1}-VioB- Erg1(463-496)-T_{CYC1}-P_{GPD1}- Tgl1(9-36)-VioE-T_{CYC1}	This study
p416-$A^{L}B^{L}E^{L}$	p416-P_{CCW12}-VioA- Erg1(463-496)-T_{ENO1}-P_{TEF1}-VioB- Erg1(463-496)-T_{CYC1}-P_{GPD1}- Tgl1(9-36)-VioE-T_{CYC1}	This study
pLM494	Harboring β-carotene biosynthesis genes (*crtE, crtI, crtYB*), CEN/ARS single-copy origin and *URA3* marker	Addgene (*94*)
p416-EBI	p416-P_{GPD1}-crtE-T_{ADH1}-P_{CCW12}-crtB-T_{ENO1}-P_{TEF1}-crtI-T_{CYC1}	This study
p416-E^{CYT}BI	p416-P_{GPD1}-crtE-Erg1(441-469)-T_{ADH1}-P_{CCW12}-crtB-T_{ENO1}-P_{TEF1}-crtI-T_{CYC1}	This study
p416-EB^{CYT}I	p416-P_{GPD1}-crtE-T_{ADH1}-P_{CCW12}-Tgl1(31-55)-crtB-T_{ENO1}-P_{TEF1}-crtI-T_{CYC1}	This study
p416-EBI^{CYT}	p416-P_{GPD1}-crtE-T_{ADH1}-P_{CCW12}-crtB-T_{ENO1}-P_{TEF1}-Tgl1(31-55)-crtI-T_{CYC1}	This study
p416-E^{L}BI	p416-P_{GPD1}-crtE-Erg1(463-496)-T_{ADH1}-P_{CCW12}-crtB-T_{ENO1}-P_{TEF1}-crtI-T_{CYC1}	This study
p416-EB^{L}I	p416-P_{GPD1}-crtE-T_{ADH1}-P_{CCW12}-Tgl1(9-36)-crtB-T_{ENO1}-P_{TEF1}-crtI-T_{CYC1}	This study
p416-EBI^{L}	p416-P_{GPD1}-crtE-T_{ADH1}-P_{CCW12}-crtB-T_{ENO1}-P_{TEF1}-Tgl1(9-36)-crtI-T_{CYC1}	This study
pGADT7-ADH700-yeCherry-pTEF1(ashbya)-yeGFP-DHFR	Harboring GFP and mCherry genes	Addgene (*95*)
1x_a1gRNAOp_pCYC1m_yeBFP2	Harboring BFP gene	Addgene (*96*)
p416-BGC	p416-P_{CCW12}-BFP-T_{ENO1}-P_{TEF1}-GFP-T_{CYC1}-P_{GPD1}-	This study

	mCherry-T_{CYC1}	
p416-BGC^{CYT}	p416-P_{CCW12}-BFP-T_{ENO1}-P_{TEF1}-GFP-T_{CYC1}-P_{GPD1}-Tgl1(31-55)-mCherry-T_{CYC1}	This study
p416-BGC^{L}	p416-P_{CCW12}-BFP-T_{ENO1}-P_{TEF1}-GFP-T_{CYC1}-P_{GPD1}-Tgl1(9-36)-mCherry-T_{CYC1}	This study
p416-B^{CYT}GC^{CYT}	p416-P_{CCW12}-BFP- Erg1(441-469)-T_{ENO1}-P_{TEF1}-GFP-T_{CYC1}-P_{GPD1}- Tgl1(31-55)-mCherry-T_{CYC1}	This study
p416-B^{CYT}GC^{L}	p416-P_{CCW12}-BFP- Erg1(441-469)-T_{ENO1}-P_{TEF1}-GFP-T_{CYC1}-P_{GPD1}-Tgl1(9-36)-mCherry-T_{CYC1}	This study
p416-B^{CYT}G^{CYT}C^{CYT}	p416-P_{CCW12}-BFP- Erg1(441-469)-T_{ENO1}-P_{TEF1}-GFP-Erg1(441-469)-T_{CYC1}-P_{GPD1}- Tgl1(31-55)-mCherry-T_{CYC1}	This study
p416-B^{CYT}G^{CYT}C^{L}	p416-P_{CCW12}-BFP- Erg1(441-469)-T_{ENO1}-P_{TEF1}-GFP-Erg1(441-469)-T_{CYC1}-P_{GPD1}- Tgl1(9-36)-mCherry-T_{CYC1}	This study
p416-VioE-6xHis	p416-P_{GPD1}-VioE-6xHis-T_{CYC1}	This study
p416-VioE^{CYT}-6xHis	p416-P_{GPD1}-Tgl1(31-55)-VioE-6xHis-T_{CYC1}	This study
p416-VioE^{CYT-H}-6xHis	p416-P_{GPD1}-Tgl1(37-55)-VioE-6xHis-T_{CYC1}	This study
p416-VioE^{L}-6xHis	p416-P_{GPD1}-Tgl1(9-36)-VioE-6xHis-T_{CYC1}	This study
p416-crtE-6xHis	p416-P_{GPD1}-crtE-6xHis-T_{CYC1}	This study
p416-crtE^{CYT}-6xHis	p416-P_{GPD1}-crtE-Erg1(441-469)-6xHis-T_{CYC1}	This study
p416-crtE^{CYT-H}-6xHis	p416-P_{GPD1}-crtE-Erg1(441-462)-6xHis-T_{CYC1}	This study
p416-crtE^{L}-6xHis	p416-P_{GPD1}-crtE-Erg1(463-496)-6xHis-T_{CYC1}	This study

Table S17. List of strains used in this study.

Strains	Genotype	Reference
CEN.PK2-1D (WT)	*MATα ura3-52 trp1-289 leu2-3,112 his3Δ1 MAL2-8^{C} SUC2*	Euroscarf
LD	CEN.PK2-1D *ΔTGL3 ΔTGL4 ΔHIS3::P_{GPD1}-LRO1*	(*9*)
Eht1-GFP	CEN.PK2-1D [p416-P_{TEF1}-Eht1-GFP]	This study
Erg6-GFP	CEN.PK2-1D [p416-P_{TEF1}-Erg6-GFP]	This study
Tgl3-GFP	CEN.PK2-1D [p416-P_{TEF1}-Tgl3-GFP]	This study
Ayr1-GFP	CEN.PK2-1D [p416-P_{TEF1}-Ayr1-GFP]	This study
Pgc1-GFP	CEN.PK2-1D [p416-P_{TEF1}-Pgc1-GFP]	This study
Hfd1-GFP	CEN.PK2-1D [p416-P_{TEF1}-Hfd1-GFP]	This study
Erg1-GFP	CEN.PK2-1D [p416-P_{TEF1}-Erg1-GFP]	This study
Fat1-GFP	CEN.PK2-1D [p416-P_{TEF1}-Fat1-GFP]	This study
Loa1-GFP	CEN.PK2-1D [p416-P_{TEF1}-Loa1-GFP]	This study
Slc1-GFP	CEN.PK2-1D [p416-P_{TEF1}-Slc1-GFP]	This study
Tgl1-GFP	CEN.PK2-1D [p416-P_{TEF1}-Tgl1-GFP]	This study
Erg1-GFP	CEN.PK2-1D [p416-P_{TEF1}-Erg1-GFP]	This study
Erg1^{CYT}-GFP	CEN.PK2-1D [p416-P_{TEF1}-Erg1^{CYT}-GFP]	This study
Erg1^{CYT-H}-GFP	CEN.PK2-1D [p416-P_{TEF1}-Erg1^{CYT-H}-GFP]	This study
Erg1^{L}-GFP	CEN.PK2-1D [p416-P_{TEF1}-Erg1^{L}-GFP]	This study
Erg1^{L-H}-GFP	CEN.PK2-1D [p416-P_{TEF1}-Erg1^{L-H}-GFP]	This study
Tgl1^{CYT}-GFP	CEN.PK2-1D [p416-P_{TEF1}-Tgl1^{CYT}-GFP]	This study
Tgl1^{CYT-H}-GFP	CEN.PK2-1D [p416-P_{TEF1}-Tgl1^{CYT-H}-GFP]	This study
Tgl1^{L}-GFP	CEN.PK2-1D [p416-P_{TEF1}-Tgl1^{L}-GFP]	This study
Tgl1^{L-H}-GFP	CEN.PK2-1D [p416-P_{TEF1}-Tgl1^{L-H}-GFP]	This study
AB	LD [p416-AB]	This study
ABE	LD [p416-ABE]	This study
A^{CYT}BE	LD [p416-A^{CYT}BE]	This study
A^{CYT}B^{CYT}E	LD [p416-A^{CYT}B^{CYT}E]	This study
A^{CYT}BE^{CYT}	LD [p416-A^{CYT}BE^{CYT}]	This study
A^{CYT}B^{L}E	LD [p416-A^{CYT}B^{L}E]	This study
A^{CYT}BE^{L}	LD [p416-A^{CYT}BE^{L}]	This study

$AB^{CYT}E$	LD [p416-$AB^{CYT}E$]	This study
$AB^{CYT}E^{CYT}$	LD [p416-$AB^{CYT}E^{CYT}$]	This study
$AB^{CYT}E^{L}$	LD [p416-$AB^{CYT}E^{L}$]	This study
ABE^{CYT}	LD [p416-ABE^{CYT}]	This study
$A^{L}BE$	LD [p416-$A^{L}BE$]	This study
$A^{L}B^{CYT}E$	LD [p416-$A^{L}B^{CYT}E$]	This study
$A^{L}BE^{CYT}$	LD [p416-$A^{L}BE^{CYT}$]	This study
$A^{L}B^{L}E$	LD [p416-$A^{L}B^{L}E$]	This study
$A^{L}BE^{L}$	LD [p416-$A^{L}BE^{L}$]	This study
$AB^{L}E$	LD [p416-$AB^{L}E$]	This study
$AB^{L}E^{CYT}$	LD [p416-$AB^{L}E^{CYT}$]	This study
$AB^{L}E^{L}$	LD [p416-$AB^{L}E^{L}$]	This study
ABE^{L}	LD [p416-ABE^{L}]	This study
$A^{CYT}B^{CYT}E^{CYT}$	LD [p416-$A^{CYT}B^{CYT}E^{CYT}$]	This study
$A^{CYT}B^{CYT}E^{L}$	LD [p416-$A^{CYT}B^{CYT}E^{L}$]	This study
$A^{CYT}B^{L}E^{CYT}$	LD [p416-$A^{CYT}B^{L}E^{CYT}$]	This study
$A^{L}B^{CYT}E^{CYT}$	LD [p416-$A^{L}B^{CYT}E^{CYT}$]	This study
$A^{L}B^{L}E^{CYT}$	LD [p416-$A^{L}B^{L}E^{CYT}$]	This study
$A^{L}B^{CYT}E^{L}$	LD [p416-$A^{L}B^{CYT}E^{L}$]	This study
$A^{CYT}B^{L}E^{L}$	LD [p416-$A^{CYT}B^{L}E^{L}$]	This study
$A^{L}B^{L}E^{L}$	LD [p416-$A^{L}B^{L}E^{L}$]	This study
EBI	LD [p416-EBI]	This study
$E^{CYT}BI$	LD [p416-$E^{CYT}BI$]	This study
$EB^{CYT}I$	LD [p416-$EB^{CYT}I$]	This study
EBI^{CYT}	LD [p416-$EBI^{CYT]}$	This study
$E^{L}BI$	LD [p416-$E^{L}BI$]	This study
$EB^{L}I$	LD [p416-$EB^{L}I$]	This study
EBI^{L}	LD [p416-EBI^{L}]	This study
BGC	LD [p416-BGC]	This study
BGC^{CYT}	LD [p416-BGC^{CYT}]	This study
BGC^{L}	LD [p416-BGC^{L}]	This study

$B^{CYT}GC^{CYT}$	LD [p416-$B^{CYT}GC^{CYT}$]	This study
$B^{CYT}GC^{L}$	LD [p416-$B^{CYT}GC^{L}$]	This study
$B^{CYT}G^{CYT}C^{CYT}$	LD [p416-$B^{CYT}G^{CYT}C^{CYT}$]	This study
$B^{CYT}G^{CYT}C^{L}$	LD [p416-$B^{CYT}G^{CYT}C^{L}$]	This study
ABE	LD [p416-ABE]	This study
$A^{CYT}BE$	LD [p416-$A^{CYT}BE$]	This study
$A^{CYT}B^{CYT}E$	LD [p416-$A^{CYT}B^{CYT}E$]	This study
$A^{CYT}BE^{CYT}$	LD [p416-$A^{CYT}BE^{CYT}$]	This study
$A^{CYT}B^{L}E$	LD [p416-$A^{CYT}B^{L}E$]	This study
$A^{CYT}BE^{L}$	LD [p416-$A^{CYT}BE^{L}$]	This study
$AB^{CYT}E$	LD [p416-$AB^{CYT}E$]	This study
$AB^{CYT}E^{CYT}$	LD [p416-$AB^{CYT}E^{CYT}$]	This study
$AB^{CYT}E^{L}$	LD [p416-$AB^{CYT}E^{L}$]	This study
ABE^{CYT}	LD [p416-ABE^{CYT}]	This study
$A^{L}BE$	LD [p416-$A^{L}BE$]	This study
$A^{L}B^{CYT}E$	LD [p416-$A^{L}B^{CYT}E$]	This study
$A^{L}BE^{CYT}$	LD [p416-$A^{L}BE^{CYT}$]	This study
$A^{L}B^{L}E$	LD [p416-$A^{L}B^{L}E$]	This study
$A^{L}BE^{L}$	LD [p416-$A^{L}BE^{L}$]	This study
$AB^{L}E$	LD [p416-$AB^{L}E$]	This study
$AB^{L}E^{CYT}$	LD [p416-$AB^{L}E^{CYT}$]	This study
$AB^{L}E^{L}$	LD [p416-$AB^{L}E^{L}$]	This study
ABE^{L}	LD [p416-ABE^{L}]	This study
$A^{CYT}B^{CYT}E^{CYT}$	LD [p416-$A^{CYT}B^{CYT}E^{CYT}$]	This study
$A^{CYT}B^{CYT}E^{L}$	LD [p416-$A^{CYT}B^{CYT}E^{L}$]	This study
$A^{CYT}B^{L}E^{CYT}$	LD [p416-$A^{CYT}B^{L}E^{CYT}$]	This study
$A^{L}B^{CYT}E^{CYT}$	LD [p416-$A^{L}B^{CYT}E^{CYT}$]	This study
$A^{L}B^{L}E^{CYT}$	LD [p416-$A^{L}B^{L}E^{CYT}$]	This study
$A^{L}B^{CYT}E^{L}$	LD [p416-$A^{L}B^{CYT}E^{L}$]	This study
$A^{CYT}B^{L}E^{L}$	LD [p416-$A^{CYT}B^{L}E^{L}$]	This study
$A^{L}B^{L}E^{L}$	LD [p416-$A^{L}B^{L}E^{L}$]	This study

VioE	LD[p416-VioE-6xHis]	This study
$VioE^{CYT}$	LD[p416-$VioE^{CYT}$-6xHis]	This study
$VioE^{CYT\text{-}H}$	LD[p416-$VioE^{CYT\text{-}H}$-6xHis]	This study
$VioE^{L}$	LD[p416-$VioE^{L}$-6xHis]	This study
crtE	LD[p416-crtE-6xHis]	This study
$crtE^{CYT}$	LD[p416-$crtE^{CYT}$-6xHis]	This study
$crtE^{CYT\text{-}H}$	LD[p416-$crtE^{CYT\text{-}H}$-6xHis]	This study
$crtE^{L}$	LD[p416-$crtE^{L}$-6xHis]	This study

Table S18. List of primers in this study.

Primer name	Primer sequence (5'-3')
TEF-F	CAAAAGCTGGAGCTCATAGCTTCAAAATGTTTCTACTCCTTTTTTAC
TEF-R	CTAGTTCTAGAAAACTTAGATTAGATTGCTATGCTTTCTTTCTAATG
Eht1-N-LD-F	GTTTTCTAGAACTAGTATGTCAGAAGTTTCCAAATG
Eht1-N-LD-R	TTCACCTTTAGATGAACCGGATCCGAATGGGTTGATTGCTGGCCATTTGGAAACTTCT
Eht1-C-LD-F	CCATGGTATGGATGAATTGTACAAAGGATCCGGTTCAAAAGACAACAACTCGTGGG
Eht1-C-LD-R	ATCCGGGGTCTCGAGTCATACGACTAATTCATCAAACTTAGTGAAAAAATT
ERG6-LD-F	GTTTTCTAGAACTAGTATGGTAATGGAAAAATTAGGTCTAG
ERG6-LD-R	ACCAGTGAATAATTCTTCACCTTTAGATGAACCGGATCCTGGAGTGAATAACTTGGACTTACC
ERG6-GFP-F	GTAAGTCCAAGTTATTCACTCCAGGATCCGGTTCATCTAAAGGTGAAGAATTATTCACTGG
FAT1-LD-F	TTTTCTAGAACTAGTATGTCTCCCATACAG
FAT1-LD-R	GAATAATTCTTCACCTTTAGATGAACCGGATCCCTGGATAGGGGTTATAATTAGCTTGA
FAT1-GFP-F	TAATTATAACCCCTATCCAGGGATCCGGTTCATCTAAAGGTGAAGAATTATTCACTGGT
TGL1-LD-F	TCTAAGTTTTCTAGAACTAGTATGTTATCGATAACAGATTACATTATAGTCGTCT
TGL1-LD-R	ATAATTCTTCACCTTTAGATGAACCGGATCCGGTATTGTCATCGGAGGACGTTGAGAAAT
TGL1-GFP-F	CCTCCGATGACAATACCGGATCCGGTTCATCTAAAGGTGAAGAATTATTCACTGG
PGC1-LD-F	TAAGTTTTCTAGAACTAGTATGTGGGTCCATATTAAGTTGTGC
PGC1-LD-R	TGAATAATTCTTCACCTTTAGATGAACCGGATCCAAGGAACAAAAAAATCACATAAGCAAT
PGC1-GFP-F	TGATTTTTTTGTTCCTTGGATCCGGTTCATCTAAAGGTGAAGA

	ATTATTCACTGG
AYR1-LD-F	GTTTTCTAGAACTAGTATGGTTTATAGAGGAACGTTCGCCA
AYR1-LD-R	ATTCTTCACCTTTAGATGAACCGGATCCCAATAGCCAGTAAGG AACA
AYR1-GFP-F	CTTACTGGCTATTGGGATCCGGTTCATCTAAAGGTGAAGAATT ATT
SLC1-LD-F	GTTTTCTAGAACTAGTATGAGTGTGATAGGTAGGTTC
SLC1-LD-R	CCAGTGAATAATTCTTCACCTTTAGATGAACCGGATCCGGCGA TTACACCGTAAAAG
SLC1-GFP-F	CTTTTACGGTGTAATCGCCGGATCCGGTTCATCTAAAGGTGAA GAATTATTCACTGG
TGL3-LD-F	GTTTTCTAGAACTAGTATGTTGTCACTGGGCACAACAG
TGL3-LD-R	CTTCACCTTTAGATGAACCGGATCCCACCCCAAGGTGAAACA GCCC
TGL3-GFP-F	TCACCTTGGGGTGGGATCCGGTTCATCTAAAGGTGAAGAATT ATTCACTGG
LOA1-LD-F	GTTTTCTAGAACTAGTATGGCGTGTTTGTTGGGTATCC
LOA1-LD-R	TCTTCACCTTTAGATGAACCGGATCCTAGAAGGTACAGCATAA TCAATGGTAGC
LOA1-GFP-F	GCTGTACCTTCTAGGATCCGGTTCATCTAAAGGTGAAGAATTA TTCACTGG
ERG1-LD-F	TTTTCTAGAACTAGTATGCCTTTGCAATTGACCAGGGTTT
ERG1-LD-R	ATAATTCTTCACCTTTAGATGAACCGGATCCACCAATCAACTC ACCAAACAAAAATGGG
ERG1-GFP-F	CCCATTTTTGTTTGGTGAGTTGATTGGTGGATCCGGTTCATCT AAAGGTGAAGAATTATTCACTGG
HFD1-LD-F	GTTTTCTAGAACTAGTATGGGCAGTGTCTTGATTATTGCTCC
HFD1-LD-R	CTTCACCTTTAGATGAACCGGATCCTGCAGCAAGAGCTGCTG CCAATGGGGC
HFD1-GFP-F	CAGCTCTTGCTGCAGGATCCGGTTCATCTAAAGGTGAAGAAT TATTCACTGG
GFP R	GGGATCCACTAGTTCATTTGTACAATTCATCCATACCATGGG

Erg1-CYT-F	CATGGTATGGATGAATTGTACAAAGGATCCGGATCCC CTTTGCAATTGACCAGGGTTTT
Erg1-CYT-R	GATCCGGGGTCTCGAGTTATGGTAATCCCAAGAAACCACGTT CTT
GFP-Erg1-F	GTTTTCTAGAACTAGTATGTCTAAAGGTGAAGAATTATTCACT GGTG
GFP-Erg1-CYT-R	GCGACAGCGAAGAAAACCCTGGTCAATTGCAAAGGGGATCC GGATCCTTTGTACAATTCATCCATACCATGGGTAATACCAGC
Erg1-L-F	TACCCATGGTATGGATGAATTGTACAAAGGATCCGGATCCCGT GGTTTCTTGGGATTACCAAT
Erg1-L-R	GGATCCGGGGTCTCGAGTTAACCAATCAACTCACCAAACAAA AATGGGGT
GFP-Erg1-L-R	TCCAATAAAGCCATTGGTAATCCCAAGAAACCACGGATCCG GATCCTTTGTACAATTCATCCATACCATGGGTAATACCAGC
Erg1-CYT-F	CATGGTATGGATGAATTGTACAAAGGATCCGGATCCCCTTTGC AATTGACCAGGGTTTT
Erg1-CYT-H-R	GGGGATCCGGGGTCTCGAGTTATTCTTCCATGTTCAAGTAAAT GG
GFP-Erg1-CYT-R	GCGACAGCGAAGAAAACCCTGGTCAATTGCAAAGGGGATCC GGATCCTTTGTACAATTCATCCATACCATGGGTAATACCAGC
Erg1-L-H-F	ACCCATGGTATGGATGAATTGTACAAAGGATCCGGATCCATGG CTTTATTGGAAGGTATTAT
GFP-Erg1-L-H-R	TCCAATAAAGCCATGGATCCGGATCCTTTGTACAATTCATCCA TACCATGGGTAATACCAGC
Tgl1-CYT-F	TCTAAGTTTTCTAGAACTAGTATGAAACTTATACCACAACCAA TGATTAA
Tgl1-CYT-R	TGAATAATTCTTCACCTTTAGAGGATCCGGATCCGGTATTGTC ATCGGAGGACGT
GFP-Tgl1-CYT-F	ATGACAATACCGGATCCGGATCCTCTAAAGGTGAAGAATTATT CACTGG
GFP-Tgl1-R	CGGGGGATCCGGGGTCTCGAGTTATTTGTACAATTCATCCATA CCATGGGTAATACCAG
Tgl1-L-F	TAATCTAAGTTTTCTAGAACTAGTATGTTATCGATAACAGATTA CATTATAG
Tgl1-L-R	TGAATAATTCTTCACCTTTAGAGGATCCGGATCCTGGTTGTGG

	TATAAGTTTAAGGACT
GFP-Tgl1-L-F	ATACCACAACCAGGATCCGGATCCTCTAAAGGTGAAGAATTATTCACTGG
Tgl1-CYT-H-F	AGTTTTCTAGAACTAGTATGATGATTAACCTTTTCGAATGGCT
Tgl1-CYT-R	TGAATAATTCTTCACCTTTAGAGGATCCGGATCCGGTATTGTCATCGGAGGACGT
GFP-Tgl1-CYT-F	ATGACAATACCGGATCCGGATCCTCTAAAGGTGAAGAATTATTCACTGG
Tgl1-L-F	TAATCTAAGTTTTCTAGAACTAGTATGTTATCGATAACAGATTACATTATAG
Tgl1-L-H-R	AGTGAATAATTCTTCACCTTTAGAGGATCCGGATCCAAGGACTGATGAAATAATGCTTT
GFP-Tgl1-L-H-F	TATTTCATCAGTCCTTGGATCCGGATCCTCTAAAGGTGAAGAATTATTCACTGG
CCW12 F	GATAATGAGAGCTCTTCGCGGCCACCTACGCC
CCW12-VioA R	CATACTAGTTCTAGATATTGATATAGTGTTTAAGCGAATGACAGAAG
VioA F	TCTAGAACTAGTATGAAACATTCTTCCGATATCTGCATTGTTG
VioA R	CAAAAGCTGAATTCCTTACGCGGCGATACGCTGC
VioA-ENO1t F	GGAATTCAGCTTTTGATTAAGCCTTCTAGTCCAAAAAAC
VioA-ENO1t R	ATCTGGTTTGGCAGCATACATGGGTGACCAAAAGAGCG
VioA-Erg1-CYT-F	GTCTGCTGTTGCAGCGTATCGCCGCGGGATCCGGATCCCCTTTGCAATTGACCAGGGTTT
Erg1-CYT-VioA-R	AGAAAACCCTGGTCAATTGCAAAGGGGATCCGGATCCCGCGGCGATACGCTG
Erg1-CYT-tENO1-R	AGAAGGCTTAATCAAAAGCTGAATTCCTTATGGTAATCCCAAGAAACCACGTTCTTC
VioA-Erg1-L-F	TCTGCTGTTGCAGCGTATCGCCGCGGGATCCGGATCCCGTGGTTTCTTGGGATTAC
Erg1-L-VioA-R	TAAAGCCATTGGTAATCCCAAGAAACCACGGGATCCGGATCCCGCGGCGATACGCTGCAA
Erg1-L-tENO1-R	TAATCAAAAGCTGAATTCCTTAACCAATCAACTCACCAAACAAAAATG

VioB-TEF1 F	GCTGCCAAACCAGATGTCAACACAGCTACAACGCCTTG
VioB-TEF1 R	CTTTTGTAATTAAAACTTAGATTAGATTGCTATGCTTTCTTTCTAATG
VioB F	TTTTAATTACAAAAGATCTATGAGCATTCTGGATTTCCCGCG
VioB R	CTTGATATCGAATTCTTAGGCCTCGCGGCTCAGTTTG
VioB-CYC1t F	GAATTCGATATCAAGCTTATCGATACCGTCGACCTCGAGTCATGTAATTAGTTATGTCACGCTTACATTCAC
VioB-CYC1t R	GAATTGGGTACCGGCCGCAAATTAAAGCCTTCGAGCGTCCC
Erg1-CYT-VioB-R	ACCCTGGTCAATTGCAAAGGGGATCCGGATCCGGCCTCGCGGCTCAGTTTGC
VioB-Erg1-CYT-F	AACTGAGCCGCGAGGCCGGATCCGGATCCCCTTTGCAATTGACCAGGGTTT
Erg1-CYT-tCYC1-R	CTTGATATCGAATTCTTATGGTAATCCCAAGAAACCACGTTC
Erg1-L-VioB-R	CAAGAAACCACGGGATCCGGATCCGGCCTCGCGGCTCAGTTTGC
VioB-Erg1-L-F	ACTGAGCCGCGAGGCCGGATCCGGATCCCGTGGTTTCTTGGGATTACCAATG
Erg1-L-tCYC1-R	CTTGATATCGAATTCTTAACCAATCAACTCACCAAACAAAAATGG
VioE-GPD1 F	ACTAGTTCTAGAATCCGTCGAAACTAAGTTCTGGTGTTTTAAAACTAAAAAAAAG
VioE-GPD1 R	TGGCCGCGAAGAGCTCTCATTATCAATACTCGCCATTTCAAAGAATACGTAAATAATTAATAG
VioE F	CGGATTCTAGAACTAGTATGGAGAACCGTGAGCCAC
VioE R	CTTGATATCGAATTCTTAGCGCTTGGCCGCGAAAAC
VioE-CYC1t F	GAATTCGATATCAAGCTTATCGATACCGTCGACCTCGAGTCATG
VioE-CYC1t R	ACAAAAGCTGGAGCTCGCAAATTAAAGCCTTCGAGCGTCCC
Tgl1-CYT-GPDp-F	CGGATTCTAGAACTAGTATGAAACTTATACCACAACCA
VioE-Tgl1-CYT-R	GTGGCTCACGGTTCTCTGAACCGGATCCGGTATTGTCATC

Tgl1-CYT-VioE-F	ACAATACCGGATCCGGTTCAGAGAACCGTGAGCCACCAC
Tgl1-L-GPDp-F	CGGATTCTAGAACTAGTATGTTATCGATAACAGATTACATTATAGTCG
VioE-Tgl1-L-R	TGGCTCACGGTTCTCTGAACCGGATCCTGGTTGTGGTATAAGTTTA
Tgl1-L-VioE-F	TTATACCACAACCAGGATCCGGTTCAGAGAACCGTGAGCCACCACTGT
crtE-F	CGACGGATTCTAGAACTAGTACCATGGATTACGCGAACATCCTCACAG
crtE-R	CATAAGAAATTCGCCCCGGGTCACAGAGGGATATCGGCTAGCTTTTTCAG
Erg1-CYT-crtE-R	CATAAGAAATTCGCCCCGGGTCATGGTAATCCCAAGAAACCACGTTCTTCCATGTTCAAGTAAATGGTGTAAAAAGCGACAGCGAAGAAAACCCTGGTCAATTGCAAAGGTGATCCACCCCCGCC
Erg1-L-crtE-R	CATAAGAAATTCGCCCCGGGTCAAAACAAAAATGGGGTGAATACTCTAATAGCTGTGATCAAAATCATAATACCTTCCAATAAAGCCATTGGTAATCCCAAGAAACCACGTGATCCACCCCCGCC
linker-crtE-R	TGATCCACCCCCGCCGGAACCCCCGCCTCCAGACCCGCCTCCACCCAGAGGGATATCGGCTAGCTTTTTCAG
ADH1t-crtE-F	CCCGGGGCGAATTTCTTATGATTTATGATTTTTATTATTAAATAAGTTATAAAAAAAATAAGTG
ADH1t-crtE-R	ATTTTGAAGCTATGGGATCCCATGCCGGTAGAGGTGTGGTC
CCW12p-crtB-F	GATAATGATAAACTGAGCTCTTCGCGGCCACCTACGC
CCW12p-Tgl1-CYT-R	TCATTGGTTGTGGTATAAGTTTCATGGTTATTGATATAGTGTTTAAGCGAATGACAGAAGATTAATTTCTTG
CCW12p-Tgl1-L-R	AGACGACTATAATGTAATCTGTCATGGTTATTGATATAGTGTTTAAGCGAATGACAGAAGATTAATTTCTTG
CCW12p-crtB-R	TATGGTCGGCTGCTCATGGTTATTGATATAGTGTTTAAGCGAATGACAGAAGATTAATTTC
crtB-F	ACCATGAGCAGCCGACCATACTCTTCTC
Tgl1-CYT-crtB-F	ACCATGAAACTTATACCACAACCAATGATTAACCTTTTCGAATGGCTAATAAATTTCTCAACGTCCTCCGATGACAATACCCGAACTATTTATGGCAACAAAAAGATGCC
Tgl1-L-crtB-F	ACCATGACAGATTACATTATAGTCGTCTTGGTATACATAGAAAGCATTATTTCATCAGTCCTTAAACTTATACCACAACCACGAAC

	TATTTATGGCAACAAAAAGATGCC
linker-crtB-F	CGAACTATTTATGGCAACAAAAAGATGCCATCTTCATTTCCCCTCATTACACCGCCTGTGCTCTCCCTGTTTTTAGCAGCCGACCATACTCTTCTC
crtB-R	ATCAAAAGCTTCACTGCCCTTCCCATCCGC
ENO1t-crtB-F	GAAGGGCAGTGAAGCTTTTGATTAAGCCTTCTAGTCCAAAAAAC
ENO1t-crtB-R	GGGAACAAAAGCTGGAGCTCATACATGGGTGACCAAAAGAGCG
TEF1p-crtI-F	GGATCCCATAGCTTCAAAATGTTTCTACTCCTTTTTTACTCTTCC
TEF1p-crtI-R	TGATCTTGTTCTTTTCCCATGGTCTTAGATTAGATTGCTATGCTTTCTTTCTAATGAGCAAG
TEF1p-Tgl1-CYT-R	GGTTGTGGTATAAGTTTCATGGTCTTAGATTAGATTGCTATGCTTTCTTTCTAATGAGCAAG
TEF1p-Tgl1-L-R	ACTATAATGTAATCTGTCATGGTCTTAGATTAGATTGCTATGCTTTCTTTCTAATGAGCAAG
crtI-F	ACCATGGGAAAAGAACAAGATCAGGATAAACCC
Tgl1-CYT-crtI-F	GCATAGCAATCTAATCTAAGACCATGAAACTTATACCACAACCAATGATTAACCTTTTCGAATGGCTAATAAATTTCTCAACGTCCTCCGATGACAATACCGAAGCTGCCGCAAAAGAGGC
Tgl1-L-crtI-F	GCATAGCAATCTAATCTAAGACCATGACAGATTACATTATAGTCGTCTTGGTATACATAGAAAGCATTATTTCATCAGTCCTTAAACTTATACCACAACCAGAAGCTGCCGCAAAAGAGGC
linker-crtI-F	GAAGCTGCCGCAAAAGAGGCCGCTGCGAAGGAAGCAGCCGCTAAAGAGGCGGCAGCCAAGGGAAAAGAACAAGATCAGGATAAACCCACAGCTA
crtI-R	ATAAGCTTGATATCGAATTCTTATCTCTCCAAATCCATGAACTTCAGCTGG
BFP F	TATCAATATCTAGAACTAGTATGGTTTCCAAGGGTGAAGAATTATTCACC
BFP R	TTAATCAAAAGCTGAATTCCTTACTTGTACAATTCGTCCATACCCAAAGTG
BFP-Erg1-CYT-F	CACTTTGGGTATGGACGAATTGTACAAGGGATCCGGATCCCCTTTGC

Erg1-CYT-BFP-R	AATTGCAAAGGGGATCCGGATCCCTTGTACAATTCGTCCATACCCAAAGTG
GFP F	TTTTAATTACAAAAGATCTATGTCTAAAGGTGAAGAATTATTCACTGG
GFP R	ATAAGCTTGATATCGAATTCTTATTTGTACAATTCATCCATACCATGGGTAATACCAG
GFP-Erg1-CYT-F	ATTACCCATGGTATGGATGAATTGTACAAAGGATCCGGATCCCCTTTG
Erg1-CYT-GFP-R	GTCAATTGCAAAGGGGATCCGGATCCTTTGTACAATTCATCCATACCATGGGTAATACCAG
mCherry F	CGACGGATTCTAGAACTAGTATGGTAAGTAAGGGTGAAGAAGACAATATGGCG
mCherry R	ATAAGCTTGATATCGAATTCTTATTTGTACAATTCGTCCATTCCACCCG
mCherry-Tgl1-CYT-R	TTCTTCACCCTTACTTACTGAACCGGATCCGGTATTGTCATC
Tgl1-CYT-mCherry-F	CGATGACAATACCGGATCCGGTTCAGTAAGTAAGGGTGAAGAAGACAATATGGCG
mCherry-Tgl1-L-R	CTTCACCCTTACTTACTGAACCGGATCCTGGTTGTGGTATAAGTTTA
Tgl1-L-mCherry-F	TATACCACAACCAGGATCCGGTTCAGTAAGTAAGGGTGAAGAAGACAATATGGCG
VioE-SpeI-F	CGACGGATTCTAGAACTAGTATGGAGAACCGTGAGCCACCACT
6His-VioE-EcoRI-R	ATAAGCTTGATATCGAATTCTTAGTGGTGATGATGGTGATGGCGCTTGGCCGCGAAAAC
VioE-Tgl1-CYT-SpeI-F	CGGATTCTAGAACTAGTATGAAACTTATACCACAACCAATGATT
VioE-Tgl1-CYT-H-SpeI-F	CGGATTCTAGAACTAGTATGATGATTAACCTTTTCGAATGGCTAA
VioE-Tgl1-LD-SpeI-F	CGACGGATTCTAGAACTAGTATGTTATCGATAACA
crtE-6His-SpeI-F	CGACGGATTCTAGAACTAGTATGCATCACCATCATCACCACGATTACGCGAACATCCTCACAG
crtE-EcoRI-R	ATAAGCTTGATATCGAATTCTTACAGAGGGATATCGGCTAGCT

	TTTTCAG
ERG1-CYT-EcoRI-R	ATAAGCTTGATATCGAATTCTTATGGTAATCCCAAGAAACCACGTTC
ERG1-CYT-H-EcoRI-R	ATAAGCTTGATATCGAATTCTTATTCTTCCATGTTCAAGTAAATGGTGTAAAAAGCG
ERG1-LD-EcoRI-R	ATAAGCTTGATATCGAATTCTTAACCAATCAACTCACCAAACAAAAATGGGGTGA

Movie S1. Time-lapse fluorescence microscopy of the cytosol-facing GFP (Tgl1^{CYT}-GFP) after proteinase K treatment.

Movie S2. Time-lapse fluorescence microscopy of the LD inner space-facing GFP (Tgl1^{L}-GFP) after proteinase K treatment.

REFERENCES

1. S. Y. Moon, S.-H. Son, S. S. Oh, J. Y. Lee, Harnessing cellular organelles to bring new functionalities into yeast. *Biotechnol. Bioprocess Eng.* **28**, 936–948 (2023).

2. S. K. Hammer, J. L. Avalos, Harnessing yeast organelles for metabolic engineering. *Nat. Chem. Biol.* **13**, 823–832 (2017).

3. H. J. Wagner, C. C. Capitain, K. Richter, M. Nessling, J. Mampel, Engineering bacterial microcompartments with heterologous enzyme cargos. *Eng. Life Sci.* **17**, 36–46 (2017).

4. J. Liu, F. Zhorabek, Y. Chau, Biomaterial design inspired by membraneless organelles. *Matter* **5**, 2787–2812 (2022).

5. D. Yang, H. Eun, C. P. S. Prabowo, S. Cho, S. Y. Lee, Metabolic and cellular engineering for the production of natural products. *Curr. Opin. Biotechnol.* **77**, 102760 (2022).

6. J. S. Cho, G. B. Kim, H. Eun, C. W. Moon, S. Y. Lee, Designing microbial cell factories for the production of chemicals. *JACS Au* **2**, 1781–1799 (2022).

7. R. Dhiman, S. Caesar, A. R. Thiam, B. Schrul, Mechanisms of protein targeting to lipid droplets: A unified cell biological and biophysical perspective. *Semin. Cell Dev. Biol.* **108**, 4–13 (2020).

8. T. C. Walther, R. V. Farese Jr., Lipid droplets and cellular lipid metabolism. *Annu. Rev. Biochem.* **81**, 687–714 (2012).

9. S. H. Son, G. Park, J. Lim, C. Y. Son, S. S. Oh, J. Y. Lee, Chain flexibility of medicinal lipids determines their selective partitioning into lipid droplets. *Nat. Commun.* **13**, 3612 (2022).

10. T. Ma, B. Shi, Z. Ye, X. Li, M. Liu, Y. Chen, J. Xia, J. Nielsen, Z. Deng, T. Liu, Lipid engineering combined with systematic metabolic engineering of *Saccharomyces cerevisiae* for high-yield production of lycopene. *Metab. Eng.* **52**, 134–142 (2019).

11. M. Valachovic, M. Garaiova, R. Holic, I. Hapala, Squalene is lipotoxic to yeast cells defective in lipid droplet biogenesis. *Biochem. Biophys. Res. Commun.* **469**, 1123–1128 (2016).

12. X. Bu, J.-Y. Lin, C.-Q. Duan, M. A. G. Koffas, G.-L. Yan, Dual regulation of lipid droplet-triacylglycerol metabolism and ERG9 expression for improved beta-carotene production in *Saccharomyces cerevisiae*. *Microb. Cell Fact.* **21**, 3 (2022).

13. S. Gad, S. Ayakar, Protein scaffolds: A tool for multi-enzyme assembly. *Biotechnol. Rep.* **32**, e00670 (2021).

14. T. C. Walther, J. Chung, R. V. Farese Jr., Lipid droplet biogenesis. *Annu. Rev. Cell Dev. Biol.* **33**, 491–510 (2017).

15. T. Fujimoto, R. G. Parton, Not just fat: The structure and function of the lipid droplet. *Cold Spring Harb. Perspect. Biol.* **3**, a004838 (2011).

16. M. Poppelreuther, S. Sander, F. Minden, M. S. Dietz, T. Exner, C. Du, I. Zhang, F. Ehehalt, L. Knüppel, S. Domschke, A. Badenhop, S. Staudacher, R. Ehehalt, W. Stremmel, C. Thiele, M. Heilemann, J. Füllekrug, The metabolic capacity of lipid droplet localized acyl-CoA synthetase 3 is not sufficient to support local triglyceride synthesis independent of the endoplasmic reticulum in A431 cells. *Biochim. Biophys. Acta Mol. Cell Biol. Lipids* **1863**, 614–624 (2018).

17. F. Wilfling, H. Wang, J. T. Haas, N. Krahmer, T. J. Gould, A. Uchida, J.-X. Cheng, M. Graham, R. Christiano, F. Fröhlich, X. Liu, K. K. Buhman, R. A. Coleman, J. Bewersdorf, R. V. Farese Jr., T. C. Walther, Triacylglycerol synthesis enzymes mediate lipid droplet growth by relocalizing from the ER to lipid droplets. *Dev. Cell* **24**, 384–399 (2013).

18. E. Currie, X. Guo, R. Christiano, C. Chitraju, N. Kory, K. Harrison, J. Haas, T. C. Walther, R. V. Farese Jr., High confidence proteomic analysis of yeast LDs identifies additional droplet proteins and reveals connections to dolichol synthesis and sterol acetylation. *J. Lipid Res.* **55**, 1465–1477 (2014).

19. K. Bersuker, J. A. Olzmann, Establishing the lipid droplet proteome: Mechanisms of lipid droplet protein targeting and degradation. *Biochim. Biophys. Acta Mol. Cell Biol. Lipids* **1862**, 1166–1177 (2017).

20. I. Bouchez, M. Pouteaux, M. Canonge, M. Genet, T. Chardot, A. Guillot, M. Froissard, Regulation of lipid droplet dynamics in *Saccharomyces cerevisiae* depends on the Rab7-like Ypt7p, HOPS complex and V1-ATPase. *Biol. Open* **4**, 764–775 (2015).

21. L. Käll, A. Krogh, E. L. L. Sonnhammer, A combined transmembrane topology and signal peptide prediction method. *J. Mol. Biol.* **338**, 1027–1036 (2004).

22. A. Krogh, B. Larsson, G. von Heijne, E. L. Sonnhammer, Predicting transmembrane protein topology with a hidden Markov model: Application to complete genomes. *J. Mol. Biol.* **305**, 567–580 (2001).

23. J. J. Almagro Armenteros, K. D. Tsirigos, C. K. Sonderby, T. N. Petersen, O. Winther, S. Brunak, G. von Heijne, H. Nielsen, SignalP 5.0 improves signal peptide predictions using deep neural networks. *Nat. Biotechnol.* **37**, 420–423 (2019).

24. J. Zhu, C. Schwartz, I. Wheeldon, Controlled intracellular trafficking alleviates an expression bottleneck in *S. cerevisiae* ester biosynthesis. *Metab. Eng. Commun.* **8**, e00085 (2019).

25. A. Jandrositz, J. Petschnigg, R. Zimmermann, K. Natter, H. Scholze, A. Hermetter, S. D. Kohlwein, R. Leber, The lipid droplet enzyme Tgl1p hydrolyzes both steryl esters and triglycerides in the yeast, *Saccharomyces cerevisiae*. *Biochim. Biophys. Acta* **1735**, 50–58 (2005).

26. M. J. Olarte, S. Kim, M. E. Sharp, J. M. J. Swanson, R. V. Farese Jr., T. C. Walther, Determinants of endoplasmic reticulum-to-lipid droplet protein targeting. *Dev. Cell* **54**, 471–487.e7 (2020).

27. J. N. Lee, H. Kim, H. Yao, Y. Chen, K. Weng, J. Ye, Identification of Ubxd8 protein as a sensor for unsaturated fatty acids and regulator of triglyceride synthesis. *Proc. Natl. Acad. Sci. U.S.A.* **107**, 21424–21429 (2010).

28. A. Stevanovic, C. Thiele, Monotopic topology is required for lipid droplet targeting of ancient ubiquitous protein 1. *J. Lipid Res.* **54**, 503–513 (2013).

29. S. J. Stone, M. C. Levin, R. V. Farese Jr., Membrane topology and identification of key functional amino acid residues of murine acyl-CoA:diacylglycerol acyltransferase-2. *J. Biol. Chem.* **281**, 40273–40282 (2006).

30. B. M. Abell, L. A. Holbrook, M. Abenes, D. J. Murphy, M. J. Hills, M. M. Moloney, Role of the proline knot motif in oleosin endoplasmic reticulum topology and oil body targeting. *Plant Cell* **9**, 1481–1493 (1997).

31. A. H. Huang, Oleosins and oil bodies in seeds and other organs. *Plant Physiol.* **110**, 1055–1061 (1996).

32. R. G. Hope, D. J. Murphy, J. McLauchlan, The domains required to direct core proteins of hepatitis C virus and GB virus-B to lipid droplets share common features with plant oleosin proteins. *J. Biol. Chem.* **277**, 4261–4270 (2002).

33. J. P. Segrest, H. De Loof, J. G. Dohlman, C. G. Brouillette, G. M. Anantharamaiah, Amphipathic helix motif: Classes and properties. *Proteins* **8**, 103–117 (1990).

34. S. Veit, L. C. Paweletz, T. Günther Pomorski, Determination of membrane protein orientation upon liposomal reconstitution down to the single vesicle level. *Biol. Chem.* **404**, 647–661 (2023).

35. J. D. Lenz, M. B. Lawrenz, D. G. Cotter, M. C. Lane, R. J. Gonzalez, M. Palacios, V. L. Miller, Expression during host infection and localization of *Yersinia pestis* autotransporter proteins. *J. Bacteriol.* **193**, 5936–5949 (2011).

36. A. Saurí, N. Oreshkova, Z. Soprova, W. S. Jong, M. Sani, P. J. Peters, J. Luirink, P. van Ulsen, Autotransporter β-domains have a specific function in protein secretion beyond outer-membrane targeting. *J. Mol. Biol.* **412**, 553–567 (2011).

37. N. Noinaj, A. J. Kuszak, J. C. Gumbart, P. Lukacik, H. Chang, N. C. Easley, T. Lithgow, S. K. Buchanan, Structural insight into the biogenesis of β-barrel membrane proteins. *Nature* **501**, 385–390 (2013).

38. S. D. Schumacher, J. Jose, Expression of active human P450 3A4 on the cell surface of *Escherichia coli* by Autodisplay. *J. Biotechnol.* **161**, 113–120 (2012).

39. W. Kang'ethe, H. D. Bernstein, Stepwise folding of an autotransporter passenger domain is not essential for its secretion. *J. Biol. Chem.* **288**, 35028–35038 (2013).

40. G. H. Han, G. H. Gim, W. Kim, S. I. Seo, S. W. Kim, Enhanced indirubin production in recombinant *Escherichia coli* harboring a flavin-containing monooxygenase gene by cysteine supplementation. *J. Biotechnol.* **164**, 179–187 (2012).

41. A. Wisniewska, J. Widomska, W. K. Subczynski, Carotenoid-membrane interactions in liposomes: Effect of dipolar, monopolar, and nonpolar carotenoids. *Acta Biochim. Pol.* **53**, 475–484 (2006).

42. C. J. Balibar, C. T. Walsh, In vitro biosynthesis of violacein from L-tryptophan by the enzymes VioA-E from *Chromobacterium violaceum*. *Biochemistry* **45**, 15444–15457 (2006).

43. K. S. Ryan, C. J. Balibar, K. E. Turo, C. T. Walsh, C. L. Drennan, The violacein biosynthetic enzyme VioE shares a fold with lipoprotein transporter proteins. *J. Biol. Chem.* **283**, 6467–6475 (2008).

44. H. Park, S. Park, Y.-H. Yang, K.-Y. Choi, Microbial synthesis of violacein pigment and its potential applications. *Crit. Rev. Biotechnol.* **41**, 879–901 (2021).

45. E. M. Zhao, N. Suek, M. Z. Wilson, E. Dine, N. L. Pannucci, Z. Gitai, J. L. Avalos, J. E. Toettcher, Light-based control of metabolic flux through assembly of synthetic organelles. *Nat. Chem. Biol.* **15**, 589–597 (2019).

46. W. C. DeLoache, Z. N. Russ, J. E. Dueber, Towards repurposing the yeast peroxisome for compartmentalizing heterologous metabolic pathways. *Nat. Commun.* **7**, 11152 (2016).

47. P. S. Grewal, J. A. Samson, J. J. Baker, B. Choi, J. E. Dueber, Peroxisome compartmentalization of a toxic enzyme improves alkaloid production. *Nat. Chem. Biol.* **17**, 96–103 (2021).

48. B. J. Johnson, W. R. Algar, A. P. Malanoski, M. G. Ancona, I. L. Medintz, Understanding enzymatic acceleration at nanoparticle interfaces: Approaches and challenges. *Nano Today* **9**, 102–131 (2014).

49. L. J. Sweetlove, A. R. Fernie, The role of dynamic enzyme assemblies and substrate channelling in metabolic regulation. *Nat. Commun.* **9**, 2136 (2018).

50. M. Radulovic, O. Knittelfelder, A. Cristobal-Sarramian, D. Kolb, H. Wolinski, S. D. Kohlwein, The emergence of lipid droplets in yeast: Current status and experimental approaches. *Curr. Genet.* **59**, 231–242 (2013).

51. K. Grillitsch, M. Connerth, H. Kofeler, T. N. Arrey, B. Rietschel, B. Wagner, M. Karas, G. Daum, Lipid particles/droplets of the yeast *Saccharomyces cerevisiae* revisited: Lipidome meets proteome. *Biochim. Biophys. Acta* **1811**, 1165–1176 (2011).

52. B. J. Palmero, E. Gamero, N. M. Mangan, D. Tullman-Ercek, Encapsulation of select violacein pathway enzymes in the 1,2-propanediol utilization bacterial microcompartment to divert pathway flux. *Metab. Eng.* **91**, 91–102 (2025).

53. P. Eastman, R. Galvelis, R. P. Pelaez, C. R. A. Abreu, S. E. Farr, E. Gallicchio, A. Gorenko, M. M. Henry, F. Hu, J. Huang, A. Kramer, J. Michel, J. A. Mitchell, V. S. Pande, J. P. Rodrigues, J. Rodriguez-Guerra, A. C. Simmonett, S. Singh, J. Swails, P. Turner, Y. Wang, I. Zhang, J. D. Chodera, G. De Fabritiis, T. E. Markland, OpenMM 8: Molecular dynamics simulation with machine learning potentials. *J. Phys. Chem. B* **128**, 109–116 (2024).

54. J. B. Klauda, R. M. Venable, J. A. Freites, J. W. O'Connor, D. J. Tobias, C. Mondragon-Ramirez, I. Vorobyov, A. D. MacKerell Jr., R. W. Pastor, Update of the CHARMM all-atom additive force field for lipids: Validation on six lipid types. *J. Phys. Chem. B* **114**, 7830–7843 (2010).

55. R. B. Best, X. Zhu, J. Shim, P. E. Lopes, J. Mittal, M. Feig, A. D. Mackerell Jr., Optimization of the additive CHARMM All-atom protein force field targeting improved sampling of the

backbone ϕ, ψ and side-chain χ_1 and χ_2 dihedral angles. *J. Chem. Theory Comput.* **8**, 3257–3273 (2012).

56. A. D. MacKerell, D. Bashford, M. Bellott, R. L. Dunbrack, J. D. Evanseck, M. J. Field, S. Fischer, J. Gao, H. Guo, S. Ha, D. Joseph-McCarthy, L. Kuchnir, K. Kuczera, F. T. Lau, C. Mattos, S. Michnick, T. Ngo, D. T. Nguyen, B. Prodhom, W. E. Reiher, B. Roux, M. Schlenkrich, J. C. Smith, R. Stote, J. Straub, M. Watanabe, J. Wiorkiewicz-Kuczera, D. Yin, M. Karplus, All-atom empirical potential for molecular modeling and dynamics studies of proteins. *J. Phys. Chem. B* **102**, 3586–3616 (1998).

57. P. Campomanes, J. Prabhu, V. Zoni, S. Vanni, Recharging your fats: CHARMM36 parameters for neutral lipids triacylglycerol and diacylglycerol. *Biophys. Rep.* **1**, 10.1016/j.bpr.2021.100034 (2021).

58. R. J. Braun, J. M. J. Swanson, Progress in lipid droplet simulations: How polarizability shapes triacylglycerol behavior in bulk and interfacial environments. *J. Chem. Theory Comput.* **21**, 8235–8248 (2025).

59. S. Kim, J. M. J. Swanson, G. A. Voth, Computational studies of lipid droplets. *J. Phys. Chem. B* **126**, 2145–2154 (2022).

60. M. J. Tol, Y. Shimanaka, A. H. Bedard, J. Sapia, L. Cui, M. Colaco-Gaspar, P. Hofer, A. Ferrari, K. Qian, J. P. Kennelly, S. D. Lee, Y. Gao, X. Xiao, J. Gao, J. J. Mack, T. A. Weston, K. J. Williams, B. Su, C. Pan, A. J. Lusis, D. P. Pike, A. Reed, N. Milosevich, B. F. Cravatt, M. Arita, S. G. Young, D. A. Ford, R. Zechner, S. Vanni, P. Tontonoz, Dietary control of peripheral adipose storage capacity through membrane lipid remodelling. *Nat. Metab.* **7**, 1424–1442 (2025).

61. B. A. Berger, H. M. Vietor, D. W. Scott, H. Lee, S. Hashemipour, W. Im, N. J. Wittenberg, K. J. Glover, Physicochemical properties of seed oil blends and their potential for the creation of synthetic oleosomes with modulated polarities. *ACS Omega* **9**, 43193–43202 (2024).

62. K. Vanommeslaeghe, E. Hatcher, C. Acharya, S. Kundu, S. Zhong, J. Shim, E. Darian, O. Guvench, P. Lopes, I. Vorobyov, A. D. Mackerell Jr., CHARMM general force field: A force

field for drug-like molecules compatible with the CHARMM all-atom additive biological force fields. *J. Comput. Chem.* **31**, 671–690 (2010).

63. T. Darden, D. York, L. Pedersen, Particle mesh Ewald: An *N*·log (*N*) method for Ewald sums in large systems. *J. Chem. Phys.* **98**, 10089–10092 (1993).

64. U. Essmann, L. Perera, M. L. Berkowitz, T. Darden, H. Lee, L. G. Pedersen, A smooth particle mesh Ewald method. *J. Chem. Phys.* **103**, 8577–8593 (1995).

65. J. Lee, X. Cheng, S. Jo, A. D. MacKerell Jr., J. B. Klauda, W. Im, CHARMM-GUI input generator for NAMD, Gromacs, Amber, Openmm, and CHARMM/OpenMM simulations using the CHARMM36 additive force field. *Biophys. J.* **110**, 641a (2016).

66. S. Jo, T. Kim, V. G. Iyer, W. Im, CHARMM-GUI: A web-based graphical user interface for CHARMM. *J. Comput. Chem.* **29**, 1859–1865 (2008).

67. J. Jumper, R. Evans, A. Pritzel, T. Green, M. Figurnov, O. Ronneberger, K. Tunyasuvunakool, R. Bates, A. Zidek, A. Potapenko, A. Bridgland, C. Meyer, S. A. A. Kohl, A. J. Ballard, A. Cowie, B. Romera-Paredes, S. Nikolov, R. Jain, J. Adler, T. Back, S. Petersen, D. Reiman, E. Clancy, M. Zielinski, M. Steinegger, M. Pacholska, T. Berghammer, S. Bodenstein, D. Silver, O. Vinyals, A. W. Senior, K. Kavukcuoglu, P. Kohli, D. Hassabis, Highly accurate protein structure prediction with AlphaFold. *Nature* **596**, 583–589 (2021).

68. G. M. Torrie, J. P. Valleau, Nonphysical sampling distributions in Monte Carlo free-energy estimation: Umbrella sampling. *J. Comput. Phys.* **23**, 187–199 (1977).

69. F. Zhu, G. Hummer, Convergence and error estimation in free energy calculations using the weighted histogram analysis method. *J. Comput. Chem.* **33**, 453–465 (2012).

70. W. M. Haynes, *CRC Handbook of Chemistry and Physics* (CRC Press, 2016).

71. R. D. Gietz, R. H. Schiestl, High-efficiency yeast transformation using the LiAc/SS carrier DNA/PEG method. *Nat. Protoc.* **2**, 31–34 (2007).

72. N. W. Rigel, D. P. Ricci, T. J. Silhavy, Conformation-specific labeling of BamA and suppressor analysis suggest a cyclic mechanism for β-barrel assembly in *Escherichia coli*. *Proc. Natl. Acad. Sci. U.S.A.* **110**, 5151–5156 (2013).

73. V. Tatzer, G. Zellnig, S. D. Kohlwein, R. Schneiter, Lipid-dependent subcellular relocalization of the acyl chain desaturase in yeast. *Mol. Biol. Cell* **13**, 4429–4442 (2002).

74. R. Köffel, R. Tiwari, L. Falquet, R. Schneiter, The *Saccharomyces cerevisiae YLL012/YEH1*, *YLR020/YEH2*, and *TGL1* genes encode a novel family of membrane-anchored lipases that are required for steryl ester hydrolysis. *Mol. Cell. Biol.* **25**, 1655–1668 (2005).

75. J. DeCaprio, T. O. Kohl, Lysing yeast cells with glass beads for immunoprecipitation. *Cold Spring Harb. Protoc.* **2020**, 10.1101/pdb.prot098590 (**2020**).

76. J. L. Lin, J. Zhu, I. Wheeldon, Synthetic protein scaffolds for biosynthetic pathway colocalization on lipid droplet membranes. *ACS Synth. Biol.* **6**, 1534–1544 (2017).

77. M. Çağlayan, S. H. Wilson, Enzymatic activity assays in yeast cell extracts. *Bio Protoc.* **4**, e1312 (2014).

78. M. E. Lee, A. Aswani, A. S. Han, C. J. Tomlin, J. E. Dueber, Expression-level optimization of a multi-enzyme pathway in the absence of a high-throughput assay. *Nucleic Acids Res.* **41**, 10668–10678 (2013).

79. D. Yang, S. Y. Park, S. Y. Lee, Production of rainbow colorants by metabolically engineered *Escherichia coli*. *Adv. Sci. (Weinh)* **8**, e2100743 (2021).

80. J. E. Kim, I. S. Jang, S. H. Son, Y. J. Ko, B. K. Cho, S. C. Kim, J. Y. Lee, Tailoring the *Saccharomyces cerevisiae* endoplasmic reticulum for functional assembly of terpene synthesis pathway. *Metab. Eng.* **56**, 50–59 (2019).

81. L. Frusciante, P. Carli, M. R. Ercolano, R. Pernice, A. Di Matteo, V. Fogliano, N. Pellegrini, Antioxidant nutritional quality of tomato. *Mol. Nutr. Food Res.* **51**, 609–617 (2007).

82. W. Kang, T. Ma, M. Liu, J. L. Qu, Z. J. Liu, H. W. Zhang, B. Shi, S. Fu, J. C. Ma, L. T. F. Lai, S. C. He, J. A. Qu, S. W. N. Au, B. H. Kang, W. C. Y. Lau, Z. X. Deng, J. Xia, T. G. Liu, Modular enzyme assembly for enhanced cascade biocatalysis and metabolic flux. *Nat. Commun.* **10**, 4248 (2019).

83. J. F. Rebbeor, G. C. Connolly, M. E. Dumont, N. Ballatori, ATP-dependent transport of reduced glutathione in yeast secretory vesicles. *Biochem. J.* **334** (Pt. 3), 723–729 (1998).

84. M. A. Rasheed, R. Mohy-Ud-Din, T. Anwar, M. Faiz, A novel cell biological tool to explain mechanics and dynamics in fission yeast. *J. Basic Microbiol.* **64**, e2300605 (2024).

85. S. Chen, P. Novick, S. Ferro-Novick, ER network formation requires a balance of the dynamin-like GTPase Sey1p and the Lunapark family member Lnp1p. *Nat. Cell Biol.* **14**, 707–716 (2012).

86. Y. Ohsaki, T. Maeda, T. Fujimoto, Fixation and permeabilization protocol is critical for the immunolabeling of lipid droplet proteins. *Histochem. Cell Biol.* **124**, 445–452 (2005).

87. T. Exner, I. Romero-Brey, E. Yifrach, J. Rivera-Monroy, B. Schrul, C. C. Zouboulis, W. Stremmel, M. Honsho, R. Bartenschlager, E. Zalckvar, M. Poppelreuther, J. Füllekrug, An alternative membrane topology permits lipid droplet localization of peroxisomal fatty acyl-CoA reductase 1. *J. Cell Sci.* **132**, jcs223016 (2019).

88. K. N. Richardson, W. B. Black, H. Li, Aldehyde production in crude lysate- and whole cell-based biotransformation using a noncanonical redox cofactor system. *ACS Catal.* **10**, 8898–8903 (2020).

89. S. L. Hooe, M. Thakur, G. Lasarte-Aragones, J. C. Breger, S. A. Walper, I. L. Medintz, G. A. Ellis, Exploration of the in vitro violacein synthetic pathway with substrate analogues. *ACS Omega* **9**, 3894–3904 (2024).

90. R. Nagel, J. Gershenzon, A. Schmidt, Nonradioactive assay for detecting isoprenyl diphosphate synthase activity in crude plant extracts using liquid chromatography coupled with tandem mass spectrometry. *Anal. Biochem.* **422**, 33–38 (2012).

91. P. J. Chitwood, S. Juszkiewicz, A. Guna, S. Shao, R. S. Hegde, EMC is required to initiate accurate membrane protein topogenesis. *Cell* **175**, 1507–1519.e16 (2018).

92. H. Nishikawa, M. Sasaki, K.-i. Nishiyama, In vitro assay for bacterial membrane protein integration into proteoliposomes. *Bio Protoc.* **10**, e3626 (2020).

93. J. Y. Lee, C. D. Kang, S. H. Lee, Y. K. Park, K. M. Cho, Engineering cellular redox balance in *Saccharomyces cerevisiae* for improved production of L-lactic acid. *Biotechnol. Bioeng.* **112**, 751–758 (2015).

94. L. Hochrein, L. A. Mitchell, K. Schulz, K. Messerschmidt, B. Mueller-Roeber, L-SCRaMbLE as a tool for light-controlled Cre-mediated recombination in yeast. *Nat. Commun.* **9**, 1931 (2018).

95. S. R. Edwards, T. J. Wandless, Dicistronic regulation of fluorescent proteins in the budding yeast *Saccharomyces cerevisiae*. *Yeast* **27**, 229–236 (2010).

96. F. Farzadfard, S. D. Perli, T. K. Lu, Tunable and multifunctional eukaryotic transcription factors based on CRISPR/Cas. *ACS Synth. Biol.* **2**, 604–613 (2013).
